# Structural Basis for BD1-Preferring 2,4-Disubstituted Pyrimidine BRDT Inhibitors

**DOI:** 10.1021/acs.jmedchem.6c00180

**Published:** 2026-04-15

**Authors:** Taimeng Liang, Xianghong Guan, Alice Chan, Prakriti Kalra, Rui Shi, Jonathan Solberg, Logan H. Sigua, Jun Qi, William C. K. Pomerantz, Ernst Schönbrunn, Jon E. Hawkinson, Gunda I. Georg

**Affiliations:** † Department of Chemistry, 5635 University of Minnesota, 207 Pleasant Street, SE, Minneapolis, Minnesota 55455-0431, United States; ‡ Department of Medicinal Chemistry and Institute for Therapeutics Discovery and Development, University of Minnesota College of Pharmacy, 717 Delaware Street, SE, Minneapolis, Minnesota 55414, United States; § Drug Discovery Department, Moffitt Cancer Center, 12902 Magnolia Drive, Tampa, Florida 33612, United States; ∥ Department of Medical Oncology, Dana-Farber Cancer Institute, and Department of Medicine, 1855 Harvard Medical School, 360 Longwood Avenue, Boston, Massachusetts 02215, United States

## Abstract

The first bromodomain of the BET protein BRDT (BRDT-BD1) possesses a unique Arg54 residue at the terminus of the ZA channel, absent in other BET family members. We explored this structural uniqueness with 23 analogs of the BET/kinase inhibitor **SG3–179**, each bearing an amino acid side chain to enable potential interactions between the positively charged arginine group and the negatively charged carboxylate groups. In an AlphaScreen assay, serine analog **13** showed 35-fold selectivity for BRDT-T over BRD4-T. The BRDT-BD1 cocrystal structure with glutamic acid analog **14** showed no interaction with Arg54, suggesting that the observed preference may be related to differences in the structured water molecules. Compound **13** displayed exceptional in vitro metabolic stability but had limited cellular permeability in MDCK-MDR1 cells. Compounds **13** and **14** are among the best BRDT-BD1-preferring inhibitors reported to date and demonstrate a significant step toward identifying highly selective BRDT inhibitors for male contraception.

## Graphical abstract

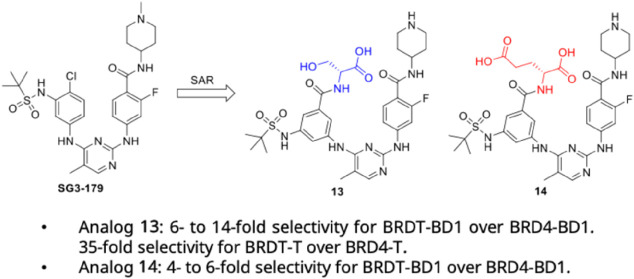

## Introduction

The development of effective contraceptive methods is a crucial public health priority, yet contraceptive options for men remain extremely limited.^1–3^ While women have had access to oral contraceptives for over five decades, male contraceptive methods are currently restricted to vasectomy and condoms, both of which have significant limitations due to lack of efficacy or lack of reversibility.^4^ The lack of effective male-directed contraceptive options contributes to high rates of unintended pregnancies, leading to substantial social and economic consequences.^5^ Extensive global studies have evaluated male hormonal contraceptives, including testosterone derivatives, progestin combination therapies, and varied drug formulations.^3,4^ While these approaches demonstrate efficacy and reversibility, they face significant translational barriers. Exogenous testosterone administration reduces high-density lipoprotein (HDL) cholesterol levels, a known cardiovascular risk factor.^6^ Additional challenges include ethnic variability in therapeutic response,^7,8^ adverse effects such as mood disturbances,^9^ acne, weight gain, and unresolved concerns about long-term prostate safety.^10^ Collectively, these limitations have hindered the development of a commercially viable male hormonal contraceptive agent. A nonhormonal, reversible male contraceptive would represent a significant advance in reproductive health by expanding contraceptive choices and promoting reproductive autonomy for both men and women.

A promising target for nonhormonal male contraception is the bromodomain testis-specific protein (BRDT), a member of the bromodomain and extra-terminal (BET) subfamily of epigenetic reader proteins (Figure 1a).^11^ BET proteins comprise four structurally similar proteins: BRD2, BRD3, BRD4, and BRDT. Each member contains two distinct bromodomains (BD1 and BD2) located near the N-terminus, along with a characteristic extra-terminal (ET) motif at the C-terminal. The bromodomains of BET proteins are highly conserved and exhibit a strong affinity for hyperacetylated lysine (Kac) residues on the histone tails H3 and H4 (Figure 1b), playing a crucial role in epigenetic regulation and transcriptional control.^12–14^ Unlike other BET proteins, which are widely expressed in somatic tissues, BRDT is exclusively expressed in the testis and plays an essential role in spermatogenesis.^15,16^ It is required for meiotic gene expression and postmitotic histone removal, enabling chromatin condensation and sperm maturation.^17–19^ Genetic studies have confirmed BRDT’s crucial role in male fertility; mice lacking the first bromodomain of BRDT (Brdt^ΔBD1^) are completely infertile due to defects in spermiogenesis,^20^ while complete deletion of BRDT leads to sterility with additional defects in meiotic prophase.^16^ Further validation comes from genome-wide association studies (GWAS), which have associated BRDT single-nucleotide polymorphisms (SNPs) with oligozoospermia, suggesting a functional role in human male fertility.^21^ Since BRDT-BD1 deletion alone is sufficient to cause sterility in mice, targeting BRDT-BD1 specifically could yield a highly selective and reversible male contraceptive with minimal off-target effects.

**Figure 1:**
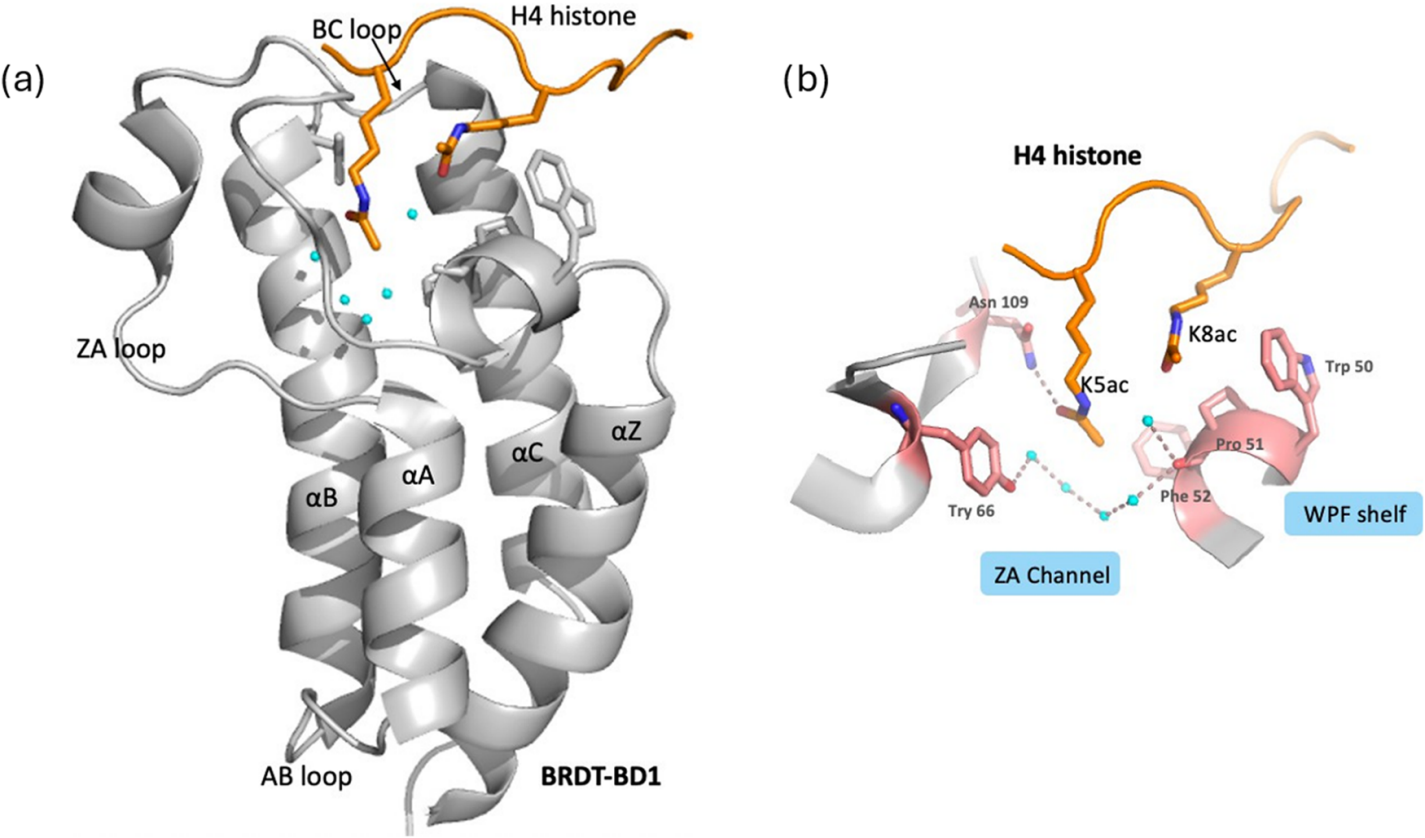
(a) Structure of BRDT- BD1 (PDB: 2WP2). (b) Kac pocket of BD1 in BRDT with the orientation of the histone subunit H4 protein. Cyan spheres represent five conserved water molecules observed within the Kac binding site.

The pharmacological feasibility of BRDT inhibition as a nonhormonal contraceptive strategy was first demonstrated using (+)-JQ1, a pan-BET inhibitor (Figure 2). (+)-JQ1 effectively crosses the blood-testis barrier, inhibiting BRDT function in spermatocytes and spermatids, and leading to reduced sperm count and motility in mice, resulting in reversible male infertility without altering hormone levels.^15,22^ Although (+)-JQ1 is not isoform- or domain-selective and BRD2, BRD3, and BRD4 are also expressed in the testis,^23^ the infertility phenotype closely recapitulates that observed in Brdt genetic knockout models, including impaired spermatogenesis and defective chromatin remodeling in postmeiotic germ cells. BRDT is highly enriched in pachytene spermatocytes and round spermatids, where it plays a central role in chromatin compaction during spermiogenesis.^24^ The reversible sterility induced by (+)-JQ1, together with the testis-restricted expression pattern and established genetic validation of BRDT, supports the interpretation that BRDT inhibition is a principal contributor to the observed contraceptive phenotype, although contributions from other BET paralogs cannot be excluded. However, despite its utility as a proof-of-concept molecule, (+)-JQ1 has significant limitations for contraceptive development. (+)-JQ1 lacks selectivity, where it exhibits a higher binding affinity for BRD4 over BRDT, thus leading to potential off-target effects as BRD4 is expressed in somatic cells.^25,26^

**Figure 2:**
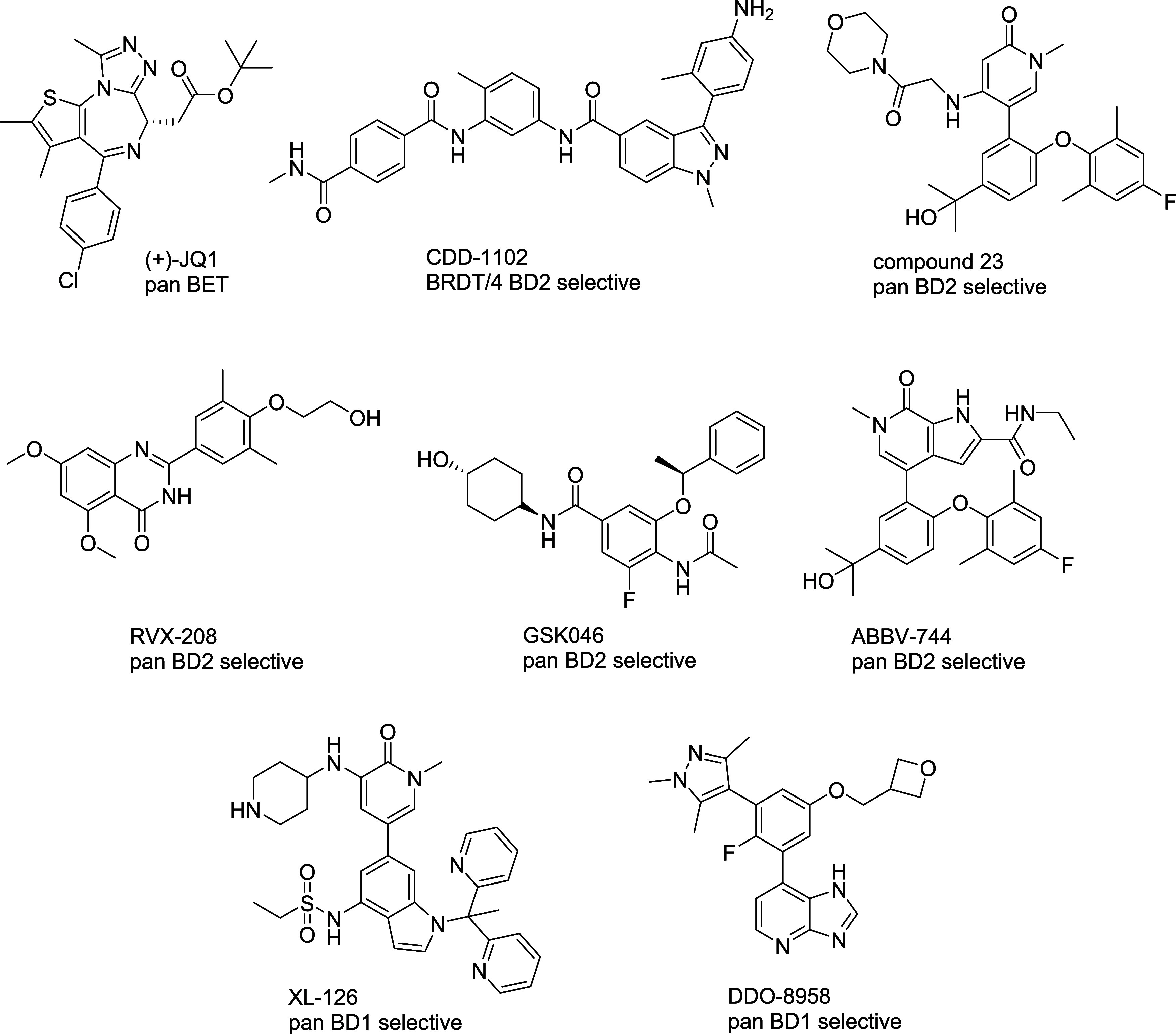
Chemical structures of exemplary BET bromodomain inhibitors.

Efforts to improve BET inhibitor selectivity have resulted in the discovery of numerous small molecules (Figure 2). From DNA-encoded chemical library screening performed by Matzuk et al., **CDD-1102** was found as a BRDT-BD2-selective inhibitor with an IC_50_ of 7 nM and >1000-fold selectivity over BRDT-BD1.^27^ This compound was also BD2-selective for BRD4 (IC_50_ of 4460 nM for BRD4-BD1 and IC_50_ of 15 nM for BRD4-BD2). However, because **CDD-1102** was not evaluated against BRD2 and BRD3, its selectivity cannot be assessed across the full BET family and is therefore described here as BRDT/BRD4 BD2-selective.

Additionally, advances in BET inhibitor research for cancer and inflammatory diseases support the feasibility of bromodomain-selective targeting.^28,29^ Compound **23** (Figure 2), a pan-BD2- inhibitor, exhibited a remarkable IC_50_ of 2.9 nM for BRD4-BD2 and 2,583-fold selectivity over BRD4-BD1, while maintaining 453- to 1214-fold BD2/BD1 selectivity across BRD2, BRD3, and BRDT.^30^ Early BD2-preferring inhibitors such as **RVX-208** (Figure 2) showed an up to 23-fold preference for BD2 over BD1 across BET proteins (*K*_D_ of 195 nM against BD2 and 4 μM against BD1 for BRD3), demonstrating the feasibility of domain preference, although their potency and selectivity were modest and assay-dependent.^29,31^ Subsequent development of more potent and selective pan-BD2 inhibitors provided clearer evidence for domain-selective inhibition of BET biology. For example, the discovery of **GSK046** (Figure 2), a BD2-selective inhibitor (>300-fold selectivity over BD1), revealed a critical role for BD2 in gene regulation and inflammation and provided evidence that BET proteins primarily bind chromatin via BD1, implying that BD1-selective inhibition could reduce off-target effects.^32,33^ Similarly, **ABBV-744** (Figure 2), a BD2-selective BET inhibitor (95 to 290-fold selectivity for BRD4-BD2) that has advanced to clinical evaluation for AML and prostate cancer (NCT03360006, NCT04454658),^34^ demonstrated improved tolerability and anticancer efficacy,^35^ reinforcing the importance of selective bromodomain inhibition in drug development.

In parallel with BD2-selective efforts, significant progress has also been made in developing BD1-selective BET inhibitors. Structure-guided optimization of a pyridinone-based Kac-mimetic scaffold led to the identification of **XL-126** (Figure 2), which binds BRD4-BD1 with a *K*_D_ of 8.9 nM and exhibits 185-fold selectivity over BRD4-BD2, with broader BROMOscan profiling demonstrating substantial discrimination against BD2 domains across the BET family.^36^ More recently, a multiwater-bridge design strategy yielded **DDO-8958**, a potent pan-BD1 inhibitor that binds BRD4-BD1 with a *K*_D_ of 5.6 nM and displays approximately 214-fold selectivity for BD1 over BD2 (Figure 2).^37^ The high sequence and structural similarity among BD1 domains, and separately among BD2 domains,^38^ makes selective targeting of a single domain within a single BET paralog particularly challenging. Consequently, many reported selective inhibitors are pan-BD1 or pan-BD2 rather than paralog-specific.

Despite significant progress in BET inhibitor design, selective BRDT-BD1 inhibitors have not yet been reported. This may be partly attributable to the fact that most BET inhibitor research has focused on oncology and inflammatory diseases, where BRDT activity is often not explicitly evaluated, making it difficult to determine whether BRDT-preferring inhibitors have been identified. To identify potent and selective BRDT-BD1 inhibitors, we turned to the known kinase inhibitor **TG101209** (Figure 3a), which has shown notable activity against BRDT and demonstrates dual targeting of kinases and bromodomains.^39–41^ Given that both BRD4 and various kinases play central roles in mitotic regulation, a dual kinase-bromodomain inhibition strategy has been proposed to combat drug resistance associated with kinase inhibition. Early pharmacological studies have shown that acute myeloid leukemia (AML) cells resistant to kinase inhibitors remain sensitive to BRD4 inhibition,^42^ highlighting the therapeutic potential of targeting both pathways simultaneously.

**Figure 3:**
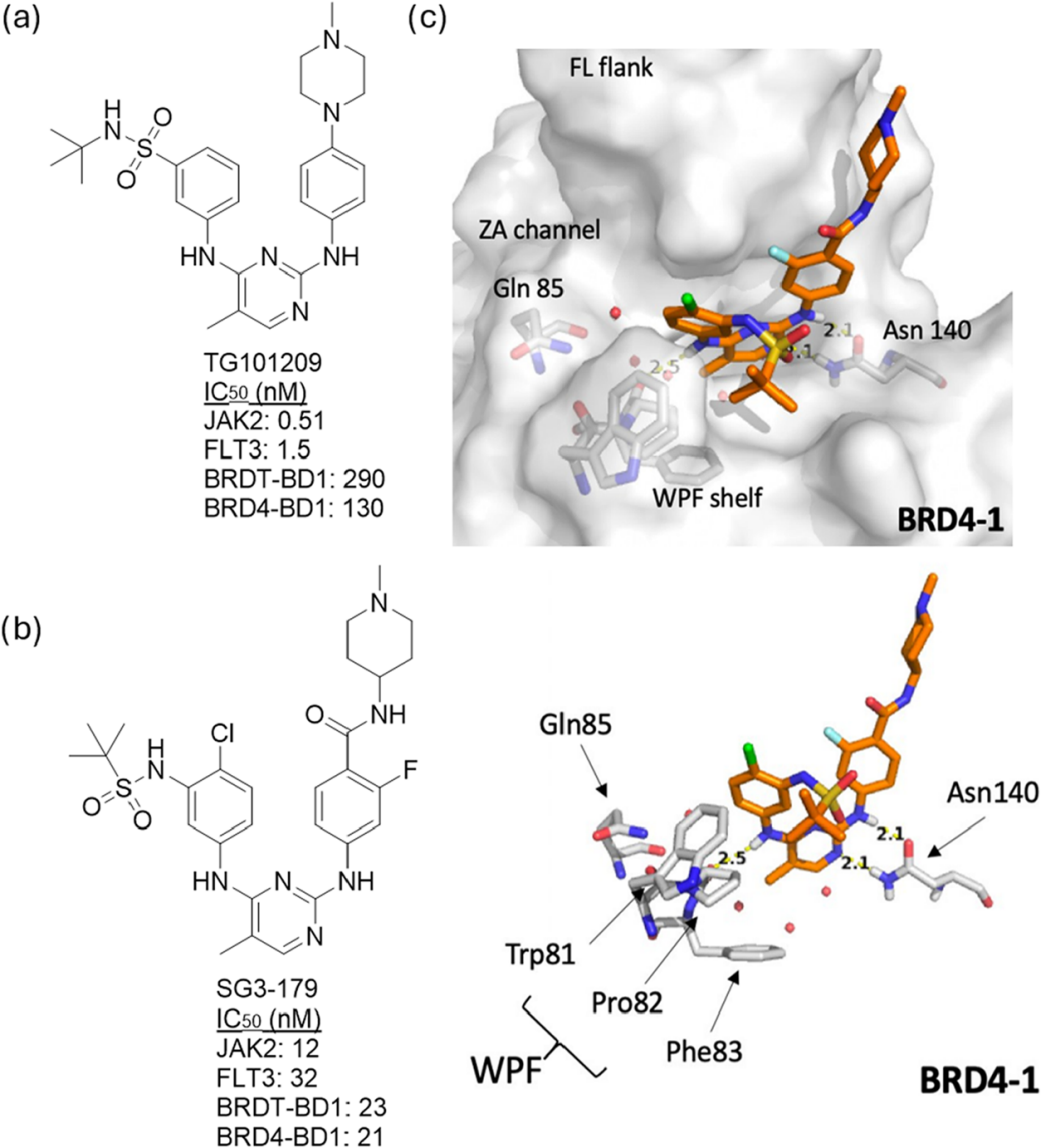
(a) Chemical structure of kinase inhibitor **TG101209** and its activity for JAK2, FLT3, BRDT-BD1, and BRD4-BD1. (b) Chemical structure of BET/kinase inhibitor **SG3–179** and its activity for JAK2, FLT3, BRDT-BD1, and BRD4-BD1. (c) Co-crystal structure of BRD4-BD1 with **SG3–179** within the Kac binding site (PDB: 5F63). Hydrogen bonds are formed with Asn140 and Pro82 and are highlighted in yellow. Red spheres represent the conserved water molecules found in the Kac binding site.

**TG101209** is a known Janus kinase 2 (JAK2) inhibitor with an IC_50_ of 0.51 nM derived from an AlphaScreen assay.^43^ It also inhibits fms-like tyrosine kinase 3 (FLT3) with an IC_50_ of 1.5 nM. Furthermore, it inhibits BRDT-BD1 and BRD4-BD1 with an IC_50_ of 290 nM and 130 nM, respectively.^44^ Activity of **TG101209** against BRD2 and BRD3 bromodomains was not reported in the referenced studies; therefore, its selectivity across the full BET family cannot be assessed from the available data. Structural studies by Schönbrunn et al. have provided insights into its dual activity, revealing that **TG101209** binds to BRD4-BD1 at the acetyl-lysine (Kac) binding site, where its pyrimidine nitrogen and adjacent amine group mimic the interaction of acetylated histone H4K5ac, forming a hydrogen bond network with the conserved asparagine (Asn140) in BRD4-BD1.^40^ A comparable binding mode was observed in the cocrystal structure of **TG101209** with JAK2 (PDB: 4JI9), where its aminopyrimidine moiety forms hydrogen bonds with Leu932 in the hinge region of JAK2.^41^

These findings provided a rationale for further structural modifications of **TG101209**, ultimately leading to the development of **SG3–179** (Figure 3c), a 2,4-disubstituted pyrimidine derivative with an IC_50_ of 12 nM and 32 nM for JAK2 and FLT3, respectively.^44^ **SG3–179** also displays potent pan-BET bromodomain inhibition, with improved submicromolar activity against BET family proteins, with IC_50_ values of 23 nM and 21 nM for BRDT-BD1 and BRD4-BD1, respectively.^44,46^ Specifically, **SG3–179** was selected as a starting point due to the availability of high-resolution cocrystal structures and its well-characterized pan-BET profile, which provided a structurally validated scaffold for rational modification. Although the parent compound exhibits kinase activity, our design strategy specifically aimed to eliminate kinase inhibition while preserving bromodomain engagement. In the cocrystal structure of BRD4-BD1 with **SG3–179** (Figure 3b), the pyrimidine core and the NH linker between the pyrimidine ring and the fluorine-substituted benzene ring establish a hydrogen bond network with the conserved Asn140, a key residue in the bromodomain’s acetyl-lysine recognition site. Additionally, the NH linker between the aryl ring containing the *tert*-butyl sulfonamide moiety and the pyrimidine ring forms a hydrogen bond with Pro82, a component of the tryptophan-proline-phenylalanine (WPF) shelf, a critical structural motif in BET bromodomains. The *tert*-butyl group is positioned within the hydrophobic cavity of the WPF shelf, enhancing ligand stability, while the piperidine moiety extends into the solvent-exposed region, potentially influencing ligand solubility and pharmacokinetic properties.

There is a high degree of structural similarity among BET family members, which has complicated efforts to design inhibitors that effectively distinguish between BRD2, BRD3, BRD4, and BRDT.^11,47,48^ Structurally, all BET proteins share a conserved fold consisting of four α-helices (αZ, αA, αB, αC) connected by two variable loop regions (ZA and BC loops) as shown in Figure 1a. However, BRDT-BD1 possesses a unique Arg54 residue at the terminus of the ZA channel, which is absent in other BET family members (Figure 4). This structural uniqueness offers a promising approach for designing selective BRDT-BD1 inhibitors.^49^ Furthermore, in the first bromodomains of BRDT and BRD4, Arg54 in BRDT-BD1 occupies the position corresponding to Gln85 in BRD4-BD1 (Figure 4b), representing a key residue difference within the ZA channel that may be exploited for selective targeting.

**Figure 4:**
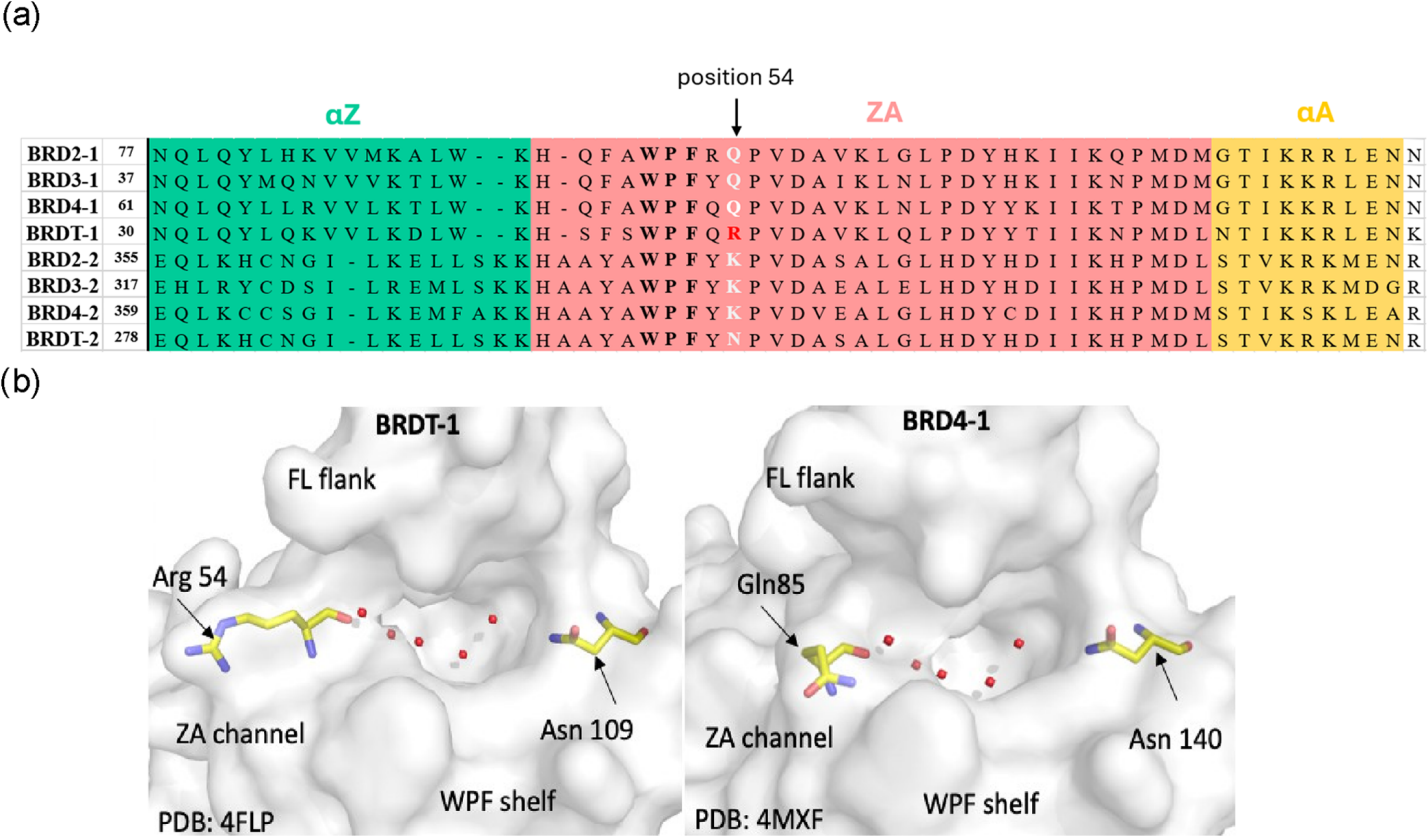
(a) Sequence homology of bromodomains in the BET family presented up to α helix A. α helices Z and A are highlighted in green and yellow, respectively; the ZA loop area is highlighted in pink; Residues in position 54 are indicated where the red residue is the unique arginine residue in BRDT-BD1 (CLUSTAL multiple sequence alignment by Kalign 2.0).^50^ (b) Kac binding pocket of BRDT-BD1 (PDB: 4FLP) and BRD4-BD1 (PDB: 4MXF), where the positions of Arg54 and Gln85 are indicated as they share similar positions within the binding pockets.

Our group and others have previously explored 2,4-disubstituted pyrimidine scaffolds as dual BET/kinase inhibitors, establishing that (i) preservation of the aminopyrimidine–Asn hydrogen bond network is critical for bromodomain affinity, (ii) hydrophobic engagement of the WPF shelf through *tert*-butyl sulfonamide substitution is required for potency, and (iii) substitution at the meta-position relative to the sulfonamide is tolerated and projects toward the ZA channel.^40,44^ These earlier SAR results suggested that the meta-position could serve as a strategic vector for introducing polar functionality without compromising core binding interactions. In the present study, we leveraged this trajectory to introduce amino acid-derived side chains designed to target the unique Arg54 residue of BRDT-BD1.

## Results and Discussion

We designed our BRDT-BD1 inhibitors based on the known binding pose of **SG3–179** in BRD4-BD1 (PDB: 5F63).^51^ The hydrophobic *tert*-butylsulfonamide group is engaged favorably with the WPF shelf, and this lipophilic pharmacophore would be essential for preserving binding affinity in subsequent analogs, as it contributes to complex stability through complementary van der Waals contacts with this hydrophobic protein. To exploit the binding pocket’s chemical topology, we focused on functionalizing the meta position relative to the sulfonamide group, which has a favorable trajectory toward the ZA channel and Gln85 (Figure 3). To target Arg54 within the ZA channel of the binding pocket, side chains were introduced at the meta-position relative to the *tert*-butylsulfonamide group. As an initial evaluation, we utilized the FP assay with (+)-JQ1 as a positive control (SI, Figure S1).

Our streamlined synthetic route, comprising eight steps, is outlined in Scheme 1. The process began with the methylation of commercially available 3,5-diaminobenzoic acid using MeOH, yielding intermediate **1**. This was followed by a single substitution reaction with 2,4-dichloro-5-methylpyrimidine to produce chloropyrimidine **2**. The reaction of intermediate **2** with *tert*-butylsulfinyl chloride generated *tert*-butylsulfinyl-containing intermediate **3**, which was subsequently oxidized with mCPBA to form sulfonamide-containing intermediate **4**.

**Scheme 1:**
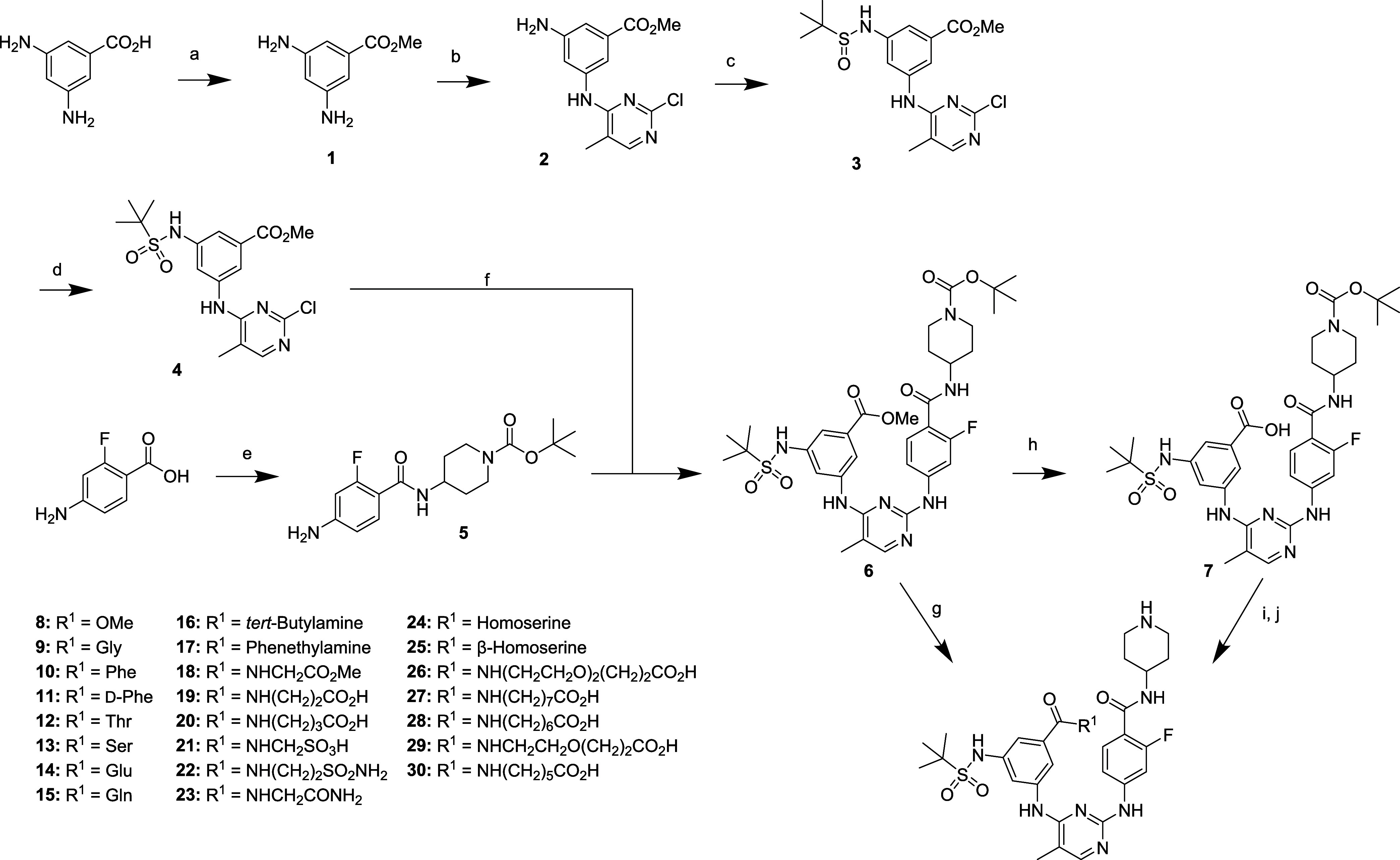
Reaction Scheme for the Synthesis of **SG3-179** Analogs with Meta-Linked Side Chains^a^

a Reagents and conditions: (a) 3,5-diaminobenzoic acid (1 equiv), H_2_SO_4_ (2.5 equiv), MeOH, 0 °C to RT, 5 h, 91%; (b) methyl 3,5-diaminobenzoate (**1**, 1 equiv), 2,4-dichloro-5-methylpyrimidine (1 equiv), DIPEA (3.9 equiv), *n*-BuOH, 120 °C, 24 h, 69%; (c) methyl 3-amino-5-((2-chloro-5-methylpyrimidin-4-yl)­amino)­benzoate (**2**, 1 equiv), *tert-*butylsulfinyl chloride (1 equiv), pyridine, DCM, 0 °C to RT, 20 h, 89%; (d) methyl 3-((*tert*butylsulfinyl)­amino)-5-((2-chloro-5-methylpyrimidin-4-yl)­amino)­benzoate (**3**, 1 equiv), mCPBA (1 equiv), DCM, 0 °C to RT, 81%; (e) 4-amino-2-fluorobenzoic acid (1 equiv), HATU (1.1 equiv), DIPEA (2.0 equiv), *tert*-butyl 4-aminopiperidine-1-carboxylate (1.2 equiv), DMF, RT, 12 h, 95%; (f) *tert*-butyl 4-(4-amino-2-fluorobenzamido)­piperidine-1-carboxylate (**5**, 1 equiv), methyl 3-((2-chloro-5-methylpyrimidin-4-yl)­amino)-5-((1,1-dimethylethyl)­sulfonamido)­benzoate (**4**, 1 equiv), sodium *tert*-butoxide (2 equiv) Pd_2_(dba)_3_ (0.05 equiv), BINAP (0.1 equiv),1,4-dioxane, 110 °C, 7 h, 64%; (g) Intermediate **6** (1 equiv), TFA, DCM, RT, 2 h, 94%; (h) Intermediate **6** (1 equiv), LiOH (3 equiv),MeOH: THF: H_2_O (1:1:2), 60 °C, 3 h, 57%; (i) Intermediate **7** (1 equiv), HATU (1.1 equiv), DIPEA (2.0 equiv), *tert*-butyl ester amine (1.2 equiv), DMF, RT, 6 h; (j) Crude intermediates **7** (1 equiv), TFA, DCM, RT, 2 h, 59–91%.

Fragment **5** was then coupled with intermediate **4** via a Buchwald-Hartwig coupling reaction, resulting in compound **6**. Direct deprotection of the Boc group with TFA yielded the final compound **8** (R^1^ = OMe). Hydrolysis of intermediate **6** with LiOH provided the key intermediate **7**. Various side chains were introduced by coupling **7** with amino acid *tert*-butyl esters or primary amine-containing side chains. These crude intermediates were then subjected to global deprotection with TFA, yielding the final desired target molecules **8–30**.

Table 1 summarizes the SAR for BRDT-BD1 versus BRD4-BD1 in our FP format as an initial selectivity readout. Broader BET family profiling for leads was performed by BROMOscan, as shown below. We first explored methyl ester **8** (Table 1), which exhibits moderate binding affinity for BRDT-BD1 (*K_i_* = 22 nM) and BRD4-BD1 (*K_i_* = 43 nM), with a selectivity ratio of 1.9, indicating a preference for BRDT-BD1. Glycine analog **9** exhibits a slightly reduced affinity for BRDT-BD1 (*K_i_* = 41 nM) and BRD4-BD1 (*K_i_* = 44 nM), with a minimal selectivity index of 1.1.

**Table 1:**
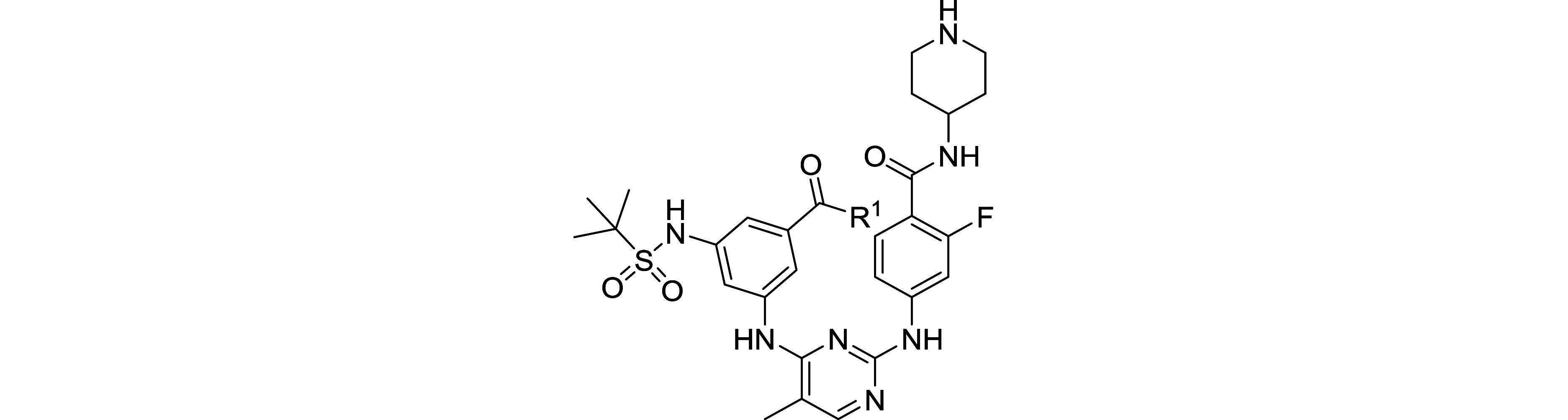
SG3-179 Analogs Where the Meta Position to the *tert*-Butylsulfonamide Group Is Oriented towards the Position of Arg54^a^

		*K_i_* (nM)^b^		
compound	R^1^	BRDT-BD1	BRD4-BD1	selectivity
**(+)-JQ1**		6.3 ± 1.1	6.3 ± 1.1	1.0
**8**	OMe	22 ± 2	43 ± 2	1.9
**9**	Gly	41 ± 1	44 ± 1	1.1
**10**	Phe	28 ± 2	63 ± 2	2.2
**11**	d-Phe	56 ± 1	105 ± 2	1.9
**12**	Thr	27 ± 2	89 ± 1	3.3
**13**	Ser	8.8 ± 1	55 ± 1	6.3
**14**	Glu	10 ± 1	43 ± 1	4.2
**15**	Gln	8.7 ± 0.2	7.6 ± 1	0.87
**16**	*tert*-Butylamine	94 ± 3	101 ± 3	1.1
**17**	Phenethylamine	46 ± 3	58 ± 2	1.3
**18**	NHCH_2_CO_2_Me	26 ± 2	42 ± 1	1.6
**19**	NH(CH_2_)_2_CO_2_H	27 ± 2	49 ± 2	1.8
**20**	NH(CH_2_)_3_CO_2_H	41 ± 4	47 ± 3	1.1
**21**	NHCH_2_SO_3_H	32 ± 3	56 ± 2	1.8
**22**	NH(CH_2_)_2_SO_2_NH_2_	22 ± 0.6	44 ± 0.8	2.0
**23**	NHCH_2_CONH_2_	54 ± 0.6	93 ± 1	1.7
**24**	Homoserine	8.3 ± 1	32 ± 1	3.8
**25**	β-Homoserine	21 ± 3.1	41 ± 2	2.0
**26**	NH(CH_2_CH_2_O)_2_(CH_2_)_2_CO_2_H	39 ± 3	117 ± 4	3.0
**27**	NH(CH_2_)_7_CO_2_H	45 ± 3	108 ± 4	2.4
**28**	NH(CH_2_)_6_CO_2_H	44 ± 2	112 ± 0.3	2.5
**29**	NHCH_2_CH_2_O(CH_2_)_2_CO_2_H	87 ± 0.9	191 ± 0.8	2.2
**30**	NH(CH_2_)_5_CO_2_H	16 ± 1	65 ± 0.9	4.2

a Unless noted otherwise, all amino acid side chains are in the l-configuration. Compound binding affinity for BRDT-BD1 and BRD4-BD1 using our FP assay, where the experiment was performed three times.

b Error-values ± SEM.

To explore bulkier groups, compound **10** (l-Phe) and compound **11** (d-Phe) were synthesized. Both exhibited moderate affinities for BRDT-BD1 (*K_i_* = 28 nM and 56 nM, respectively) and BRD4-BD1 (*K_i_* = 63 nM and 105 nM, respectively), with selectivity ratios of 2.2 and 1.9, respectively. The aromatic side chains of l-Phe and d-Phe appear to enhance selectivity, likely through hydrophobic interactions, with up to 2-fold greater binding affinity for the L-amino acid conformation. Compound **12**, containing a threonine side chain, demonstrated improved affinity for BRDT-BD1 (*K_i_* = 27 nM) and BRD4-BD1 (*K_i_* = 89 nM) as compared to compound **11**. Compound **12** also showed an improved selectivity of 3.3, suggesting that the hydroxyl group of threonine may engage in favorable polar interactions.

Compound **13** with a serine side chain stands out with the highest affinity for BRDT-BD1 (*K_i_* = 8.8 nM), with less affinity for BRD4-BD1 (*K_i_* = 55 nM), and significant selectivity against BRD4-BD1 of 6.3, perhaps once again due to the hydroxyl group in enhancing binding and selectivity within the first bromodomain of BRDT. Similarly, compound **14** with a glutamic acid shows strong affinity for BRDT-BD1 (*K_i_* = 10 nM), less affinity for BRD4-BD1 (*K_i_* = 43 nM), and moderate selectivity (4.2), likely due to the carboxylate group forming ionic interactions in addition to hydrogen bonds. However, when the terminal carboxylic acid is exchanged with an amide, as seen with compound **15** containing a glutamine side chain, nearly equivalent affinities for both targets were observed (*K_i_* = 8.7 nM for BRDT-BD1 and 7.6 nM for BRD4-BD1), with a selectivity ratio of 0.87, demonstrating that the terminal carboxylic acid might be critical for selectivity.

Next, compound **16** with a *tert*-butylamine side chain and compound **17** with a phenethylamine side chain showed weaker BRDT-BD1 affinities (*K_i_* = 94 nM and 46 nM, respectively) and weak BRD4-BD1 affinities (*K_i_* = 101 nM and 58 nM, respectively). Both compounds **16** and **17** exhibited low selectivity (1.1 and 1.3, respectively), suggesting that bulky hydrophobic groups may hinder optimal binding, or increasing the side length might render it less optimal for selectivity.

Compound **18**, containing a methylated Gly at the terminus, showed an improved activity for BRDT-BD1 (*K_i_* = 26 nM) when compared to compound **9** with a glycine side chain (*K_i_* = 41 nM), which indicated that the α-carboxylic acid of compound **9** is not a key interaction for BRDT-BD1. Compound **18** did retain similar BRD4-BD1 binding affinity (*K_i_* = 42 nM) when compared to compound **9** (*K_i_* = 44 nM). Due to compound **18** having improved binding affinity for BRDT-BD1, the selectivity ratio was slightly improved (1.6).

Compound **19** contained two methylene groups and a carboxylic acid within the side chain compared to the side chain in compound **18**. However, despite these modifications, the longer chain length and potentially stronger hydrogen-bond interactions did not yield significantly improved affinities (*K_i_* = 27 nM for BRDT-BD1 and *K_i_* = 49 nM for BRD4-BD1) or selectivity (1.8).

Compound **20** contains three methylene groups within its linker. Once again, increasing the aliphatic acid chain length proved ineffective in improving binding affinity and selectivity ratio. Compound **20** demonstrated weak binding affinities (*K_i_* = 41 nM for BRDT-BD1 and *K_i_* = 47 nM for BRD4-BD1). In addition, although compound **20** contains a γ-carboxylic acid-like compound **14**, which might contribute to hydrogen bonding with Arg54, the selectivity ratio was much lower (1.1) than that of compound **14** (4.2).

We continued investigating linker length and types that connect the terminal carboxylic acid. Compounds **27**, **28**, and **30** contain five to seven methylene groups in the linker. As the linker length increases from five methylene carbons in compound **30** to seven methylene carbons in compound **27**, the binding affinity for BRDT-BD1 becomes weaker (*K_i_* = 16 nM vs 45 nM, respectively). The binding affinity for BRD4-BD1 appears to be the best for five methylene groups, as observed for compound **30** (*K_i_* = 65 nM). The selectivity for BRDT-BD1 is best with the shortest methylene linker in compound **30** (4.2 selectivity). When the linker was changed to a PEG linker, no significant improvement in selectivity was observed. Compound **29** has the same number of atoms as part of the linker as compound **30**. Due to the presence of an oxygen atom in compound **29**, the binding affinity for both proteins (*K_i_* = 87 nM for BRDT-BD1 and *K_i_* = 191 nM for BRD4-BD1) was much lower than that of compound **30** (*K_i_* = 16 nM for BRDT-BD1 and *K_i_* = 65 nM for BRD4-BD1). Compound **26** also contained a PEG linker with a total of eight atoms and exhibited good selectivity of 3.0 (*K_i_* = 39 nM for BRDT-BD1 and *K_i_* = 117 nM for BRD4-BD1). We hypothesized that, due to the greater flexibility of PEG linkers, the terminal carboxylic acid had more rotational degrees of freedom, preventing the optimal interaction angle with Arg54 and leading to the generally observed lack of improvement in binding affinities and selectivity ratios.

In addition to the terminal carboxylic acid group, we explored other terminal functional groups that might interact strongly with the positively charged guanidinium side chain of arginine, such as salt bridge formations. Compound **21**, containing a sulfonic acid, did not exhibit a significant improvement in binding affinity (*K_i_* = 32 nM for BRDT-BD1 and *K_i_* = 56 nM for BRD4-BD1), with a slightly improved selectivity ratio of 1.8. Next, compound **22**, which has a terminal sulfonamide group, also showed no improvement compared to compound **19**, which has a similar linker length but a terminal carboxylic acid group. Compound **22** exhibited moderate binding affinities (*K_i_* = 22 nM for BRDT-BD1 and *K_i_* = 44 nM for BRD4-BD1).

Compound **23**, which contains a terminal amide, also failed to demonstrate improved selectivity (1.7). The terminal amide yielded a weaker binding affinity (*K_i_* = 54 nM for BRDT-BD1 and *K_i_* = 93 nM for BRD4-BD1) compared to the other terminal functional groups seen in compounds **19–22**.

We also wanted to explore non-natural amino acid side chains. To identify the compound with the highest selectivity ratio, we explored compounds **24** and **25**, which feature homoserine and β-homoserine side chains, structurally related amino acids that differ in side-chain length and the position of the hydroxyl group. Compound **24** with the −OH group on the γ-carbon demonstrated equipotent activity for BRDT-BD1 (*K_i_* = 8.3 nM) when compared to compound **13** (*K_i_* = 8.8 nM). However, compound **24** exhibited a slightly stronger binding affinity for BRD4-BD1 (*K_i_* = 32 nM) compared to compound **13**, resulting in a lower selectivity ratio of 3.8. Compound **25** with the −OH group on the β-carbon demonstrated weaker binding affinities for both proteins (*K_i_* = 21 nM for BRDT-BD1 and *K_i_* = 41 nM for BRD4-BD1) compared to compound **13**. It exhibited a lower selectivity ratio of 2.0.

Similar selectivity values for BRDT-BD1 over BRD4-BD1 were obtained for compounds **8**–**23** in an AlphaScreen assay (SI, Table S1). In both the FP and AlphaScreen assays, compounds **13** and **14** provided the highest selectivity of the compounds tested. Building on the promising selectivity profile observed in these biochemical assays, we selected compounds **13** and **14** for further biophysical characterization based on their superior potency and selectivity for BRDT-BD1 over other bromodomains.

While Table 1 reports biochemical affinity for isolated BD1 domains, these measurements do not report BD1 versus BD2 engagement within the physiologically relevant tandem construct. To validate the preferred binding of compounds **13** (Figure 5) and **14** (Figure 6) to the first bromodomain, we employed ^19^F-NMR with both BRDT and BRD4 tandem domains (BRDT-T and BRD4-T),^52^ extending our earlier fluorescence polarization studies on isolated bromodomains. To monitor ligand binding qualitatively, we employed 5-fluorotryptophan (5FW) labeling of tryptophan residues in both bromodomains of BRDT and BRD4, facilitating protein-observed fluorine NMR (PrOF NMR) detection of small-molecule interactions.^53,54^ In the BRDT tandem domain (BRDT-T), Trp50 (W50) is located within the WPF shelf proximal to the acetyl-lysine (Kac) binding pocket of BRDT’s first bromodomain (BD1), while Trp293 (W293) occupies the corresponding WPF shelf in the second bromodomain (BD2). These strategically positioned tryptophan residues serve as sensitive reporters of ligand-binding interactions via ^19^F NMR. Similarly, in the BRD4 tandem domain (BRD4-T), Trp81 (W81) is placed within the WPF shelf adjacent to the acetyl-lysine (Kac) binding site of the first bromodomain (BD1). In contrast, Trp374 (W374) occupies the analogous WPF shelf in the second bromodomain (BD2).

**Figure 5:**
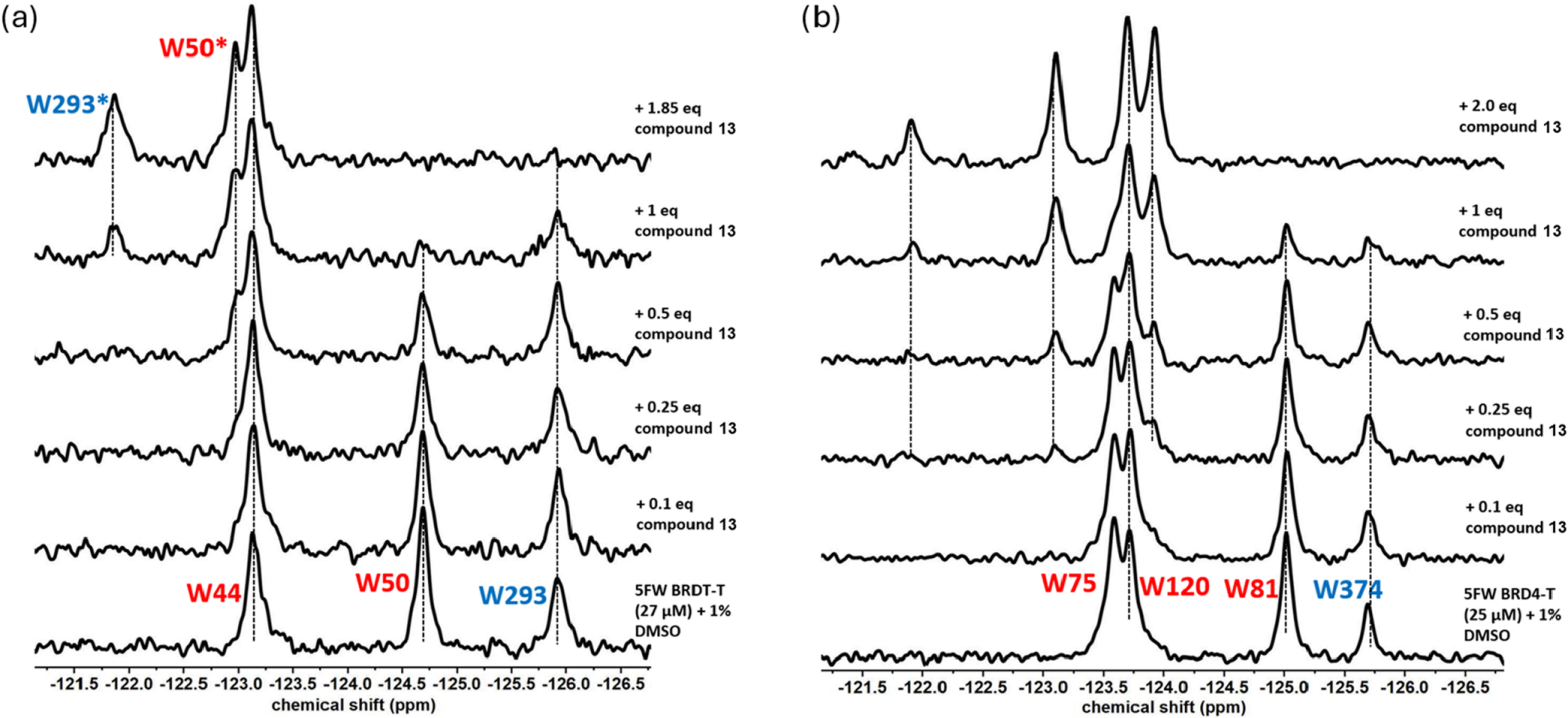
PrOF NMR experiments of compound **13** with 5FW BRDT-T and 5FW BRD4-T. (a) Stacked ^19^F NMR spectra with an increasing concentration of compound **13** with 27 μM 5FW BRDT-T. W50 and W293 are the WPF shelf tryptophans in BD1 and BD2 of BRDT-T, colored red and blue, respectively. (b) Stacked ^19^F NMR spectra with an increasing concentration of compound **13** with 25 μM 5FW BRD4-T. W81 and W374 are the WPF shelf tryptophans in BD1 and BD2 of BRD4-T, colored red and blue, respectively.

**Figure 6:**
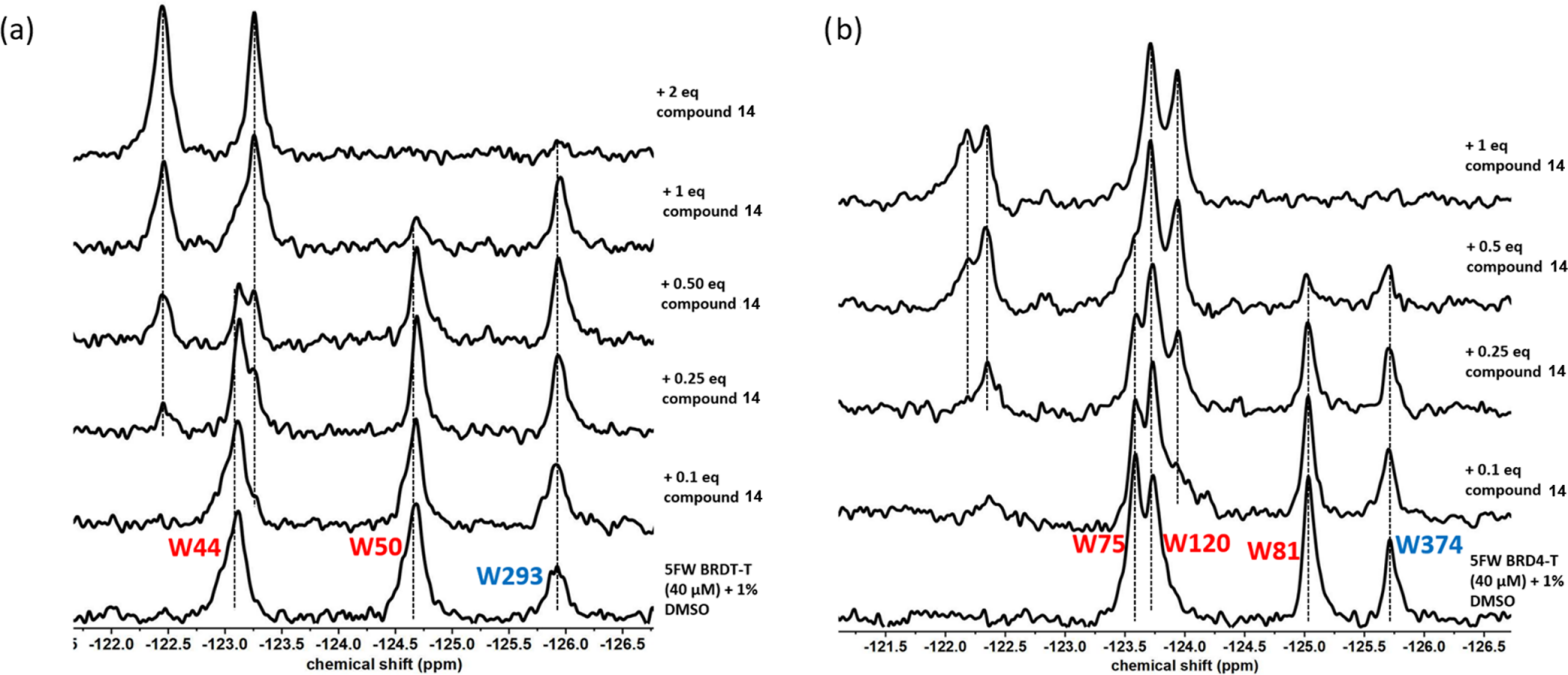
PrOF NMR experiments of compound **14** with 5FW BRDT-T and 5FW BRD4-T. (a) Stacked ^19^F NMR spectra with an increasing concentration of compound **14** with 27 μM 5FW BRDT-T. W50 and W293 are the WPF shelf tryptophans in BD1 and BD2 of BRDT-T, colored red and blue, respectively. (b) Stacked ^19^F NMR spectra with an increasing concentration of compound **14** with 25 μM 5FW BRD4-T. W81 and W374 are the WPF shelf tryptophans in BD1 and BD2 of BRD4-T, colored red and blue, respectively.

Titration of compound **13**, dose-dependently based on the molar ratio of BRDT-T (Figure 5a), affected the resonance of W50 in BD1, broadening it to the baseline, consistent with a high-affinity interaction. After saturating the binding site of BD1 at stoichiometric concentration, the BD2 resonance broadens as indicated by the W293 resonance peak. When compound **13** was titrated similarly with BRD4-T (Figure 5b), BD1 selectivity can be observed by the more intense drop of the W81 resonance peak with increasing equivalency of the compound; however, the difference in the rate of the intensity drops is small relative to the perturbations observed for BRDT-T.

The titration of compound **14** was done in the same manner. Similar BD1 selectivity was observed based on titration experiments with BRDT-T and BRD4-T. Upon titrating compound **14** with BRDT-T (Figure 6a), the resonance of W50 of BD1 broadened into the baseline. Further titration led to the broadening of W293 of BD2. With BRD4-T (Figure 6b), a drop in the intensity of W81 of BD1 is observed, as well as a drop in the intensity of W374 of BD2, but at a lower intensity. In summary, our PrOF NMR studies suggest that compounds **13** and **14** are both BD1-preferring, with compound 14 showing a similar but higher preference.

To fully characterize the selectivity profile of compound **13**, with the higher selectivity for BRDT-BD1 observed in the FP assay, we assessed its binding affinity against both individual (BD1 and BD2) and tandem bromodomains of BRDT and BRD4 using Eurofins BROMOscan, a high-throughput selectivity screening platform (Table 2). The results revealed a 14-fold selectivity for BRDT-BD1 over BRD4-BD1, exceeding the selectivity ratio observed in our fluorescence polarization (FP) assays (6-fold), but similar to the AlphaScreen assay selectivity (13-fold, SI, Table S1). The true potency of highly potent inhibitors is likely underestimated in the FP assay due to a floor effect, due to the bromodomain protein concentration needed (75 nM),^55^ which would explain the lower selectivity ratio. The divergence between FP and BROMOscan results may reflect differences in assay conditions such as ligand occupancy, protein constructs, or detection methods, highlighting the importance of orthogonal validation in selectivity profiling. Interestingly, this pronounced selectivity was specific to BRDT-BD1 and was not recapitulated in BRD4, since compound **13** instead preferred BRD4-BD2 over BRD4-BD1 in the BROMOscan assay. This finding reinforces the conclusion that compound **13** is a BRDT-BD1-preferring inhibitor. Interestingly, the affinity of compound **13** for the BD2 domains was similar to that of the tandem domains for both BRDT and BRD4, suggesting that binding to BD2 drives overall affinity for the tandem domains in the BROMOscan platform. However, this observation may reflect limitations of competitive binding assays for proteins with multiple ligand-binding sites, since selectivity for BD1 was clearly observed in PrOF NMR studies using BRDT-T and BRD4-T.

**Table 2:** *K*_D_ Determination for Compound **13** Tested by BROMOscan (Eurofins)

	*K*_D_ (nM)
BRDT-BD1	1.6
BRDT-BD2	6.1
BRDT-Tandem	5.0
BRD4-BD1	22
BRD4-BD2	2.9
BRD4-Tandem	2.7
BRD3-BD1	49
BRD3-BD2	15
BRD2-BD1	110
BRD2-BD2	23

We further assessed the selectivity across the broader BET family (Table 2). Compound **13** showed substantially weaker binding to the BRD2 and BRD3 bromodomains than to BRDT. For BRD2-BD1, the compound exhibited a *K*_D_ of 110 nM, corresponding to a 69-fold preference for BRDT-BD1 (*K*_D_ = 1.6 nM). Similarly, its *K*_D_ of 49 nM for BRD3-BD1 reflects a 31-fold selectivity for BRDT-BD1. These results further highlight compound **13’s preference** for BD1 of BRDT. Regarding the BD2 domain, compound **13** also showed a modest preference for BRDT-BD2, with affinities only 2.5- to 3.8-fold higher than those for BRD3-BD2 and BRD2-BD2, respectively. However, BROMOscan profiling indicates that compound **13** binds BRDT-BD1 with high affinity but also retains substantial affinity for BRDT-BD2 and BRD4-BD2, suggesting that full BRDT-BD1 selectivity across all BET bromodomains was not achieved. Accordingly, we describe compound **13** as BRDT-BD1-preferring rather than BRDT-BD1-selective. To further evaluate selectivity for the tandem bromodomains, we used the AlphaScreen assay (Reaction Biology Corp.). Compound **13** demonstrated double-digit nanomolar potency against BRDT-T (56 nM) while demonstrating limited affinity for BRD4-T (2.0 μM), indicating a 35-fold selectivity for BRDT over BRD4 (Figure 7). The lower potency of compound **13** in the AlphaScreen assay relative to the BROMOscan, particularly for BRD4-T, may reflect the high avidity of the bromodomain-substrate interaction in the bead-based assay, which reduces the potency of competitive inhibitors.

**Figure 7:**
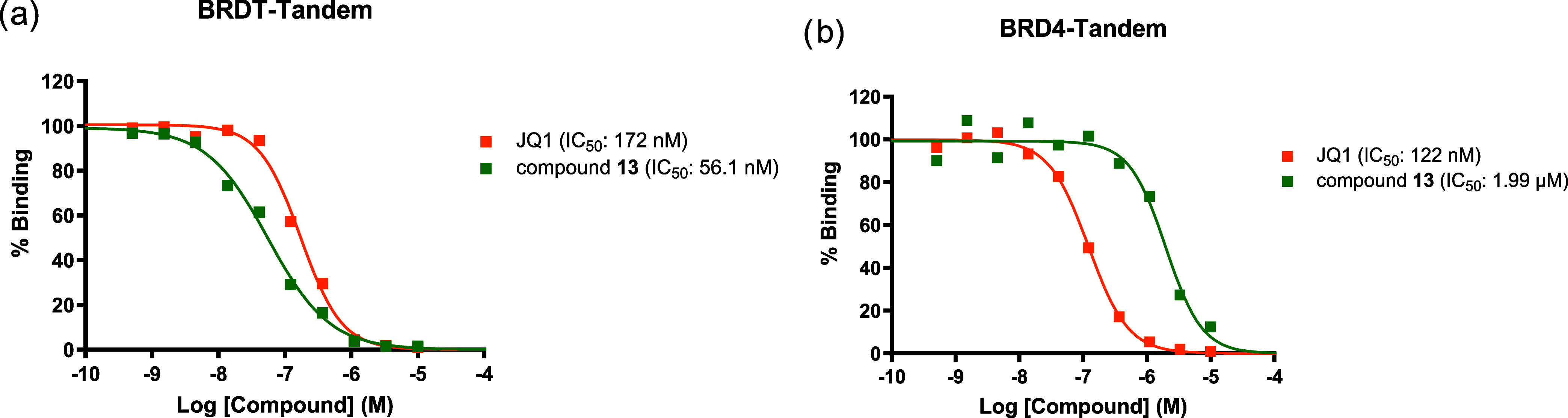
AlphaScreen experiments conducted by Reaction Biology Corporation. (a) Dose–response curve showing the binding percentage of BRDT-Tandem by compound **13** using reference compound **JQ1**. (b) Dose–response curve showing the binding percentage of BRD4-Tandem by compound **13** using reference compound **JQ1**.

It is important to note that apparent affinity and selectivity values vary across assay platforms due to differences in construct design and detection methods. The apparent IC_50_ values from AlphaScreen are not expected to match equilibrium *K*_D_ values from BROMOscan on a numerical basis, and the two assays are best viewed as complementary rather than directly comparable. BROMOscan is a competitive binding assay performed with isolated bromodomains, whereas AlphaScreen employs bead-based proximity detection and, in the case of tandem constructs, may be influenced by avidity effects arising from two binding sites within the same protein. Consequently, apparent *K*_D_ values for tandem domains can reflect the highest-affinity site rather than strict domain preference. In contrast, PrOF NMR provides a protein-observed readout that directly monitors perturbations of BD1- and BD2-resident tryptophan residues within the tandem construct. The preferential broadening of BD1 resonances for compound **13** supports BD1-preferring engagement despite differences in absolute affinity values observed across biochemical platforms. Together, these orthogonal assays provide a comprehensive assessment of binding behavior.

Simultaneously targeting both kinases and BET family proteins does not always yield therapeutic benefits. For instance, JAK2 inhibitors are commonly associated with adverse hematopoietic effects, including disruptions in blood cell differentiation, anemia, thrombocytopenia, and other hematologic complications.^56^ These side effects highlight the need for careful evaluation of dual-inhibitor strategies to balance therapeutic efficacy with potential toxicity. Hence, we tested compounds **SG3–179, 13**, and **14** for their inhibitory activity against JAK2 and FLT3 (Table 3) using an AlphaScreen assay (Reaction Biology Corp.), as the parent compound **SG3–179** is a known JAK2 and FLT3 inhibitor. Compounds **13** and **14** were significantly less potent JAK2 and FLT3 inhibitors (>300-fold) than **SG3–179**. We also evaluated **SG3–179** and compounds **13** and **14** in an AlphaScreen assay against BRDT and BRD4, including both BD1 and BD2, and observed a preference for BRDT-BD1 over BRD4-BD1 for compounds **13** and **14**, as well as reduced activity against the BD2 domains (SI, Table S2). While prior reports indicate that **SG3–179** displays broadly potent BET bromodomain activity, a full head-to-head BD1/BD2 profiling of **SG3–179** across BRDT/BRD4 and BRD2/BRD3 was not generated in the current study. Therefore, we focused our expanded domain profiling on the most informative lead analogs.

**Table 3:** JAK2, and FLT3 Activity^a^

	IC_50_ (nM)	
compound	JAK2	FLT3
**SG3–179**	12	32
**13**	8130	>10,000
**14**	>10,000	>10,000

a AlphaScreen performed by Reaction Biology Corporation.

As previously observed, compound **13** again showed a preference for BRDT-BD1. In this case, a 14-fold preference for BRDT-BD1 was observed (BRDT-BD1 IC_50_: 19 nM vs BRD4-BD1 IC_50_: 256 nM). In addition, compound **13** exhibited a 428-fold selectivity against JAK2 and a greater than 526-fold selectivity against FLT3. For compound **14**, a 6-fold selectivity for BRDT-BD1 against BRD4-BD1 was observed. Similarly, compound **14** exhibited selectivity greater than 476-fold against JAK2 and FLT3, indicating that the structural modification led to a significant loss of kinase activity relative to the parent compound.

We next assessed the antiproliferative activity of compounds **13** and **14** in the MM.1S multiple myeloma cell line via CellTiter-Glo viability assays (Figure 8). The MM.1S cell line was chosen for its sensitivity to BET inhibition and its dependence on BRD4- and BRDT-regulated oncogenic transcription factors, particularly c-Myc, which is essential for myeloma cell survival and growth. This experiment was intended to assess cellular activity consistent with inhibiting the BET pathway rather than to assign BRDT-specific cellular mechanisms. In comparative testing, compounds **13** and **14** exhibited IC_50_ values of 4.3 μM and 6.8 μM, respectively, whereas the reference inhibitors JQ1 and I-BET151 were more potent (IC_50_ = 0.12 μM and 0.33 μM, respectively). Because BRDT is testis-restricted, the observed effects in MM.1S cells are most plausibly explained by engagement of somatic BET proteins such as BRD4 rather than BRDT-specific inhibition. Consistent with the mechanisms of BET inhibitors, Western blot analysis revealed a dose-dependent suppression of c-Myc protein levels following compound treatment (Figure 9).

**Figure 8:**
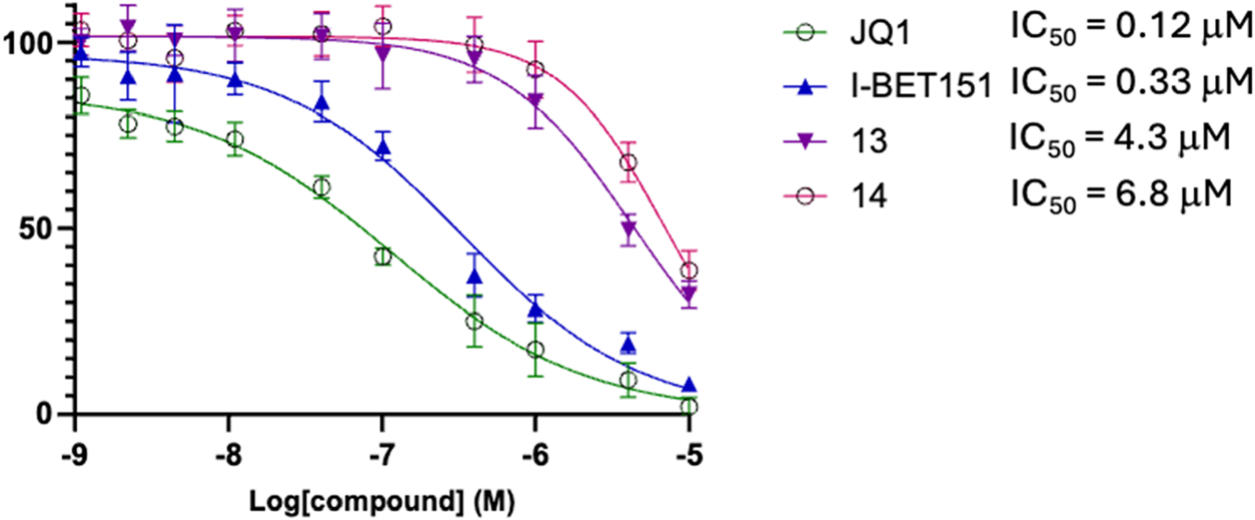
Inhibition of MM.1S cancer cell growth by compounds **13** and **14** with reference compounds **JQ1** and **I-BET151** using the CellTiter-Glo assay.

**Figure 9:**
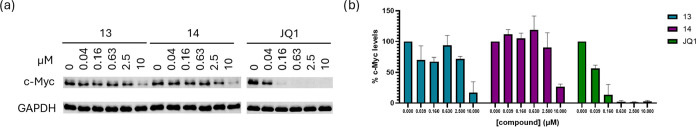
Inhibition of c-Myc expression by compound **13**, **14**, and **JQ1**. (a) Western blot detection of c-Myc levels in MM.1S cells after a 6 h drug exposure. (b) Quantification of the experiment from (a).

We attempted to cocrystallize both of our lead compounds. However, only compound **14** featuring a glutamic acid side chain could be cocrystallized with BRDT-BD1 and BRD4-BD1 (Figure 10). Compound **14** exhibited similar binding poses in both bromodomains, with its *tert*-butyl group occupying the WPF shelf, the Glu side chain extending into the ZA channel, and the piperidine moiety solvent-exposed. Observed key interactions included hydrogen bonds between the pyrimidine core of compound **14** and Asn140 in BRD4-BD1 (Figure 10b) or Asn109 in BRDT-BD1 (Figure 10c), as well as an additional hydrogen bond to Pro82 in BRD4-BD1 (Figure 10b) or Pro51 in BRDT-BD1 (Figure 10c).

**Figure 10:**
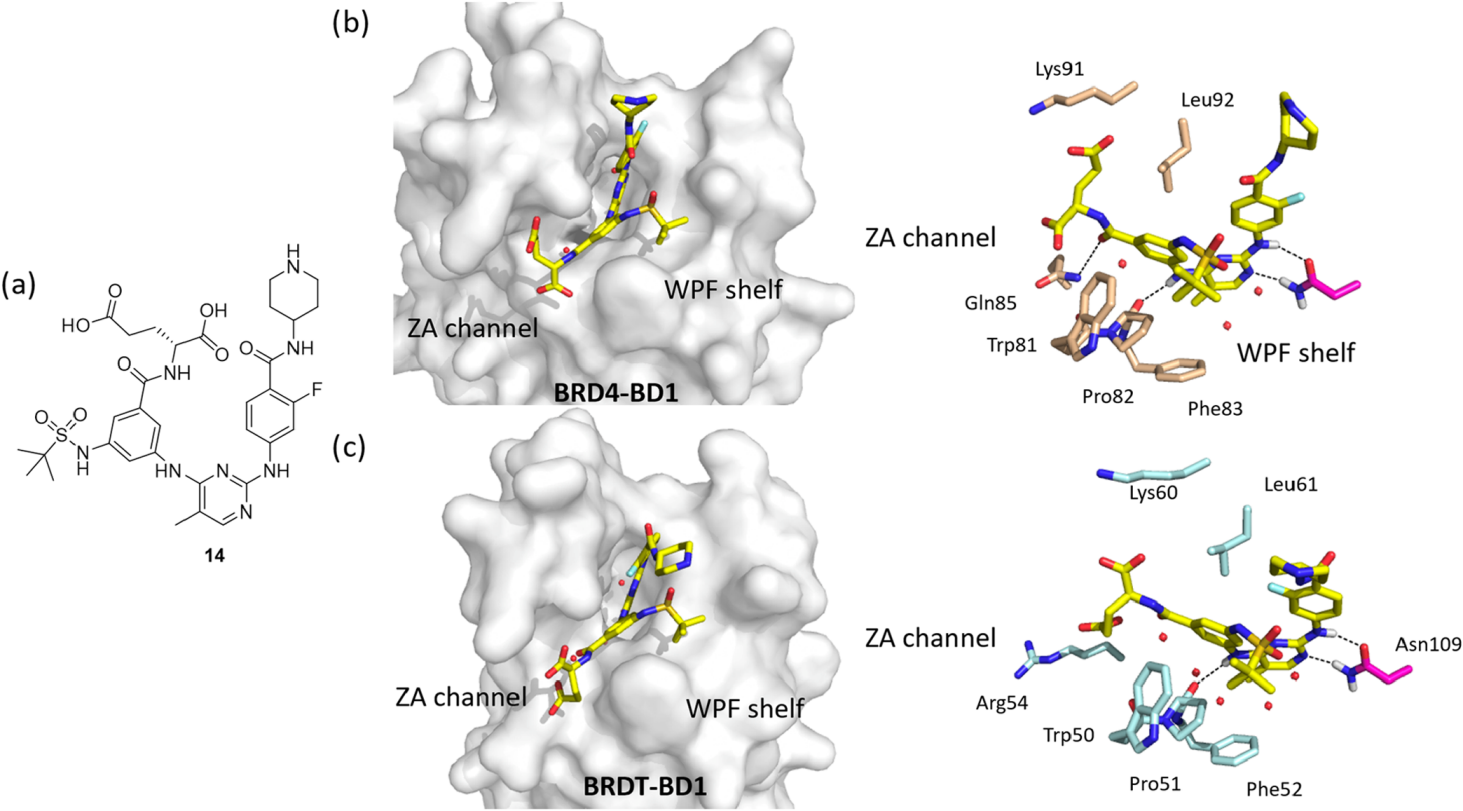
(a) Structure of compound **14**. (b) Co-crystal structure of compound **14** with BRD4-BD1 (PDB 7MR8). The inhibitor is shown in yellow, and the neighboring residues are shown in beige. H-bonds are indicated as black dotted lines. (c) Co-crystal structure of compound **14** with BRDT-BD1 (PDB 9YCQ). The inhibitor is shown in yellow, and the neighboring residues are shown in light blue. H-bonds are indicated as black dotted lines. The electron density maps are shown in Supporting Figure S2.

Surprisingly, the BRDT-BD1 cocrystal structure showed no direct interaction between the inhibitor’s acid groups and Arg54 (Figure 10c). Notably, the ZA channel in BRDT-BD1 contained five water molecules (Figure 10c) as previously known (Figures 1 and 5), compared to only three in BRD4-BD1 (Figure 10b), which differs from the presence of five conserved water molecules in its unbound state (Figure 3). Since water displacement is a known strategy for improving ligand selectivity and binding entropy,^57–60^ the reduced hydration resulting from the displacement of two conserved water molecules near the ZA channel in BRD4-BD1 likely decreased the binding affinity of compound **14**. Furthermore, this disruption likely destabilized BRD4-BD1’s binding loop, correlating with the compound’s selectivity for BRDT-BD1, while the preserved water network in BRDT-BD1 may stabilize the ZA channel. The ligand’s slight positional shift in BRDT-BD1’s Kac site suggests these waters contribute to differential binding potentials.

We conducted several in vitro assays to assess our lead compound’s properties for further development. First, we performed comprehensive metabolic stability studies on compound **13**, which features a serine-derived side chain, using liver microsomes from both human and mouse models (Table 4). In human liver microsomes, compound **13** exhibited outstanding metabolic resistance, as evidenced by a prolonged half-life (*T*_1/2_) of 231 min. This remarkably slow degradation rate was accompanied by an ultralow intrinsic clearance CL_int_ of 5.99 μL/min/mg protein. When scaled to physiological conditions, hepatic clearance was calculated to be 7.52 mL/min/kg, indicating minimal first-pass metabolism. These parameters suggest that the compound would likely maintain stable plasma concentrations in humans, with a reduced risk of rapid metabolic inactivation. Metabolic stability was further confirmed in mouse liver microsomes, where compound **13** exhibited similar favorable degradation kinetics. This cross-species consistency enhances the translational potential of our findings and supports the compound’s suitability as a lead for further development. The MDCK-MDR1 permeability assay (Tables S4 and S5) indicates low cellular uptake (<0.22 × 10^–6^ cm/s), as reflected by the lower IC_50_ value (4.3 μM) observed in the antiproliferative assay in MM.1S cell lines compared with JQ1, the positive control compound. Because MDCK-MDR1 cells overexpress P-gp, the low apparent permeability may reflect active efflux and transporter liability in addition to limited passive permeability. Given the high polarity/ionization expected for compound **13**, these results suggest that the cellular activity is likely constrained by permeability and/or efflux rather than intrinsic target potency. It should also be noted that apparent microsomal stability can be influenced by nonspecific binding to microsomes, *f*_u,mic_.^61^ Although *f*_u,mic_ was not determined here, the reported stability values provide a comparative assessment under identical assay conditions.

**Table 4:** Metabolic Stability of Test Compounds in Pooled Human and Male Mouse Liver Microsomes and Permeability in the MDCK-MDR1 Cell Line

compound	species	*T*_1/2_ (min)	CL_int_ (μL/min/mg protein)	Scaled-up CL_int_ (mL/min/kg)	MDCK-MDR1 (10^–6^ cm/s)^a^
Diclofenac	Human	9.4	147	185	ND^b^
	Mouse	47	30	130	
compound **13**	Human	231	6.0	7.5	<0.22
	Mouse	870	1.6	7.0	

a The permeability for compound **13** was evaluated using the MDCK cell line in the presence of 2% BSA. Permeability was measured by *P*_app_ (A-B). Further assay details are provided in Supporting Tables 5 and 6.

b ND = not determined.

## Conclusion

Through a structure-guided derivatization campaign of the dual BRD4-kinase inhibitor **TG101209**, we developed two novel BRDT-BD1-preferring compounds, **13** and **14**. Selectivity for BD1 over BD2 in BRDT was demonstrated in FP, AlphaScreen, and PRoF NMR assays. Compound **13** exhibits 14-fold selectivity for BRDT-BD1 over BRD4-BD1 (BROMOscan) and 35-fold selectivity for the BRDT-tandem bromodomain over the BRD4-tandem bromodomain, as determined by AlphaScreen. Leveraging cocrystal structures of the **TG101209**-related probe **SG3–179**, we optimized binding interactions to achieve domain specificity, which crystallographic studies revealed to be mediated by displacement of conserved water networks in the BRDT-BD1 pocket. Both compounds demonstrate functional efficacy in MM.1S multiple myeloma cells, suppressing proliferation and downregulating oncogenic c-Myc expression. Notably, compound **13** displayed exceptional metabolic stability, suggesting promising pharmacokinetic properties of this lead compound. However, compound **13** exhibited poor permeability, indicating the need for further modifications to improve its property. While compound **13** exhibits strong selectivity for BRDT-BD1 relative to other BET BD1 domains, cross-platform profiling revealed high affinity for BRD4-BD2 in isolated-domain assays, underscoring the importance of considering assay format and construct context when interpreting domain selectivity. Nevertheless, these findings yielded a chemical probe for BRDT 35-fold selective over BRD4. Furthermore, based on the crystallographic findings, we conclude that the observed preference for BRDT-BD1 over BRD4-BD1 is more likely driven by differences in local hydration and pocket microenvironment than by a single-residue interaction with Arg54, thereby establishing a framework for targeting water-displacement strategies in the design of selective BRDT-BD1 inhibitors.

## Experimental Section

### Protein Expression

BRDT-1, BRD4–1, fluorinated BRDT-T, and BRD4-T were expressed and purified as previously described.^46,53,54^

### Fluorescence Polarization Assay

The affinities of BRDT-1 and BRD4–1 inhibitors were determined in eight-point dose–response experiments, performed in triplicate, with (+)-JQ1 (Sigma-Aldrich) as a positive control using a previously described method.^55^ Dose–response curves for all compounds may be found in the SI, Figure S1. The compounds were dissolved and diluted to 10 mM in DMSO and then added to appropriate source plate wells (Beckman Coulter) and diluted to the appropriate intermediate dilution concentrations using the Echo 550 contactless liquid handler (Labcyte) to achieve the desired concentration ranges for each compound with 0.1% final DMSO concentration in 384-well destination plates (Corning 4511). BET proteins prepared in assay buffer, comprising 150 mM NaCl, 3 mM DTT, 4 mM CHAPS, 50 mM sodium phosphate, and pH 7.4, were then added to the appropriate wells of the destination plate to achieve a final concentration of 75 nM. Finally, our in-house probe, compound **16** from our previous report,^55^ was diluted into the assay buffer and added to each well to reach a final concentration of 7.5 nM. The plates were shaken using a Mircomix5 (DPC) and centrifuged on a Centrifuge 5804R (Eppendorf) for 1 min. The plates were sealed and incubated at room temperature for 1 h. Then, the FP values were measured on a CLARIOstar (BMG Labtech) plate reader (482–16 nm excitation, 530–40 nm emission, 504 nm dichroic). Each experiment was performed >3 times. IC_50_ values were calculated by fitting concentration–response data to the log­(inhibitor) vs response equation in GraphPad Prism (version 10.3.1), with 0 and 100% control values defined by the total change of polarization. Specifically, 0% value is defined by the value obtained in the presence of the probe at 7.5 nM, while a 100% polarization reading is defined by 75 nM of the BET protein of interest in complex with the probe at a concentration of 7.5 nM. For compounds with incomplete concentration–response curves, the IC_50_ values were determined by fixing the bottom to 0. High concentrations that did not conform to a standard sigmoidal curve, likely due to fluorescence interference and/or lack of solubility, were excluded. *K_i_* values were calculated using the equation of Nikolovska-Coleska et al. Reported *K_i_* values represent the mean of at least three independent experiments, and errors are expressed as the standard error of the mean (SEM), reflecting the precision of the estimated mean *K_i_*.^62^

### Protein-Observed Fluorine NMR

The NMR study was carried out as previously described.^53^ Recombinant expression of fluorinated BET bromodomains has been reported previously.^53^ Experiments were run on a Bruker 600-MHz Avance NEO (6002), equipped with a 5 mm triple-resonance cryoprobe. 5FW-labeled bromodomains were diluted in 50 mM HEPES, 100 mM NaCl, pH 7.4 buffer, by adding D2O and 0.1% TFA/H2O to final concentrations of 4% and 0.04%, respectively. Two one-dimensional ^19^F NMR spectra were taken of the control protein sample at an O1P of −75 ppm, NS = 16, D1 = 1, AQ = 0.5 s (TFA Reference set to −75.25 ppm), and an O1P of −125 ppm, NS = 1000–3000, D1 = 0.6, AQ = 0.05 s (protein resonances). Ligand stock solutions prepared in d6-DMSO were titrated into bromodomain protein solutions (40 μM) to yield a constant DMSO concentration of 1% v/v. All titrations were performed as single replicates.

### Protein Crystallization and Crystallography

Crystals of BRDT-1 and BRD4–1 were grown in the presence of 1 mM compound **14** by vapor-diffusion in hanging droplets using 0.2 M ammonium sulfate, 0.1 M TRIS pH 8.5, 25% (w/v) poly­(ethylene glycol) 3350 at 19 °C. Crystals were harvested in cryoprotectant (precipitant +25% (v/v) ethylene glycol) and flash-frozen in liquid nitrogen. X-ray diffraction data were recorded at beamlines 22-BM and 22-ID (SER-CAT) of the Advanced Photon Source at Argonne National Laboratory. Data were reduced and scaled with XDS.^63^ PHENIX^64^ was employed for phasing and refinement, and model building was performed using Coot.^65^ The structures were solved by molecular replacement using PDB entry 4O70 as the search model. An initial model of the inhibitor was generated with ligand restraints from the elbow of the PHENIX suite. All structures were validated by MolProbity^66^ and phenix.model_vs_data.^67^ Data collection and refinement statistics are shown in Supporting Table S3.

### Cell Viability and Signaling

#### CellTiter-Glo Assay

MM.1S cells (CRL-2974) were cultured and maintained in RPMI-1640 medium supplemented with 10% fetal bovine serum and 1% 100 μg/mL penicillin-streptomycin solution. Compounds of interest and positive control compounds were dissolved to 10 mM in DMSO and then added to appropriate source plate wells (Beckman Coulter) and diluted to appropriate intermediate dilution concentrations using the Echo 550 contactless liquid handler (Labcyte) to achieve the desired 10-point concentration ranges for each compound with 0.1% final DMSO concentration in 384-well destination plates (Greiner 781974). Twenty-five μL of culture medium containing 5000 cells was seeded into each well with the test compounds, and the plate was incubated for 72 h at 37 °C and 5% CO_2_. After 3 days, 25 μL of CellTiter-Glo 2.0 reagent (Promega) was added to each well, followed by orbital shaking for 1 min. Luminescence signals were recorded by Envision 2103 Multilabel Reader (PerkinElmer) using excitation and emission filters of 570 and 615 nm, respectively. IC_50_ curves and values were calculated using the GraphPad Prism program based on a sigmoidal dose–response equation. Each experiment was performed >3 times.

#### Western Blot

Western blot experiments were carried out as previously described.^46,53^ Western blot experiments with MM.1S cells (CRL-2974) were performed by coincubating with the compound of interest at a density of 1 × 10^6^ cells/well in a 6-well cell culture plate (Corning 3506) for 6 h. MM1. S cells were then lysed in M-PER Mammalian Protein Extraction Reagent (Thermo Fisher Scientific) supplemented with Halt Protease Inhibitor Cocktail (Thermo Fisher Scientific). Protein concentrations were determined using the Pierce BCA Protein Assay (Thermo Fisher Scientific). Equal amounts of protein of 20 μg per lane were mixed with 2× Laemmli buffer (Bio-Rad) and heated at 95 °C for 5 min, then loaded onto 4–20% Mini-PROTEAN TGX Precast Protein Gels (Bio-Rad). Proteins were separated by gel electrophoresis and transferred onto PVDF membranes (Bio-Rad) using a wet transfer system (Bio-Rad) at 100 V for 1 h at 4 °C. Membranes were washed with TBST buffer (20 mM Tris, 150 mM NaCl, 0.05% Tween), then blocked with EveryBlot Blocking buffer (Bio-Rad) for 5 min at room temperature. Primary antibodies were diluted in blocking buffer and incubated for 90 min at room temperature: GAPDH (1:5000, Cell Signaling, #2118) and c-Myc (1:250, Cell Signaling, #5605). After five 5 min TBST washes while shaking, membranes were incubated with horseradish peroxidase (HRP)-conjugated antirabbit secondary antibody (1:1000, Cell Signaling, #7074) for 1 h at room temperature. Protein bands were visualized using enhanced chemiluminescence (ECL) substrate (Pierce) and imaged with a ChemiDoc XRS+ system (Bio-Rad). Densitometric analysis was performed using ImageLab software (Bio-Rad, version 6.1).

#### Synthesis

##### General

All commercial chemicals and solvents were reagent grade and were purchased from Sigma-Aldrich, Oakwood, TCI Chemicals, Alfa Aesar, Fisher Scientific, or Enamine. Commercially available starting materials and reagents were used without further purification unless otherwise specified. All reactions that required anhydrous conditions were run under a nitrogen atmosphere. Reactions were monitored by thin layer chromatography (TLC) performed on silica gel 60 F_254_ plates (Sigma-Aldrich 1.05715) and visualized under 254 nm UV light or monitored via single quadrupole LC-MS (Agilent 1260 Infinity series II/LC/MSD iQ) fitted with a C18 10 × 250–5 μm column, on a mobile phase of ACN/H_2_O + 0.1% formic acid. Purifications were conducted using medium-pressure liquid chromatography (MPLC) on a CombiFlash Companion (Teledyne ISCO) with prepacked silica columns (20–40 μm) and UV detection at 254 nm. ^1^H and ^13^C NMR spectra were obtained on a Bruker 400/100 MHz Avance DPX. Chemical shifts are reported in parts per million (ppm, δ units). Splitting patterns are designed as s, singlet; d, doublet; t, triplet; q, quartet; m, multiplet; brs, broad singlet. All NMR data were processed using MestReNova. All tested compounds are >95% pure by HPLC analysis. For LC-MS purity testing, compounds were dissolved in DMSO to make 10 mM stock solutions. The stock solutions were then diluted 1000-fold using MeOH and filtered (0.2 μm, PTFE) before purity analysis using an Acquity UPLC (Waters Corporation) equipped with an Acquity BEH UPLC C18, 1.7 μm (2.1 × 50 mm) column for separation. Light was detected at 214 nm using a photodiode array detector. Mass data were acquired using a Micromass ZQ mass spectrometer. LC was completed using a gradient method with each of the following mobile phase systems: Mobile Phase A2:95% water, 5% acetonitrile, 0.1% formic acid; Mobile Phase B2:95% acetonitrile, 5% water, 0.1% formic acid; Mobile Phase A1:95% water, 5% MeOH, 0.1% formic acid; Mobile Phase B1:95% MeOH, 5% water, 0.1% formic acid.

##### Methyl 3,5-Diaminobenzoate (**1**)

To a mixture of 3,5-diaminobenzoic acid (1 equiv, 4.0 g, 26 mmol) and MeOH (75 mL), concentrated sulfuric acid (2.5 equiv, 3.5 mL, 66 mmol) was added slowly at 0 °C dropwise. The mixture was stirred at room temperature for 5 h. The mixture was washed with a saturated solution of NaHCO_3_ and extracted with EtOAc. The organic layer was dried over MgSO_4_ and concentrated under reduced pressure to provide the title compound as a white solid (4.0 g, 91%). Mp: 129.6–130.1 °C. ^1^H NMR (400 MHz, acetone-*d*_6_) δ 6.64 (d, *J* = 2.1 Hz, 2H), 6.23 (d, *J* = 2.0 Hz, 1H), 4.56 (brs, 3H), 3.78 (s, 3H).

##### Methyl 3-Amino-5-((2-chloro-5-methylpyrimidin-4-yl)­amino)­benzoate (**2**)

To a solution of methyl 3,5-diaminobenzoate (**1**, 1 equiv, 80 mg, 0.48 mmol) and 2,4-dichloro-5-methylpyrimidine (1 equiv, 78 mg, 0.48 mmol) in *n*-BuOH (1 mL) was added DIPEA (3.9 equiv, 0.33 mL, 1.9 mmol). The mixture was stirred at 120 °C for 24 h, then cooled to room temperature. The solvent was removed under reduced pressure, and the residue was purified by silica gel column chromatography (RediSep Gold silica gel column, 10% MeOH in DCM) to give the title compound as a brown solid (97 mg, 69%). Mp: 130.1–130.7 °C. ^1^H NMR (400 MHz, DMSO-*d*_6_) δ 8.76 (s, 1H), 8.04 (s, 1H), 7.38 (s, 1H), 7.15 (s, 1H), 6.99 (s, 1H), 5.46 (s, 2H), 3.82 (s, 3H), 2.16 (s, 3H).

##### Methyl 3-((*tert*-Butylsulfinyl)­amino)-5-((2-chloro-5-methylpyrimidin-4-yl)­amino)­benzoate (**3**)

To a solution of methyl 3-amino-5-((2-chloro-5-methylpyrimidin-4-yl)­amino)­benzoate (**2**, 1 equiv, 1.3 g, 4.4 mmol) in pyridine (10 mL) was added a solution of *tert-*butylsulfinyl chloride (1 equiv, 0.62 g, 4.4 mmol) in DCM (10 mL) dropwise at 0 °C under nitrogen. The mixture was stirred at 0 °C for 2 h, then warmed to room temperature and stirred for an additional 20 h. The solvent was removed under reduced pressure. The residue was diluted with EtOAc (20 mL) and washed with HCl (1 *M*, 1 × 50 mL) and water (1 × 50 mL). The organic layer was dried with Na_2_SO_4_ and concentrated under reduced pressure. The yellow oil was purified by silica gel column chromatography (RediSep Gold silica gel column, 80–90% EtOAc in hexanes) to give the title compound as a pale-yellow foam (1.6 g, 89%). ^1^H NMR (400 MHz, acetone-*d*_6_) δ 8.34 (s, 1H), 8.11 (s, 1H), 8.05 (s, 1H), 7.94 (s, 1H), 7.50 (s, 1H), 7.31 (s, 1H), 3.89 (s, 3H), 2.31 (s, 3H), 1.35 (d, *J* = 1.2 Hz, 9H).

##### Methyl 3-((2-Chloro-5-methylpyrimidin-4-yl)­amino)-5-((1,1-dimethylethyl)­sulfonamido)­benzoate (**4**)

To a solution of methyl 3-((*tert*butylsulfinyl)­amino)-5-((2-chloro-5-methylpyrimidin-4-yl)­amino)­benzoate (**3**, 1 equiv, 10 mg, 25 μmol) in DCM (1 mL) was added mCPBA (1 equiv, 4.4 mg, 25 μmol) under nitrogen at 0 °C for 1 h, then warmed to room temperature and further stirred overnight. The reaction mixture was diluted with DCM (20 mL) and washed with saturated NaHCO_3_ solution (2 × 20 mL) and brine (1× 20 mL). The organic layer was dried (Na_2_SO_4_) and concentrated under reduced pressure. The yellow oil was purified by silica gel column chromatography (RediSep Gold silica gel column, 90–100% EtOAc in hexanes) to give the title compound as a light-yellow solid (8.4 mg, 81%). Mp: 251.3–251.9 °C. ^1^H NMR (400 MHz, CDCl_3_) δ 8.08 (s, 1H), 7.97 (s, 1H), 7.81 (s, 1H), 7.54 (s, 1H), 7.00 (s, 1H), 6.72 (s, 1H), 3.86 (s, 3H), 2.16 (s, 3H), 1.41 (s, 9H).

##### *tert*-Butyl 4-(4-Amino-2-fluorobenzamido)­piperidine-1-carboxylate (**5**)

To a solution of 4-amino-2-fluorobenzoic acid (1 equiv, 200.0 mg, 1.29 mmol) in DMF (5 mL) were added HATU (1.1 equiv, 539.2 mg, 1.42 mmol) and DIPEA (2.0 equiv, 0.449 mL, 2.58 mmol). The solution was stirred at room temperature for 30 min, followed by the addition of *tert*-butyl 4-aminopiperidine-1-carboxylate (1.2 equiv, 309.9 mg, 1.55 mmol). The reaction mixture was stirred at room temperature for an additional 12 h. The DMF was removed under reduced pressure, and the residue was diluted with EtOAc (50 mL). The organic layer was washed with brine (3 × 20 mL) and dried over Na_2_SO_4_. The solvent was removed, and the residue was purified by silica gel column chromatography (RediSep Gold silica gel column, 50–60% EtOAc in hexanes) to give the title compound as a pale-yellow solid (413 mg, 95%). Mp: 137.6–138.1 °C. ^1^H NMR (400 MHz, CDCl_3_) δ 7.89 (t, *J* = 8.9 Hz, 1H), 6.56–6.43 (m, 2H), 6.32 (dd, *J* = 2.2, 14.4 Hz, 1H), 4.06–4.28 (m, 3H), 4.03 (s, 2H), 2.95 (t, *J* = 12.1 Hz, 2H), 2.06–1.94 (m, 2H), 1.46 (s, 9H), 1.45–1.32 (m, 2H). ^13^C NMR (100 MHz, CDCl_3_) δ 162.8 (d, *J* = 3.6 Hz),162.2 (d, *J* = 244.4 Hz), 154.7, 151.5 (d, *J* = 12.7 Hz), 133.4 (d, *J* = 4.3 Hz), 110.9 (d, *J* = 1.9 Hz), 110.3 (d, *J* = 11.5 Hz), 100.8 (d, *J* = 28.9 Hz), 79.6, 46.8, 32.1, 29.3, 28.4. MS: calculated for C_17_H_24_FN_3_NaO_3_ [M + Na]^+^, 360.2; found 360.2.

##### *tert*-Butyl 4-(4-((4-((3-((1,1-Dimethylethyl)­sulfonamido)-5-(methoxycarbonyl)­phenyl)­amino)-5-methylpyrimidin-2-yl)­amino)-2-fluorobenzamido)­piperidine-1-carboxylate (**6**)

To a solution of *tert*-butyl 4-(4-amino-2-fluorobenzamido)­piperidine-1-carboxylate (**5**, 1 equiv, 16 mg, 48 μmol) and sodium *tert*-butoxide (2.0 equiv, 9.3 mg, 97 μmol) in 1,4-dioxane (0.5 mL), a solution of methyl 3-((2-chloro-5-methylpyrimidin-4-yl)­amino)-5-((1,1-dimethylethyl)­sulfonamido)­benzoate (**4**, 1 equiv, 20 mg, 48 μmol), Pd_2_(dba)_3_ (0.05 equiv, 2.2 mg, 2.4 μmol) and BINAP (0.1 equiv, 3.0 mg, 4.8 μmol) in 1,4-dioxane (0.5 mL) was added. The mixture was degassed and heated at 110 °C for 7 h. The mixture was cooled to room temperature, diluted with EtOAc, and the organic layer was washed with brine (3 × 20 mL) and dried over Na_2_SO_4_. The solvent was removed, and the residue was purified by silica gel column chromatography (RediSep Gold silica gel column, 10–20% MeOH in DCM, gradient) to give the title compound as an off-white solid (22 mg, 64%). Mp: 192.9.–193.4 °C. ^1^H NMR (400 MHz, MeOH-*d*_4_) δ 7.79 (d, *J* = 1.7 Hz, 1H), 7.77–7.67 (m, 3H), 7.56–7.36 (m, 2H), 7.07 (d, *J* = 8.5 Hz, 1H), 4.06–3.90 (m, 3H), 3.76 (s, 3H), 2.88 (s, 2H), 2.14 (s, 3H), 1.84 (dd, *J* = 13.3, 3.8 Hz, 2H), 1.49–1.39 (m, 2H), 1.38 (s, 9H), 1.26 (s, 9H).

##### 3-((2-((4-((1-(*tert*-Butoxycarbonyl)­piperidin-4-yl)­carbamoyl)-3-fluorophenyl)­amino)-5-methylpyrimidin-4-yl)­amino)-5-((1,1-dimethylethyl)­sulfonamido)­benzoic acid (**7**)

To a solution of *tert*-butyl 4-(4-((4-((3-((1,1-dimethylethyl)­sulfonamido)-5-(methoxycarbonyl)­phenyl)­amino)-5-methylpyrimidin-2-yl)­amino)-2-fluorobenzamido)­piperidine-1-carboxylate (**6**, 1 equiv, 10 mg, 14 μmol) in MeOH (0.125 mL) and THF (0.125 mL), a solution of LiOH (3 equiv, 1.0 mg, 42 μmol) in H_2_O (0.25 mL) was added. The reaction was stirred at 60 °C for 3 h. The mixture was cooled to room temperature, diluted with EtOAc (10 mL), washed with 2 *N* HCl, and the organic layer was washed with brine (3 × 20 mL) and dried over MgSO_4_. The solvent was removed, and the residue was purified by silica gel column chromatography (RediSep Gold silica gel column, 10–15% MeOH in DCM) to give the title compound as a gray solid (4.9 mg, 57%). Mp: 166.2–167.1 °C. Purity: 98% (HPLC). ^1^H NMR (400 MHz, DMSO-*d*_6_) δ 12.81­(brs, 1H), 9.89 (d, *J* = 2.8 Hz, 1H), 9.76 (s, 1H), 9.14 (s, 1H), 8.01 (s, 1H), 7.83 (s, 1H), 7.77 (d, *J* = 8.0 Hz 1H), 7.73 (s, 2H), 7.64 (d, *J* = 14.0 Hz, 1H), 7.42 (t, *J* = 8.5 Hz, 1H), 7.31 (d, *J* = 8.9 Hz, 1H), 3.96–3.84 (m, 3H), 2.87 (s, 2H), 2.16 (s, 3H), 1.78 (dd, *J* = 13.2, 3.9 Hz, 2H), 1.41 (s, 9H), 1.40–1.34 (m, 2H), 1.30 (s, 9H). ^13^C NMR (101 MHz, DMSO-*d*_6_) δ 172.0, 166.6, 162.6 (d, *J* = 2 Hz), 159.5 (d, *J* = 170 Hz), 158.2, 153.8, 152.9, 142.2 (d, *J* = 12 Hz), 140.8, 138.8, 131.9, 130.2 (d, *J* = 4 Hz), 120.4, 118.8, 117.1 (d, *J* = 13 Hz), 116.4, 114.3, 107.9, 105.7 (d, *J* = 29 Hz), 78.6, 61.1, 55.8, 46.3, 29.6, 28.1, 24.2, 13.4.

##### Methyl 3-((1,1-Dimethylethyl)­sulfonamido)-5-((2-((3-fluoro-4-(piperidin-4-ylcarbamoyl)­phenyl)­amino)-5-methylpyrimidin-4-yl)­amino)­benzoate (**8**)

To a solution of 3-((2-((4-((1-(*tert*-Butoxycarbonyl)­piperidin-4-yl)­carbamoyl)-3-fluorophenyl)­amino)-5-methylpyrimidin-4-yl)­amino)-5-((1,1-dimethylethyl)­sulfonamido)­benzoic acid (**7**, 1 equiv, 100 mg, 0.14 mmol) in DCM (0.8 mL) was added TFA (0.2 mL) and the mixture was stirred at room temperature for 12 h. The solvent was removed under reduced pressure, and then the pH was adjusted to pH = 13 by adding NaOH (1 *N*). The gray precipitate was filtered, washed with water (2 × 10 mL) and then with acetone (5 mL). The residue was dried under reduced pressure to give the title compound as a white solid (81 mg, 94%). Mp: 142.2–143.1 °C. Purity 97%. ^1^H NMR (400 MHz, MeOH-*d*_4_) δ 7.86 (dd, *J* = 2.2, 1.4 Hz, 1H), 7.78 (t, *J* = 1.7 Hz, 1H), 7.76 (d, *J* = 1.2 Hz, 1H), 7.59 (t, *J* = 2.1 Hz, 1H), 7.49 (t, *J* = 8.4 Hz, 1H), 7.35 (dd, *J* = 13.2, 2.1 Hz, 1H), 7.10 (dd, *J* = 8.5, 2.1 Hz, 1H), 4.14–4.01 (m, 1H), 3.76 (s, 3H), 3.38 (dt, *J* = 13.6, 4.1 Hz, 2H), 3.14–3.01 (m, 2H), 2.17 (d, *J* = 1.1 Hz, 3H), 2.11 (dd, *J* = 12.7, 5.1, 2H), 1.86–1.72 (m, 2H), 1.26 (s, 9H). MS: calculated for C_29_H_37_FN_7_O_5_S [M + H]^+^, 614.3; found 614.2.

#### General Procedure A

##### (3-((1,1-Dimethylethyl)­sulfonamido)-5-((2-((3-fluoro-4-(piperidin-4-ylcarbamoyl)­phenyl)­amino)-5-methylpyrimidin-4-yl)­amino)­benzoyl)­glycine (**9**)

To a solution of 3-((2-((4-((1-(*tert*-Butoxycarbonyl)­piperidin-4-yl)­carbamoyl)-3-fluorophenyl)­amino)-5-methylpyrimidin-4-yl)­amino)-5-((1,1-dimethylethyl)­sulfonamido)­benzoic acid (**7**, 1 equiv, 42 mg, 60 μmol), HATU (1.1 equiv, 25 mg, 67 μmol) in anhydrous DMF (1 mL), DIPEA (2.0 equiv, 21 μL, 121 μmol) was added. The solution was stirred at room temperature for 30 min, then glycine *tert*-butyl ester (1.2 equiv, 12 mg, 73 μmol) was added. The reaction solution was stirred at room temperature for 6 h. The mixture was diluted with EtOAc, and the organic layer was washed with brine (three times, 20 mL each) and dried over Na_2_SO_4_. The solvent was removed under reduced pressure. The residues were added to a mixture of DCM (0.8 mL) and TFA (0.2 mL) and stirred at room temperature for 2 h. The solvent was removed under reduced pressure, and the residue was purified by preparative HPLC to give the title compound as a white solid (29 mg, 73%). Mp: 216.7–217.4 °C. Purity >99%. ^1^H NMR (400 MHz, DMSO-*d*_6_) δ 10.44 (s, 1H), 9.91 (s, 1H), 9.58 (s, 1H), 8.72 (d, *J* = 10.3 Hz, 1H), 8.59 (d, *J* = 7.5 Hz, 1H), 8.46 (d, *J* = 10.7 Hz, 1H), 8.06 (s, 1H), 8.02 (dd, *J* = 7.5, 2.4 Hz, 1H), 7.87–7.77 (m, *J* = 1.7 Hz, 1H), 7.66 (t, *J* = 1.7 Hz, 1H), 7.61 (t, *J* = 2.0 Hz, 1H), 7.50 (dd, *J* = 13.5, 2.0 Hz, 1H), 7.40 (t, *J* = 8.4 Hz, 1H), 7.30 (dd, *J* = 8.6, 2.0 Hz, 1H), 4.35 (ddd, *J* = 9.6, 7.4, 5.0 Hz, 1H), 4.09–3.95 (m, 1H), 3.31 (d, *J* = 12.6 Hz, 2H), 3.11–2.94 (m, 2H), 2.26 (t, *J* = 7.7 Hz, 1H), 2.18 (s, 3H), 2.02–1.95 (m, 2H), 1.78–1.64 (m, 2H), 1.28 (s, 9H). MS: calculated for C_30_H_38_FN_8_O_6_S [M + H]^+^, 657.3; found 657.3.

##### (3-((1,1-Dimethylethyl)­sulfonamido)-5-((2-((3-fluoro-4-(piperidin-4-ylcarbamoyl)­phenyl)­amino)-5-methylpyrimidin-4-yl)­amino)­benzoyl)-L*-*phenylalanine (**10**)

The titled compound was produced following general procedure A using *tert*-butyl l-phenylalaninate, yielding a white solid (35 mg, 77%). Mp: 184.5–185.5 °C. Purity >99%. ^1^H NMR (400 MHz, MeOH-*d*_4_) δ 7.89–7.68 (m, 2H), 7.55 (s, 1H), 7.52–7.38 (m, 3H), 7.28 (d, *J* = 13.4, 1H), 7.12–7.00 (m, 5H), 4.65 (dd, *J* = 8.9, 5.1 Hz, 1H), 4.07 (tt, *J* = 10.2, 3.9 Hz, 1H), 3.37 (d, *J* = 13.5, 2H), 3.16 (dd, *J* = 13.8, 5.1 Hz, 1H), 3.07 (t, *J* = 12.8, Hz, 2H), 2.94 (dd, *J* = 13.9, 8.8 Hz, 1H), 2.15 (s, 3H), 2.13–2.07 (m, 2H), 1.84–1.74 (m, 2H), 1.26 (s, 9H). MS: calculated for C_37_H_44_FN_8_O_6_S [M + H]^+^, 747.3; found 747.2.

##### (3-((1,1-Dimethylethyl)­sulfonamido)-5-((2-((3-fluoro-4-(piperidin-4-ylcarbamoyl)­phenyl)­amino)-5-methylpyrimidin-4-yl)­amino)­benzoyl)-d-phenylalanine hydrochloride (**11**)

The titled compound was produced following general procedure A using *tert*-butyl d-phenylalaninate, yielding a white solid (35 mg, 77%). Mp: 189.7–190.2 °C. Purity >99%. ^1^H NMR (400 MHz, DMSO-*d*_6_) δ 10.00 (s, 1H), 9.84 (s, 1H), 9.16 (s, 1H), 8.66 (s, 1H), 8.59 (d, *J* = 7.9 Hz, 1H), 8.41 (d, *J* = 10.4 Hz, 1H), 8.07–7.99 (m, 2H), 7.71 (s, 1H), 7.65 (s, 1H), 7.58 (d, *J* = 1.9 Hz,1H), 7.50 (s, 1H), 7.44–7.30 (m, 2H), 7.24 (d, *J* = 7.2 Hz, 2H), 7.19–7.06 (m, 3H), 4.59– 4.53 (m, 1H), 4.01 (d, *J* = 8.2 Hz, 1H), 3.30 (d, *J* = 12.4 Hz, 2H), 3.14 (dd, *J* = 13.8, 4.6 Hz, 1H), 3.01 (dd, *J* = 13.3, 9.7 Hz, 3H), 2.16 (s, 3H), 2.00–1.94 (m, 2H), 1.73–1.64 (m, *J* = 10.4 Hz, 2H), 1.28 (s, 9H). ^13^C NMR (101 MHz, DMSO-*d*_6_) δ 172.9, 166.1, 163.1 (d, *J* = 2 Hz), 160.8, 160.1, 159.6 (d, *J* = 247 Hz), 158.3, 143.6 (d, *J* = 10 Hz), 140.4, 139.3, 137.9, 135.3, 130.2 (d, *J* = 4 Hz), 129.0, 128.0, 126.3, 117.6, 117.3, 116.0 (d, *J* = 15 Hz), 114.7, 113.7, 107.6, 104.9 (d, *J* = 28 Hz), 61.0, 54.3, 44.0, 42.1, 36.2, 28.1, 24.2, 13.4. MS: calculated for C_37_H_44_FN_8_O_6_S [M + H]^+^, 747.3; found 747.3.

##### (3-((1,1-Dimethylethyl)­sulfonamido)-5-((2-((3-fluoro-4-(piperidin-4-ylcarbamoyl)­phenyl)­amino)-5-methylpyrimidin-4-yl)­amino)­benzoyl)-l-threonine (**12**)

The titled compound was produced following general procedure A using *tert*-butyl L-allothreoninate, yielding a white solid (33 mg, 78%). Mp: 178.9.–179.4 °C. Purity >99% (HPLC). ^1^H NMR (400 MHz, MeOH-*d*_4_) δ 7.75 (s, 1H), 7.73 (s, 1H), 7.64 (s, 1H), 7.57 (s, 1H), 7.48 (t, *J* = 8.3 Hz, 1H), 7.33 (dd, *J* = 13.3, 2.1 Hz, 1H), 7.11 (dd, *J* = 8.4, 2.0 Hz, 1H), 4.50 (d, *J* = 3.0 Hz, 1H), 4.30 (tt, *J* = 9.5, 4.5 Hz, 1H), 4.09 (tt, *J* = 9.9, 4.1 Hz, 1H), 3.90 (s, 1H), 3.40 (dt, *J* = 13.4, 4.3 Hz, 2H), 3.09 (td, *J* = 11.8, 11.0, 3.6 Hz, 2H), 2.17 (s, 3H), 2.16–2.09 (m, 2H), 1.88–1.77 (m, 2H), 1.28 (s, 9H), 1.07 (d, *J* = 6.4 Hz, 3H). ^13^C NMR (101 MHz, DMSO-*d*_6_) δ 171.9, 166.0, 163.0 (d, *J* = 2 Hz), 159.4 (d, *J* = 261 Hz),158.9, 158.6, 158.2, 142.6 (d, *J* = 12 Hz), 140.7, 138.8, 135.2, 130.2 (d, *J* = 3 Hz), 118.2, 117.9, 116.8 (d, *J* = 14 Hz), 115.2, 114.2, 107.7, 105.6 (d, *J* = 2 Hz), 66.4, 61.0, 58.6, 44.1, 42.1, 28.1, 24.2, 20.3, 13.3. MS: calculated for C_32_H_42_FN_8_O_7_S [M + H]^+^, 701.3; found 701.6.

##### (3-((1,1-Dimethylethyl)­sulfonamido)-5-((2-((3-fluoro-4-(piperidin-4-ylcarbamoyl)­phenyl)­amino)-5-methylpyrimidin-4-yl)­amino)­benzoyl)-l-serine (**13**)

The titled compound was produced following general procedure A using *tert*-butyl l-serinate, yielding a white solid (30 mg, 72%). Mp: 144.4–145.3 °C. Purity 95% (HPLC). ^1^H NMR (400 MHz, DMSO-*d*_6_) δ 10.71 (s, 1H), 9.94 (s, 1H), 9.76 (s,1H), 8.81 (d, *J* = 11.0 Hz, 1H), 8.55 (d, *J* = 10.3 Hz, 1H), 8.32 (d, *J* = 7.6 Hz, 1H), 8.14–8.05 (m, 2H), 7.81 (s, 1H), 7.71 (t, *J* = 1.8 Hz, 1H), 7.63 (d, *J* = 2.0 Hz, 1H), 7.51–7.38 (m, 2H), 7.30 (d, *J* = 8.5, 1H), 4.53–4.40 (m, 1H), 4.02 (brs, 1H), 3.76 (m, 2H), 3.35 (d, *J* = 15.5 Hz, 1H), 3.14–2.99 (m, 4H), 2.19 (s, 3H), 1.98 (dd, *J* = 14.1, 4.1 Hz, 2H), 1.70 (q, *J* = 12.2, 10.7 Hz, 2H), 1.28 (s, 9H). ^13^C NMR (101 MHz, DMSO-*d*_6_) δ 171.7, 165.5, 163.0 (d, *J* = 2 Hz), 161.1, 159.5 (d, *J* = 211 Hz) 159.4, 152.6, 142.1 (d, *J* = 12 Hz), 140.7, 138.4, 135.2, 130.2 (d, *J* = 4 Hz), 118.7, 117.9, 117.3 (d, *J* = 15 Hz), 115.7, 114.5, 107.8, 106.0 (d, *J* = 27 Hz), 61.1, 61.0, 55.6, 44.1, 42.1, 28.0, 24.1, 13.3. MS: calculated for C_31_H_40_FN_8_O_7_S [M + H]^+^, 687.3; found 687.6.

##### (3-((1,1-Dimethylethyl)­sulfonamido)-5-((2-((3-fluoro-4-(piperidin-4-ylcarbamoyl)­phenyl)­amino)-5-methylpyrimidin-4-yl)­amino)­benzoyl)-l-glutamic Acid (**14**)

The titled compound was produced following general procedure A using di-*tert*-butyl l-glutamate, yielding a white solid (39 mg, 89%). Mp: 189.2–189.9 °C. Purity 98% (HPLC).^1^H NMR (400 MHz, MeOH-*d*_4_) δ 7.93 (s, 2H), 7.74 (d, *J* = 15.0 Hz, 1H), 7.66 (s, 1H), 7.63–7.50 (m, 2H), 7.17 (d, *J* = 8.5 Hz, 1H), 4.45 (q, *J* = 4.5 Hz, 1H), 4.18 (s, 1H), 3.99–3.81 (m, 2H), 3.47 (d, *J* = 13.7 Hz, 2H), 3.14 (t, *J* = 11.7 Hz, 2H), 2.70–2.59 (m, 2H), 2.22–2.19 (m, 5H), 1.98 (d, *J* = 12.6 Hz, 2H), 1.39 (s, 9H). ^13^C NMR (101 MHz, DMSO-*d*_6_) δ 172.3, 171.3, 165.0, 163.2 (d, *J* = 2 Hz), 161.0, 158.4 (d, *J* = 202 Hz), 158.6, 155.8, 145.0 (d, *J* = 11 Hz), 140.4, 140.3, 135.8, 130.0 (d, *J* = 4 Hz), 116.6, 116.2, 114.6 (d, *J* = 13 Hz), 113.3, 113.2, 107.2, 104.2 (d, *J* = 28 Hz), 60.9, 55.8, 44.4, 42.2, 32.1, 29.6, 28.7, 24.3, 13.6. MS: calculated for C_33_H_42_FN_8_O_8_S [M + H]^+^, 729.3; found 729.2.

##### (3-((1,1-Dimethylethyl)­sulfonamido)-5-((2-((3-fluoro-4-(piperidin-4-ylcarbamoyl)­phenyl)­amino)-5-methylpyrimidin-4-yl)­amino)­benzoyl)-l-glutamine (**15**)

The titled compound was produced following general procedure A using *tert*-butyl l-glutaminate, yielding a white solid (36 mg, 82%). Mp: 183.6.–183.9 °C. Purity >99% (HPLC). ^1^H NMR (400 MHz, MeOH-*d*_4_) δ 7.74 (s, 1H), 7.67 (dt, *J* = 9.4, 1.7 Hz, 2H), 7.52 (t, *J* = 2.0 Hz, 1H), 7.45 (t, *J* = 8.4 Hz, 1H), 7.29 (dd, *J* = 13.3, 2.1 Hz, 1H), 7.10 (dd, *J* = 8.5, 2.1 Hz, 1H), 4.40 (dd, *J* = 8.8, 4.9 Hz, 1H), 4.13–4.01 (m, 1H), 3.46–3.32 (m, 2H), 3.08 (t, *J* = 13.0, 2H), 2.29–2.05 (m, 8H), 1.94 (dt, *J* = 14.3, 9.0, 1H), 1.89–1.77 (m, 2H), 1.26 (s, 9H). MS: calculated for C_33_H_43_FN_8_O_8_S [M + H]^+^, 728.3; found 728.5.

##### 4-((4-((3-(*tert*-Butylcarbamoyl)-5-((1,1-dimethylethyl)­sulfonamido)­phenyl)­amino)-5-methylpyrimidin-2-yl)­amino)-2-fluoro-*N*-(piperidin-4-yl)­benzamide (**16**)

The titled compound was produced following general procedure A using 2-methylpropan-2-amine hydrochloride to yield a white solid (36 mg, 91%). Mp: 186.7–187.5 °C. Purity 96%. ^1^H NMR (400 MHz, DMSO-*d*_6_) δ 10.72 (s, 1H), 9.88 (s, 1H), 9.73 (s, 1H), 8.77 (d, *J* = 11.0 Hz, 1H), 8.55 (t, *J* = 10.6 Hz, 1H), 8.18–8.00 (m, 2H), 7.76 (s, 1H), 7.66–7.49 (m, 3H), 7.49–7.38 (m, 2H), 7.33 (dd, *J* = 8.6, 1.9 Hz, 1H), 4.09–3.95 (m, 1H), 3.31 (d, *J* = 12.3 Hz, 2H), 3.03 (q, *J* = 11.3 Hz, 2H), 2.18 (s, 3H), 2.04–1.92 (m, 2H), 1.78–1.61 (m, 2H), 1.29 (s, 18H). ^13^C NMR (101 MHz, DMSO-*d*_6_) δ 165.3, 162.9 (d, *J* = 2 Hz), 162.0 (d, *J* = 204 Hz), 160.5, 158.1, 152.6, 142.2 (d, *J* = 12 Hz), 140.4, 138.1, 136.9, 130.3 (d, *J* = 4 Hz), 124.1, 118.7, 117.2 (d, *J* = 13 Hz), 115.8, 114.3, 107.8, 105.7 (d, *J* = 14 Hz), 61.0, 50.8, 44.1, 42.1, 28.4, 28.1, 24.2, 13.3. MS: calculated for C_32_H_44_FN_8_O_4_S [M + H]^+^, 655.3; found 655.3.

##### 4-((4-((3-((1,1-Dimethylethyl)­sulfonamido)-5-(phenethylcarbamoyl)­phenyl)­amino)-5-methylpyrimidin-2-yl)­amino)-2-fluoro-*N*-(piperidin-4-yl)­benzamide (**17**)

The titled compound was produced following general procedure A using 2-phenylethan-1-amine, yielding a white solid (28 mg, 67%). Mp: 124.2.–124.4 °C. Purity 98%. ^1^H NMR (400 MHz, DMSO-*d*_6_) δ 10.86 (s, 1H), 9.94 (s, 1H), 9.87 (s, 1H), 8.75 (d, *J* = 11.2 Hz, 1H), 8.62–8.42 (m, 2H), 8.11­(d, *J* = 4 Hz, 1H), 8.07 (s, 1H), 7.76 (s, 1H), 7.71 (d, *J* = 2.1 Hz, 1H), 7.50 (s, 1H), 7.45–7.34 (m, 2H), 7.32–7.22 (m, 3H), 7.20– 7.14 (m, 3H), 4.04–3.95 (m, 1H), 3.44–3.36 (m, 2H), 3.29 (d, *J* = 12.6 Hz, 2H), 3.01­(d, *J* = 11.2 Hz, 2H), 2.72 (t, *J* = 7.6 Hz, 2H), 2.19 (s, 3H), 1.98–1.90 (m, 2H), 1.74–1.62 (m, 2H), 1.28 (s, 9H). ^13^C NMR (101 MHz, DMSO-*d*_6_) δ 165.3, 162.9 (d, *J* = 2 Hz), 161.2, 159.2 (d, *J* = 247 Hz), 153.8, 152.0, 141.8 (d, *J* = 12 Hz), 140.6, 139.4, 138.1, 135.8, 130.2 (d, *J* = 4 Hz), 128.5, 128.2, 126.0, 124.1, 119.0, 117.6 (d, *J* = 15 Hz), 116.1, 114.7,, 107.9, 106.3 (d, *J* = 28 Hz), 61.1, 44.1, 42.1, 40.9, 35.0, 28.0, 24.2, 13.3. MS: calculated for C_36_H_44_FN_8_O_4_S [M + H]^+^, 703.3; found 703.2.

##### Methyl (3-((1,1-Dimethylethyl)­sulfonamido)-5-((2-((3-fluoro-4-(piperidin-4-ylcarbamoyl)­phenyl)­amino)-5-methylpyrimidin-4-yl)­amino)­benzoyl)­glycinate (**18**)

The titled compound was produced following general procedure A using methyl glycinate, yielding a white solid (34 mg, 84%). Mp: 152.1–152.7 °C. Purity 94% (HPLC).^1^H NMR (400 MHz, DMSO-*d*_6_) δ 10.09 (s, 1H), 9.89 (s, 1H), 9.31 (s, 1H), 8.86 (t, *J* = 5.8 Hz, 1H), 8.71–8.58 (m, 1H), 8.40 (d, *J* = 10.6 Hz, 1H), 8.04 (s, 2H), 7.81 (s, 1H), 7.62 (d, *J* = 12.4, 2H), 7.53 (d, *J* = 13.5 Hz, 1H), 7.45–7.30 (m, 2H), 4.06–3.99 (m, 1H), 3.94 (d, *J* = 8.0, 2H), 3.63 (s, 3H), 3.31 (d, *J* = 12.5 Hz, 2H), 3.03 (q, *J* = 11.4 Hz, 2H), 2.17 (s, 3H), 1.98 (dd, *J* = 13.8, 3.9 Hz, 2H), 1.74–1.65 (m, 2H), 1.29 (s, 9H). ^13^C NMR (101 MHz, DMSO-*d*_6_) δ 170.2, 166.1, 163.1 (d, *J* = 2 Hz), 161.5, 160.7, 159.3 (d, *J* = 215 Hz), 154.6, 143.9 (d, *J* = 13 Hz), 140.5, 139.2, 134.9, 130.2 (d, *J* = 4 Hz), 118.1, 117.8, 116.2 (d, *J* = 11 Hz), 115.0, 114.0, 107.6, 105.3 (d, *J* = 29 Hz), 61.0, 51.7, 44.0, 42.2, 41.3, 28.1, 24.2, 13.4. MS: calculated for C_31_H_40_FN_8_O_6_S [M + H]^+^, 671.3; found 671.2.

##### 3-(3-((1,1-Dimethylethyl)­sulfonamido)-5-((2-((3-fluoro-4-(piperidin-4-ylcarbamoyl)­phenyl)­amino)-5-methylpyrimidin-4-yl)­amino)­benzamido)­propanoic Acid (**19**)

The titled compound was produced following general procedure A using *tert*-butyl 3-aminopropanoate hydrochloride glycinate, yielding a white solid (36 mg, 90%). Mp: 195.1–195.8 °C. Purity >99%. ^1^H NMR (400 MHz, DMSO-*d*_6_) δ 10.52 (s, 1H), 9.77 (s, 1H), 9.58 (s, 1H), 8.62 (d, *J* = 11.0 Hz, 1H), 8.47–8.27 (m, 2H), 8.04–7.89 (m, 2H), 7.64 (t, *J* = 1.6 Hz, 1H), 7.54 (t, *J* = 1.8 Hz, 1H), 7.39 (t, *J* = 1.9 Hz, 1H), 7.35–7.21 (m, 2H), 7.16 (dd, *J* = 8.6, 2.0 Hz, 1H), 3.95–3.81 (m, 1H), 3.26 (q, *J* = 7.0 Hz, 2H), 3.18 (d, *J* = 12.4 Hz, 2H), 2.90 (q, *J* = 11.3 Hz, 2H), 2.30 (t, *J* = 7.2 Hz, 2H), 2.04 (s, 3H), 1.90–1.80 (m, 2H), 1.65–1.48 (m, 2H), 1.14 (s, 9H). ^13^C NMR (101 MHz, DMSO-*d*_6_) δ 172.8, 165.4, 163.0 (d, *J* = 2 Hz), 159.9 (d, *J* = 218 Hz), 158.5, 158.0, 153.8, 142.2 (d, *J* = 12 Hz), 140.6, 138.3, 135.5, 130.1 (d, *J* = 4 Hz) 124.1, 118.7, 117.2 (d, *J* = 17 Hz), 115.8, 114.5, 107.8, 106.1 (d, *J* = 29 Hz), 61.0, 44.0, 42.0, 35.6, 33.6, 28.1, 24.2, 13.3. MS: calculated for C_31_H_40_FN_8_O_6_S [M + H]^+^, 671.3; found 671.4.

##### 4-(3-((1,1-Dimethylethyl)­sulfonamido)-5-((2-((3-fluoro-4-(piperidin-4-ylcarbamoyl)­phenyl)­amino)-5-methylpyrimidin-4-yl)­amino)­benzamido)­butanoic acid (**20**)

The titled compound was produced following general procedure A using *tert*-butyl 4-aminobutanoate hydrochloride, yielding a white solid (29 mg, 71%). Mp: 179.4–180.1 °C. Purity 96%. ^1^H NMR (400 MHz, DMSO-*d*_6_) δ 10.51 (s, 1H), 9.88 (s, 1H), 9.61 (s, 1H), 8.71 (d, *J* = 11.0 Hz, 1H), 8.53–8.36 (m, 2H), 8.14–8.00 (m, 2H), 7.79 (s, 1H), 7.66 (s, 1H), 7.53 (s, 1H), 7.44 (d, *J* = 12.0 Hz, 1H), 7.38 (t, *J* = 8.3 Hz, 1H), 7.30 (dd, *J* = 8.6, 1.9 Hz, 1H), 4.06–3.98 (m, 1H), 3.32 (d, *J* = 12.6 Hz, 2H), 3.20 (q, *J* = 6.6 Hz, 2H), 3.03 (q, *J* = 11.3 Hz, 2H), 2.22–2.10 (m, 5H), 2.04–1.93 (m, 2H), 1.80–1.59 (m, 4H), 1.28 (s, 9H). ^13^C NMR (101 MHz, DMSO-*d*_6_) δ 174.2, 165.5, 163.0 (d, *J* = 2 Hz) 159.8 (d, *J* = 202 Hz), 158.8, 158.4, 158.0, 142.4 (d, *J* = 12 Hz), 140.5, 138.5, 135.7, 130.1 (d, *J* = 4 Hz), 124.1, 118.6, 117.0 (d, *J* = 14 Hz), 115.6, 114.4, 107.7, 105.9 (d, *J* = 27 Hz), 61.0, 44.1, 42.1, 38.7, 31.0, 28.1, 24.3, 24.2, 13.3. MS: calculated for C_32_H_42_FN_8_O_6_S [M + H]^+^, 685.3; found 685.3.

##### (3-((1,1-Dimethylethyl)­sulfonamido)-5-((2-((3-fluoro-4-(piperidin-4-ylcarbamoyl)­phenyl)­amino)-5-methylpyrimidin-4-yl)­amino)­benzamido)­methanesulfonic Acid Hydrochloride (**21**)

The titled compound was produced following general procedure A using aminomethanesulfonic acid, yielding a white solid (28 mg, 68%). Mp: 284.3–285.0 °C. Purity 97% (HPLC). ^1^H NMR (400 MHz, DMSO-*d*_6_) δ 10.41 (s, 1H), 9.98 (s, 1H), 9.50 (s, 1H), 8.81 (s, 1H), 8.57 (s, 1H), 8.31–8.01 (m, 2H), 7.92 (s, 1H), 7.78 (s, 1H), 7.68 (s, 1H), 7.58 (d, *J* = 13.3 Hz, 1H), 7.39 (t, *J* = 8.0 Hz, 1H), 7.24 (d, *J* = 9.1 Hz, 1H), 4.01 (s, 1H), 3.77 (s, 2H), 3.30 (d, *J* = 11.5 Hz, 2H), 3.01 (d, *J* = 11.8 Hz, 2H), 2.16 (s, 3H), 1.96 (d, *J* = 12.3 Hz, 2H), 1.68 (d, *J* = 11.6 Hz, 2H), 1.28 (s, 9H). ^13^C NMR (101 MHz, DMSO-*d*_6_) δ 165.0, 163.1 (d, *J* = 2 Hz), 160.7, 160.6, 159.4 (d, *J* = 251 Hz), 153.6, 142.6 (d, *J* = 10 Hz), 140.4, 138.7, 135.7, 130.2 (d, *J* = 4 Hz), 118.6, 117.7, 116.8 (d, *J* = 11 Hz), 115.6, 114.4, 107.7, 105.8 (d, *J* = 27 Hz), 61.0, 55.9, 43.9, 42.0, 27.9, 24.2, 13.4. MS: calculated for C_29_H_36_FN_8_O_7_S_2_ [M – H]^−^, 691.2; found 691.5.

##### 4-((4-((3-((1,1-Dimethylethyl)­sulfonamido)-5-((2-sulfamoylethyl)­carbamoyl)­phenyl)­amino)-5-methylpyrimidin-2-yl)­amino)-2-fluoro-*N*-(piperidin-4-yl)­benzamide Hydrochloride (**22**)

The titled compound was produced following general procedure A using aminomethanesulfonamide, yielding a white solid (39 mg, 91%). Mp: 187.0–187.6 °C. Purity >99%. ^1^H NMR (400 MHz, DMSO-*d*_6_) δ 10.27 (s, 1H), 9.89 (s, 1H), 9.43 (s, 1H), 8.70 (d, *J* = 11.0 Hz, 1H), 8.55 (t, *J* = 5.5 Hz, 1H), 8.44 (d, *J* = 9.7 Hz, 1H), 8.15–8.01 (m, 2H), 7.80 (s, 1H), 7.64 (s, 1H), 7.60–7.49 (m, 2H), 7.44–7.28 (m, 2H), 6.92 (s, 2H), 4.10–3.96 (m, 1H), 3.66–3.51 (m, 2H), 3.31 (d, *J* = 12.6 Hz, 2H), 3.15 (t, *J* = 6.0 Hz, 2H), 3.03 (q, *J* = 11.3 Hz, 2H), 2.17 (s, 3H), 2.03–1.95 (m, 2H), 1.69 (q, *J* = 10.9 Hz, 2H), 1.29 (s, 9H). ^13^C NMR (101 MHz, DMSO-*d*_6_) δ 165.6, 163.1 (d, *J* = 3 Hz), 160.6, 160.6, 159.3 (d, *J* = 235 Hz), 154.1, 143.0 (d, *J* = 11 Hz), 140.5, 138.9, 135.1, 130.1 (d, *J* = 3 Hz), 118.2, 117.7, 116.5 (d, *J* = 12 Hz), 115.1, 114.1, 107.6, 105.6 (d, *J* = 27 Hz), 61.03, 53.4, 44.1, 42.1, 34.7, 28.1, 24.2, 13.4. MS: calculated for C_30_H_41_FN_9_O_6_S_2_ [M + H]^+^, 706.3; found 706.2.

##### 4-((4-((3-((2-Amino-2-oxoethyl)­carbamoyl)-5-((1,1-dimethylethyl)­sulfonamido)­phenyl)­amino)-5-methylpyrimidin-2-yl)­amino)-2-fluoro-*N*-(piperidin-4-yl)­benzamide Hydrochloride (**23**)

The titled compound was produced following general procedure A using 2-aminoacetamide, yielding a pale-yellow solid (29 mg, 73%). Mp: 286.6–287.6 °C. Purity >99%. ^1^H NMR (400 MHz, DMSO-*d*_6_) δ 10.66 (s, 1H), 9.93 (s, 1H), 9.76 (s, 1H), 8.74 (s, 1H), 8.59–8.40 (m, 2H), 8.16–8.04 (m, 2H), 7.80 (s, 1H), 7.71 (s, 1H), 7.56 (s, 1H), 7.52–7.34 (m, 3H), 7.27 (dd, *J* = 8.6, 1.9 Hz, 1H), 7.04 (s, 1H), 4.10–3.97 (m, 1H), 3.78 (d, *J* = 5.7 Hz, 2H), 3.31 (d, *J* = 12.3 Hz, 2H), 3.03 (q, *J* = 11.5 Hz, 2H), 2.18 (s, 3H), 2.08–1.94 (m, 2H), 1.81–1.57 (m, 2H), 1.28 (s, 9H). ^13^C NMR (101 MHz, DMSO- *d*_6_) δ 170.7, 165.5, 163.0 (d, *J* = 2 Hz), 161.1, 159.5 (d, *J* = 195 Hz, 2H), 158.9, 158.0, 142.1 (d, *J* = 11 Hz), 140.6, 138.4, 135.4, 130.2 (d, *J* = 4 Hz), 118.9, 118.2, 117.3 (d, *J* = 14 Hz), 115.8, 114.9, 107.8, 106.1 (d, *J* = 28 Hz), 61.0, 44.1, 42.4, 42.1, 28.1, 24.2, 13.3. MS: calculated for C_30_H_39_FN_9_O_5_S [M + H]^+^, 656.3; found 656.3.

##### (3-((1,1-Dimethylethyl)­sulfonamido)-5-((2-((3-fluoro-4-(piperidin-4-ylcarbamoyl)­phenyl)­amino)-5-methylpyrimidin-4-yl)­amino)­benzoyl)-L-homoserine (**24**)

The titled compound was produced following general procedure A using *tert*-butyl l-homoserinate, yielding a white solid (26 mg, 61%). ^1^H NMR (400 MHz, DMSO-*d*_6_) δ 9.53 (s, 1H), 8.66 (s, 1H), 8.21 (s, 1H), 8.14 (d, *J* = 5.9 Hz, 1H), 7.98 (s, 1H), 7.92–7.84 (m, 2H), 7.58 (d, *J* = 20.4 Hz, 2H), 7.46 (s, 1H), 7.40 (t, *J* = 8.6 Hz, 2H), 4.01 (d, *J* = 6.0 Hz, 2H), 3.98 (s, 1H), 3.49 (tt, *J* = 11.0, 5.9 Hz, 4H), 2.92 (t, *J* = 11.5 Hz, 3H), 2.16 (s, 3H), 1.92 (s, 2H), 1.85 (tt, *J* = 14.0, 7.1 Hz, 3H), 1.75 (d, *J* = 10.1 Hz, 1H), 1.71 (s, 1H), 1.30 (s, 9H). MS: calculated for C_32_H_41_FN_8_O_7_S [M + H]^+^, 701.3; found 701.3.

##### (*R*)-3-(3-((1,1-Dimethylethyl)­sulfonamido)-5-((2-((3-fluoro-4-(piperidin-4-ylcarbamoyl)­phenyl)­amino)-5-methylpyrimidin-4-yl)­amino)­benzamido)-4-hydroxybutanoic Acid (**25**)

The titled compound was produced following general procedure A using *tert-*butyl (*R*)-3-amino-4-hydroxybutanoate, yielding a white solid (30 mg, 72%). ^1^H NMR (400 MHz, DMSO-*d*_6_) δ 10.14 (s, 1H), 9.69 (s, 1H), 9.43 (s, 1H), 8.62 (d, *J* = 11.0 Hz, 1H), 8.48 (m, 2H), 7.98 (s, 1H), 7.83 (m, 2H), 7.51 (d, *J* = 19.6 Hz, 2H), 7.40 (t, *J* = 8.7 Hz, 1H), 4.00 (s, 1H), 3.47 (dd, *J* = 10.1, 4.9 Hz, 3H), 3.16 (s, 2H), 2.85 (s, 1H), 2.42 (dd, *J* = 15.3, 4.2 Hz, 3H), 2.29 (s, 1H), 2.15 (s, 3H), 1.88 (d, *J* = 13.6 Hz, 2H), 1.67 (s, 1H), 1.28 (s, 9H), 1.24 (s, 2H), 1.11 (d, *J* = 1.8 Hz, 1H). MS: calculated for C_32_H_41_FN_8_O_7_S [M + H]^+^, 701.2; found 701.3.

##### 3-(2-(2-(3-((1,1-Dimethylethyl)­sulfonamido)-5-((2-((3-fluoro-4-(piperidin-4-ylcarbamoyl)­phenyl)­amino)-5-methylpyrimidin-4-yl)­amino)­benzamido)­ethoxy)­ethoxy)­propanoic Acid (**26**)

The titled compound was produced following general procedure A using *tert*-butyl 3-(2-(2-aminoethoxy)­ethoxy)­propanoate, yielding a white solid (30 mg, 66%). ^1^H NMR (400 MHz, DMSO-*d*_6_) δ10.30 (s, 1H), 9.46 (s, 1H), 8.77 (s, 1H), 8.40 (t, *J* = 5.6 Hz, 1H), 7.98 (m, 2H), 7.82 (d, *J* = 1.7 Hz, 1H), 7.75 (dd, *J* = 7.4, 3.5 Hz, 1H), 7.67–7.59 (m, 2H), 7.57 (t, *J* = 1.8 Hz, 2H), 7.37 (d, *J* = 4.2 Hz, 2H), 4.30 (s, 1H), 3.89 (s, 1H), 3.57 (t, *J* = 6.5 Hz, 3H), 3.44 (p, *J* = 5.0 Hz, 8H), 3.33 (q, *J* = 5.8 Hz, 3H), 2.74 (t, *J* = 11.3 Hz, 2H), 2.26 (t, *J* = 6.4 Hz, 2H), 1.89–1.82 (m, 2H), 1.60 (q, *J* = 11.8 Hz, 2H), 1.28 (s, 9H). MS: calculated for C_35_H_47_FN_8_O_8_S [M + H]^+^, 759.3; found 759.4.

##### 8-(3-((1,1-Dimethylethyl)­sulfonamido)-5-((2-((3-fluoro-4-(piperidin-4-ylcarbamoyl)­phenyl)­amino)-5-methylpyrimidin-4-yl)­amino)­benzamido)­octanoic Acid (**27**)

The titled compound was produced following general procedure A using *tert*-butyl 8-aminooctanoate, yielding a pale-yellow solid (26 mg, 59%). ^1^H NMR (400 MHz, DMSO-*d*_6_) δ 9.46 (s, 1H), 8.68 (s, 1H), 8.27 (t, *J* = 5.6 Hz, 1H), 7.98 (s, 1H), 7.79 (d, *J* = 1.8 Hz, 1H), 7.69–7.56 (m, 3H), 7.49 (t, *J* = 1.8 Hz, 1H), 7.37 (d, *J* = 4.4 Hz, 2H), 3.15 (q, *J* = 6.6 Hz, 2H), 3.03 (s, 1H), 2.64 (t, *J* = 11.6 Hz, 2H), 2.11 (d, *J* = 14.3 Hz, 5H), 1.80 (d, *J* = 12.3 Hz, 2H), 1.49 (d, *J* = 12.3 Hz, 2H), 1.45–1.37 (m, 3H), 1.28 (s, 9H), 1.21 (m, 5H). MS: calculated for C_36_H_49_FN_8_O_6_S [M + H]^+^, 741.3; found 741.4.

##### 7-(3-((1,1-Dimethylethyl)­sulfonamido)-5-((2-((3-fluoro-4-(piperidin-4-ylcarbamoyl)­phenyl)­amino)-5-methylpyrimidin-4-yl)­amino)­benzamido)­heptanoic Acid (**28**)

The titled compound was produced following general procedure A using *tert*-butyl 7-aminoheptanoate, yielding a white solid (30 mg, 69%). ^1^H NMR (400 MHz, DMSO-*d*_6_) δ 9.45 (s, 1H), 8.70 (s, 1H), 8.28 (t, *J* = 5.7 Hz, 1H), 7.98 (s, 1H), 7.79 (d, *J* = 1.7 Hz, 1H), 7.65–7.53 (m, 3H), 7.50 (d, *J* = 1.8 Hz, 1H), 7.35 (d, *J* = 7.3 Hz, 2H), 3.80 (s, 2H), 3.14 (q, *J* = 6.7 Hz, 2H), 3.02 (s, 1H), 2.61 (t, *J* = 11.7 Hz, 2H), 2.15–2.04 (m, 5H), 1.80 (d, *J* = 12.3 Hz, 2H), 1.51–1.38 (m, 5H), 1.28 (m, 13H). MS: calculated for C_35_H_47_FN_8_O_6_S [M + H]^+^, 727.3; found 727.3.

##### 3-(2-(3-((1,1-Dimethylethyl)­sulfonamido)-5-((2-((3-fluoro-4-(piperidin-4-ylcarbamoyl)­phenyl)­amino)-5-methylpyrimidin-4-yl)­amino)­benzamido)­ethoxy)­propanoic Acid (**29**)

The titled compound was produced following general procedure A using *tert*-butyl 3-(2-aminoethoxy)­propanoate, yielding a white solid (30 mg, 70%). ^1^H NMR (400 MHz, DMSO-*d*_6_) δ 9.50 (s, 1H), 8.74 (s, 1H), 8.38 (t, *J* = 5.6 Hz, 1H), 7.97 (s, 1H), 7.84 (d, *J* = 1.7 Hz, 1H), 7.72 (dd, *J* = 7.3, 3.8 Hz, 1H), 7.65–7.58 (m, 1H), 7.54 (dt, *J* = 14.8, 1.9 Hz, 2H), 7.35 (d, *J* = 7.1 Hz, 2H), 4.40 (s, 1H), 3.88 (s, 1H), 3.58 (t, *J* = 6.4 Hz, 2H), 3.41 (t, *J* = 6.3 Hz, 2H), 3.30 (q, *J* = 6.1 Hz, 2H), 2.75 (t, *J* = 11.6 Hz, 2H), 2.22 (t, *J* = 6.4 Hz, 2H), 2.13 (s, 3H), 1.92–1.84 (m, 2H), 1.61 (q, *J* = 11.4 Hz, 2H), 1.29 (s, 9H). MS: calculated for C_33_H_43_FN_8_O_7_S [M + H]^+^, 715.3; found 715.2.

##### 6-(3-((1,1-Dimethylethyl)­sulfonamido)-5-((2-((3-fluoro-4-(piperidin-4-ylcarbamoyl)­phenyl)­amino)-5-methylpyrimidin-4-yl)­amino)­benzamido)­hexanoic Acid (**30**)

The titled compound was produced following general procedure A using *tert*-butyl 6-aminohexanoate, yielding a white solid (26 mg, 60%). ^1^H NMR (400 MHz, DMSO-*d*_6_) δ 9.46 (s, 1H), 8.69 (s, 1H), 8.30 (t, *J* = 5.7 Hz, 1H), 7.98 (s, 1H), 7.81 (s, 1H), 7.66 (dd, *J* = 7.5, 3.9 Hz, 1H), 7.59 (d, *J* = 14.4 Hz, 1H), 7.54 (d, *J* = 2.0 Hz, 1H), 7.49 (t, *J* = 1.8 Hz, 1H), 7.35 (d, *J* = 5.1 Hz, 2H), 4.29 (s, 1H), 3.84 (d, *J* = 9.2 Hz, 1H), 3.10 (dd, *J* = 16.8, 9.5 Hz, 4H), 2.68 (t, *J* = 11.6 Hz, 2H), 2.13 (s, 3H), 2.05 (t, *J* = 7.2 Hz, 2H), 1.84 (d, *J* = 12.4 Hz, 2H), 1.55 (d, *J* = 11.7 Hz, 1H), 1.52–1.35 (m, 5H), 1.29 (m, 11H). MS: calculated for C_34_H_45_FN_8_O_6_S [M + H]^+^, 713.3; found 713.3.

## Supplementary Material





## Notes

Dose–response curve of compound analogs binding to BRDT-BD1 and BRD4-BD1 by fluorescence polarization (Figure S1); electron density maps of compound 14 bound to BRD4 and BRDT, (A) **14** in BRD4-BD1 (PDB 7MR8), (B) **14** in BRDT-BD1 (PDB 9YCQ), 2Fo-Fc density maps upon refinement with ligand are shown in blue (contoured at 1σ), Fo–Fc density maps upon refinement omitting the ligand are shown in red (contoured at 3σ) (Figure S2); AlphaScreen IC_50_ data and selectivity ratios for compounds **8**-**23** (Table S1); AlphaScreen BRDT, BRD4 activity data for **SG3–179** and compounds **13** and **14** (Table S2); crystallographic data collection and refinement statistics (Table S3); permeability Results of Test Compounds in MDCK-MDR1 Cells (Table S4); the assessment of MDCK-MDR1 Cell Monolayer Integrity (Table S5); NMR spectra and HPLC traces for purity determination (PDF)
Molecular formula strings (CSV)

## Abbreviations Used

ACN: acetonitrile
AML: acute myeloid leukemia
BD1: first bromodomain
BD2: second bromodomain
BET: bromodomain and extra-terminal
BINAP: 2,2′-Bis­(diphenylphosphino)-1,1′-binaphthyl
BRD2: bromodomain-containing protein 2
BRD3: bromodomain-containing protein 3
BRD4: bromodomain-containing protein 4
BRDT: bromodomain testis-specific protein
CHAPS: 3-((3-Cholamidopropyl)­dimethylammonio)-1-propanesulfonate
CL_int_: intrinsic clearance
DCM: dichloromethane
DIPEA: *N*,*N*-Diisopropylethylamine
DMF: *N*,*N*-Dimethylformamide
DMSO: dimethyl sulfoxide
DNA: deoxyribonucleic acid
DTT: dithiothreitol
ECL: enhanced chemiluminescence
EDC: *N*-(3-(dimethylamino)­propyl)-*N*′-ethylcarbodiimide
ET: extra-terminal
FLT3: Fms-like tyrosine kinase 3
FP: fluorescence polarization
FRET: fluorescence resonance energy transfer
Fu, mic: fraction unbound in microsomes
GAPDH: glyceraldehyde 3-phosphate dehydrogenase
GWAS: genome-wide association studies
HATU: *O*-(7-azabenzotriazol-1-yl)-*N*,*N*,*N*′,*N*′-tetramethyluronium hexafluorophosphate
HDL: high-density lipoprotein
HEPES: 4-(2-Hydroxyethyl)-1-piperazineethanesulfonic acid
HOBt: 1-hydroxybenzotriazole
HPLC: high-performance liquid chromatography
HRP: Horseradish peroxidase
IC_50_: half maximal inhibitory concentration
IPA: isopropyl alcohol
JAK2: Janus kinase 2
Kac: acetylated lysine
*K*_D_: dissociation constant
*K_i_*: inhibition constant
LiOH: lithium hydroxide
mCPBA: *meta*-chloroperoxybenzoic acid
MeOH: methanol
MM: multiple myeloma
MPLC: medium-pressure liquid chromatography
MS: mass spectrometry
NMR: nuclear magnetic resonance
PEG: poly­(ethylene glycol)
PrOF: protein-observed fluorine
RT: room temperature
SAR: structure–activity relationship
SEM: standard error of the mean
SNP: single-nucleotide polymorphism
TFA: trifluoroacetic acid
THF: tetrahydrofuran
TLC: thin-layer chromatography
UPLC: ultraperformance liquid chromatography
UV: ultraviolet
WPF: tryptophan-proline-phenylalanine
