# Supplementary material for: Structural Basis for BD1-Preferring 2,4-Disubstituted Pyrimidine BRDT Inhibitors
Source: J Med Chem. 2026 Apr 15;69(9):11088–108. doi: 10.1021/acs.jmedchem.6c00180 (PMC13181795; doi:10.1021/acs.jmedchem.6c00180)
Supplement: Supplementary file 1 [file jm6c00180_si_001.pdf]

Supporting Information

Structural Basis for BD1-Preferring 2,4-Disubstituted Pyrimidine BRDT Inhibitors

Taimeng Liang,[1#] Xianghong Guan,[2#] Alice Chan,[3] Prakriti Kalra,[1] Rui Shi,[2] Jonathan Solberg,[2] Logan H. Sigua,[4] Jun Qi,[4] William C. K. Pomerantz,[1] Ernst Schönbrunn,[3] Jon E. Hawkinson,[2] and Gunda I. Georg[2*]

[1]Department of Chemistry, University of Minnesota – Twin Cities, Minneapolis, MN, United States

[2]Department of Medicinal Chemistry and Institute for Therapeutics Discovery and Development, University of Minnesota College of Pharmacy – Twin Cities, Minneapolis, MN, United States

[3]Drug Discovery Department, Moffitt Cancer Center, Tampa, FL, United States

[4]Department of Medical Oncology, Dana-Farber Cancer Institute, and Department of Medicine, Harvard Medical School, 360 Longwood Avenue, Boston, Massachusetts 02215, United States

[#]These authors contributed equally

*Corresponding author

Table of Contents

Supplementary Figures S1-S2

Tables S1-S5

NMR Spectra

HPLC Traces for Purity Determination

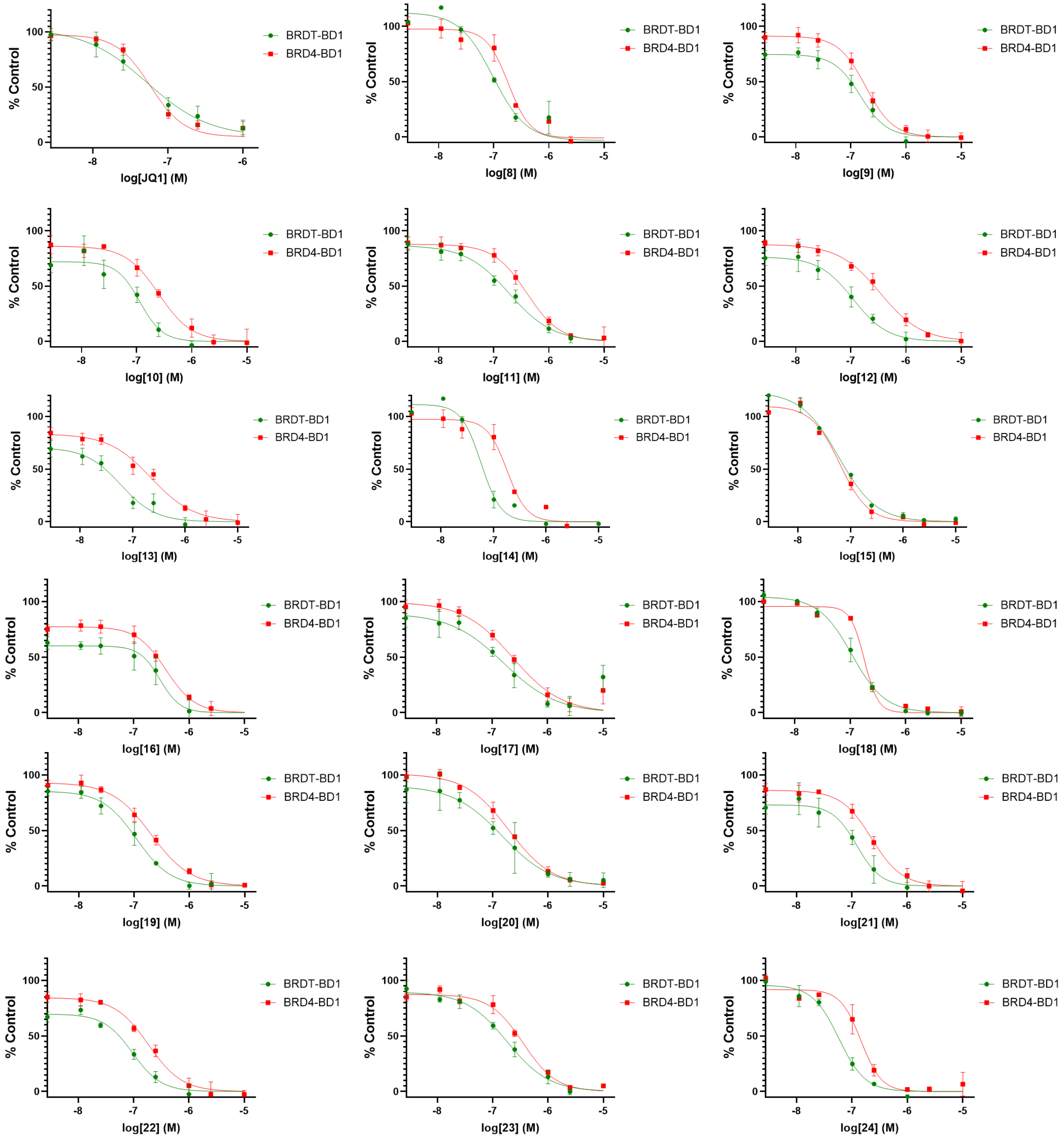

Figure S1. Dose-response curve of compound analogues binding to BRDT-BD1 and BRD4-BD1 by fluorescence polarization.

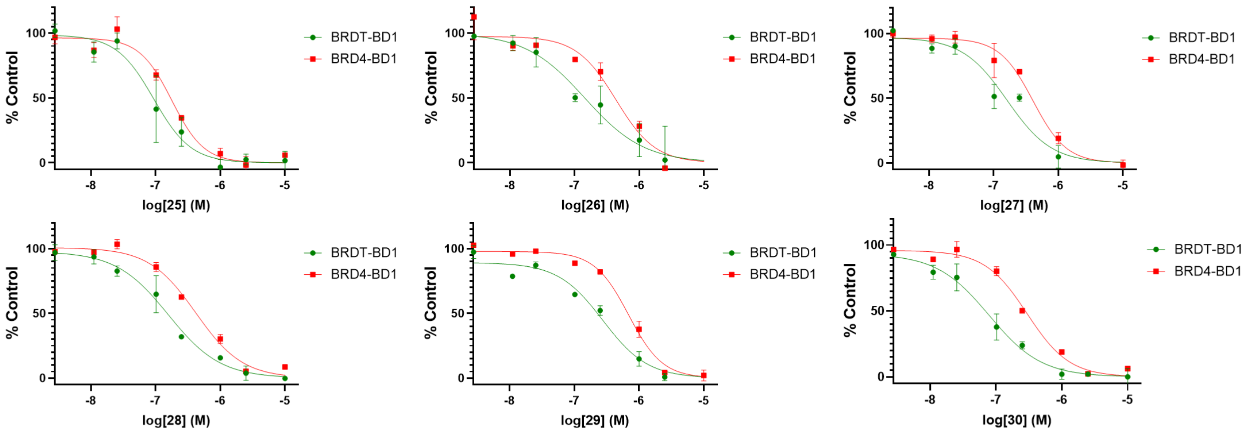

Figure S1. Continued. Dose-response curve of compound analogues binding to BRDT-BD1 and BRD4-BD1 by fluorescence polarization.

Table S1. AlphaScreen IC_{50} data and selectivity ratios for compounds **8-23**

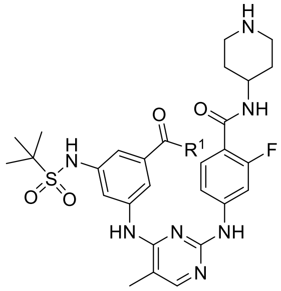

Compound	R[1]	AlphaScreen IC_{50} (nM)		SI
		BRDT-BD1	BRD4-BD1	
**JQ-1**	-	133	65.0	0.49
**8**	OMe	29.5	51.3	1.7
**9**	Gly	13.7	16.7	1.2
**10**	Phe	17.3	28.4	1.6
**11**	D-Phe	12.7	14.3	1.1
**12**	Thr	15.9	52.8	3.3
**13**	Ser	19.3	256	13
**14**	Glu	21.3	124	5.9
**15**	Gln	22.0	119	5.4
**16**	*tert*-Butylamine	24.5	43.7	1.3
**17**	Phenethylamine	39.3	92.0	2.3
**18**	$NHCH_2CO_2Me$	20.4	32.2	1.7

**19**	$NH(CH_2)_2CO_2H$	12.7	53.0	4.2
**20**	$NH(CH_2)_3CO_2H$	11.1	29.2	2.6
**21**	$NHCH_2SO_3H$	11.5	22.2	2.0
**22**	$NHCH_2CH_2SO_2NH_2$	9.2	16.9	1.8
**23**	$NHCH_2CONH_2$	12.2	12.2	1.0

AlphaScreen, single experiment performed in quadruplicate. The experiments were carried out as described before in: Jiang, J.; Sigua, L. H.; Chan, A.; Kalra, P.; Pomerantz, W. C. K.; Schönbrunn, E.; Qi, J.; Georg, G. I. Dihydropyridine lactam analogs targeting bet bromodomains. *ChemMedChem* **2022**, 17 (1), e202100407.

Table S2. AlphaScreen BRDT, BRD4 activity data for **SG3-179** and compounds **13** and **14**

	IC_{50} (nM)			
Compound	BRDT-BD1	BRDT-BD2 [a]	BRD4-BD1 [a]	BRD4-BD2
**SG3-179**	23	-	21	-
**13**	19	113	256	181
**14**	21	726	124	618

Single experiment performed in quadruplicate.

Table S3: Crystallographic data collection and refinement statistics

Protein		BRD4-BD1	BRDT-BD1
Compound		14	14
PDB ID		7MR8	9YCQ
		Data Collection	
Wavelength		0.9795	1.03319
Space group		P 21 21 21	P 21 21 21
Unit cell dimensions	a	38.54	36.67
	b	39.99	74.40
	c	96,33	82.25
	α	90	90
	β	90	90
	γ	90	90
Resolution range (Å)		30.77 - 1.20 (1.23 - 1.20)	41.12 - 1.40 (1.44 - 1.40)
Unique reflections		46061 (3099)	45152 (3289)
Rmeas		0.044 (0.186)	0.097 (1.227)
CC(1/2) (%)		99.9 (98.3)	99.7 (91.3)
Completeness (%)		97.1 (90.2)	100 (100)
I/σI		26.3 (9.8)	20.2 (7.4)
Wilson B ($Å^2$)		16.9	25.2
		Structure Refinement	
Rwork (%)		16.0 (16.3)	18.2 (22.5)
Rfree[a] (%)		17.4 (19.3)	20.4 (25.6)
Average B ($Å^2$)	all	18.0	22.0
	protein	15.3	20.9
	ligand	15.7	25.3
	water	31.0	30.6
rmsd[b] bond lengths (Å)		0.005	0.006
rmsd angles (deg)		0.95	1.09
Ramachandran	favored (%)	99.20	99.53
	allowed (%)	0.80	0.47
	outliers (%)	0.0	0.0

Values in paranthesis are for the highest resolution bins.

[a] Rfree is Rcryst calculated for randomly chosen unique reflections, which were excluded from the refinement.

[b] rmsd = root-mean-square deviation from ideal values.

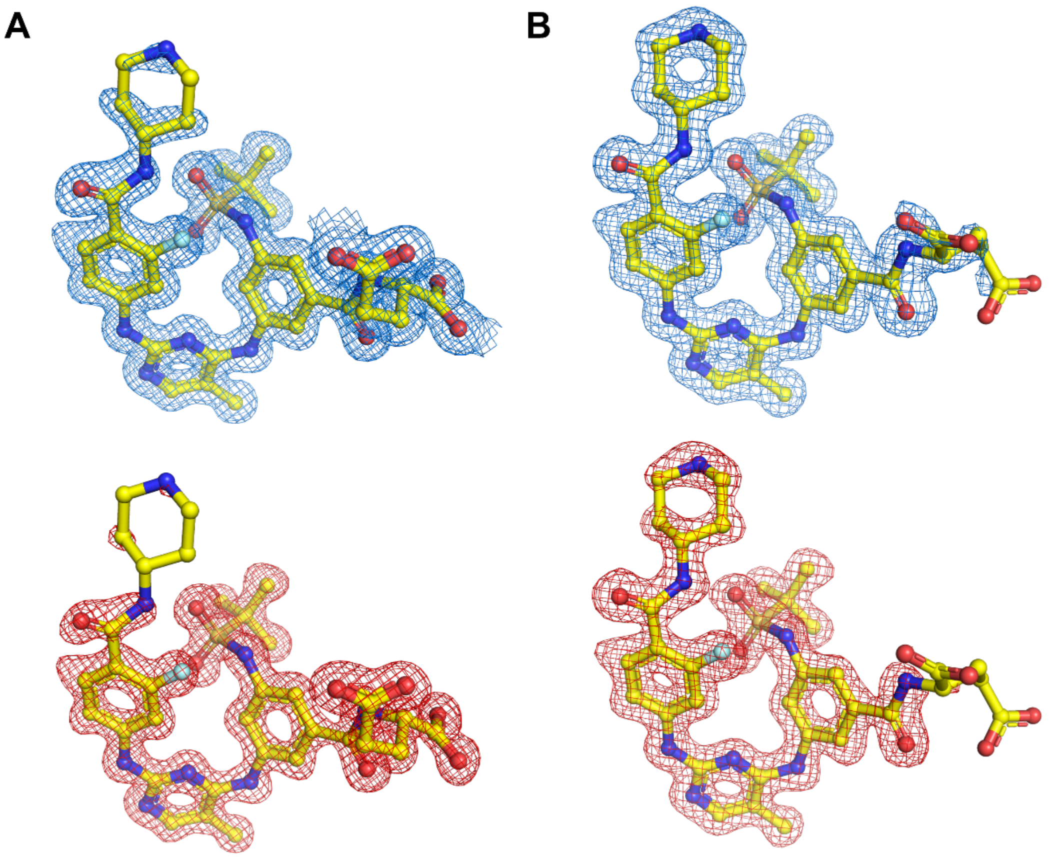

Figure S2. Electron density maps of compound 14 bound to BRD4 and BRDT. (A) **14** in BRD4-BD1 (PDB 7MR8), (B) **14** in BRDT-BD1 (PDB 9YCQ). 2Fo-Fc density maps upon refinement with ligand are shown in blue (contoured at 1σ), Fo-Fc density maps upon refinement omitting the ligand are shown in red (contoured at 3σ).

Table S4: Permeability Results of Test Compounds in MDCK-MDR1 Cells

Compound ID	BSA (%)	$P_{app\ (A\text{-}B)}$ (10^{-6}, cm/s)	$P_{app\ (B\text{-}A)}$ (10^{-6}, cm/s)	Efflux Ratio	Recovery (%)AP-BL	Recovery (%)BL-AP
Metoprolol	0	22.98	25.95	1.13	88.43	100.26
Prazosin	0	14.32	31.93	2.23	90.20	97.61
Imatinib	0	3.32	29.98	9.04	76.22	98.71
14	2	0.23	0.32	1.38	79.15	84.75
13**	2	<0.22	<0.065	N.A.	<82.55	<84.66

** The peak area of A-B and B-A receiver samples were set as 200 due to BLOD. Efflux Ratio was reported as N.A.

Table S5: The Assessment of MDCK-MDR1 Cell Monolayer Integrity

Compound ID	BSA (%)	TEER $_{A-B}$ ($\Omega \cdot cm^2$)	TEER $_{B-A}$ ($\Omega \cdot cm^2$)	LY Leakage $_{A-B}$ (%)	LY Leakage $_{B-A}$ (%)
Metoprolol	0	92	100	0.29	0.33
Prazosin	0	91	94	0.30	0.26
Imatinib	0	94	90	0.30	0.29
Compound 14	2	86	88	0.31	0.22
Compound 13	2	86	86	0.24	0.22

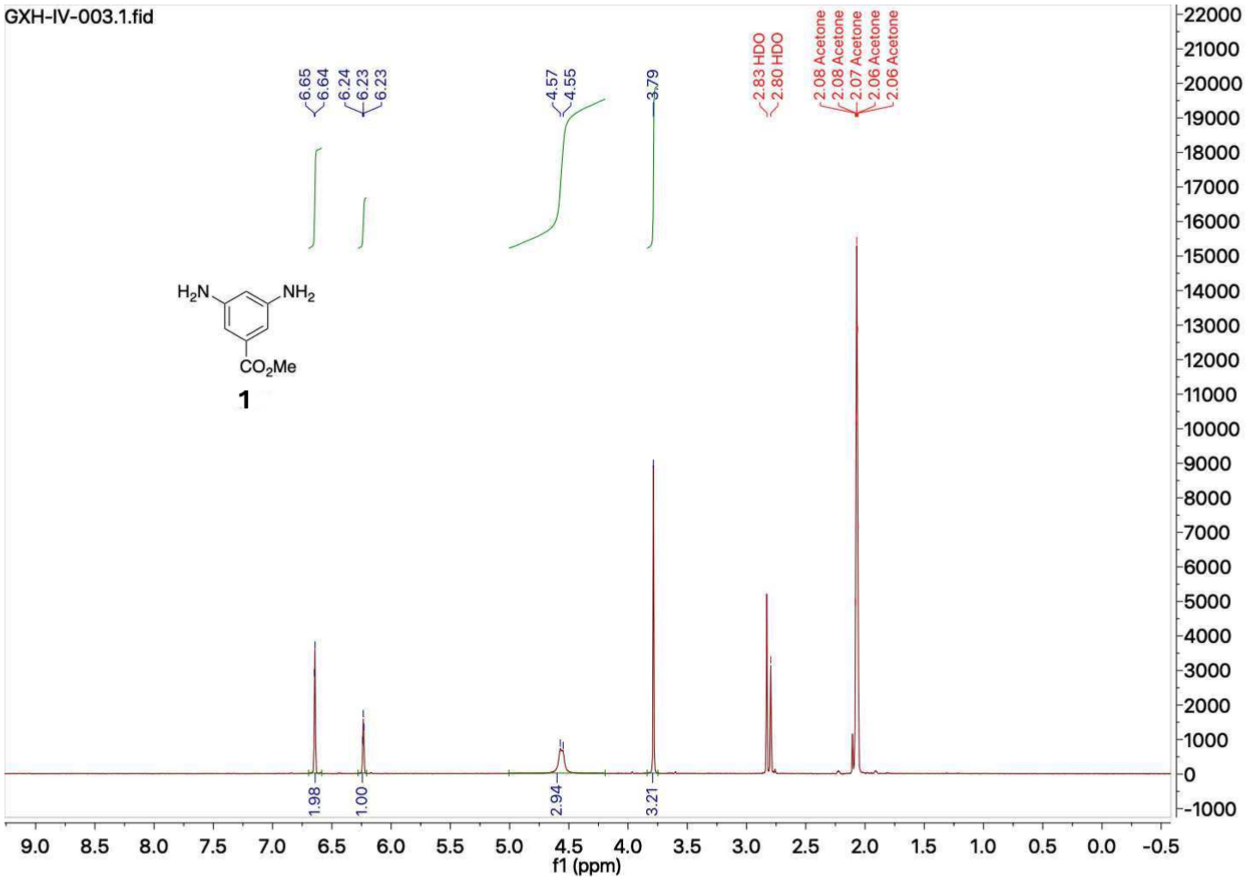
GXH-IV-003.1.fid
6.65
6.64
6.24
6.23
6.23
4.57
4.55
3.79
2.83 HDO
2.80 HDO
2.08 Acetone
2.08 Acetone
2.07 Acetone
2.06 Acetone
2.06 Acetone
H_2N
NH_2
CO_2Me
1
1.98
1.00
2.94
3.21
f1 (ppm)

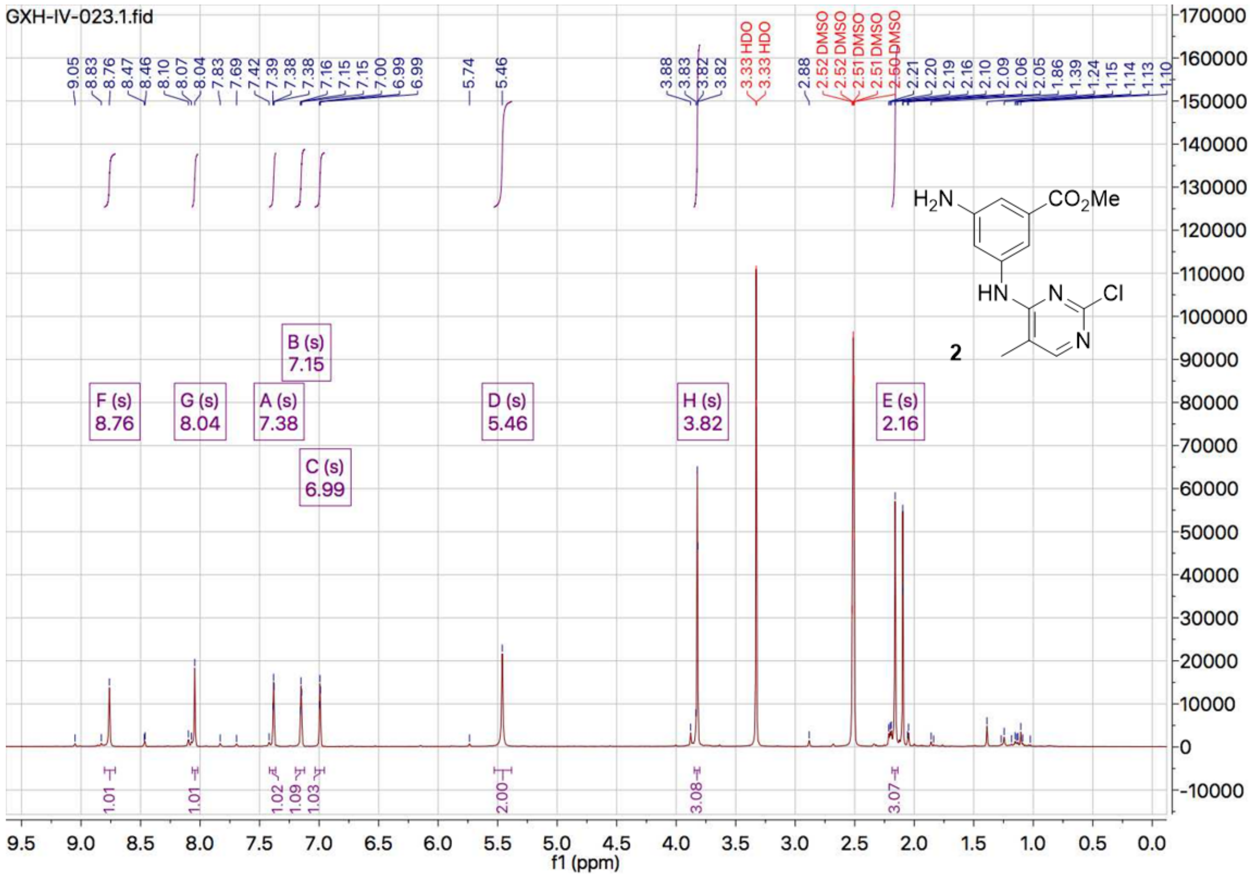
GXH-IV-023.1.fid
F (s)
8.76
G (s)
8.04
A (s)
7.38
B (s)
7.15
C (s)
6.99
D (s)
5.46
H (s)
3.82
E (s)
2.16
3.33 HDO
2.52 DMSO
2.51 DMSO
2.50 DMSO
2
f1 (ppm)

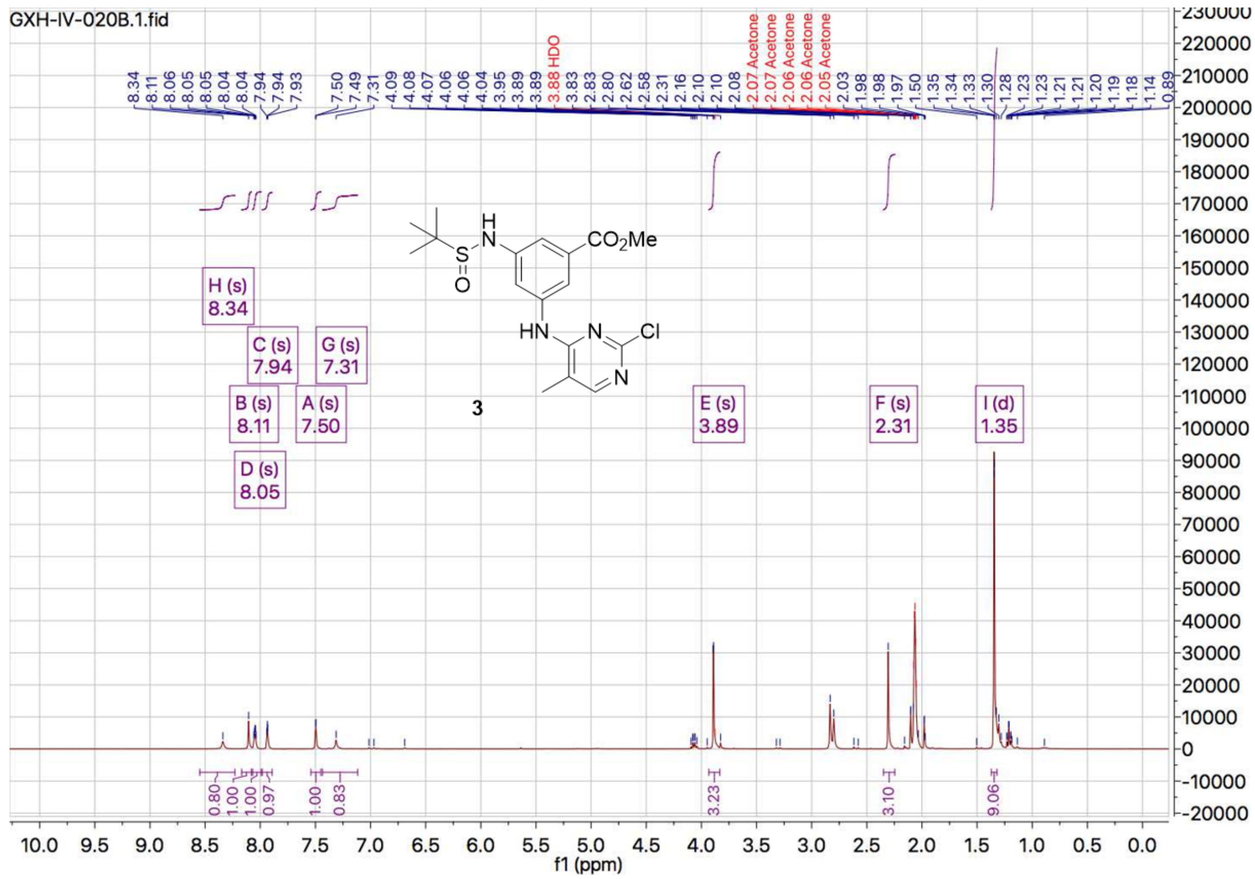
GXH-IV-020B.1.fid
H (s)
8.34
C (s)
7.94
G (s)
7.31
B (s)
8.11
A (s)
7.50
D (s)
8.05
E (s)
3.89
F (s)
2.31
I (d)
1.35
3
f1 (ppm)

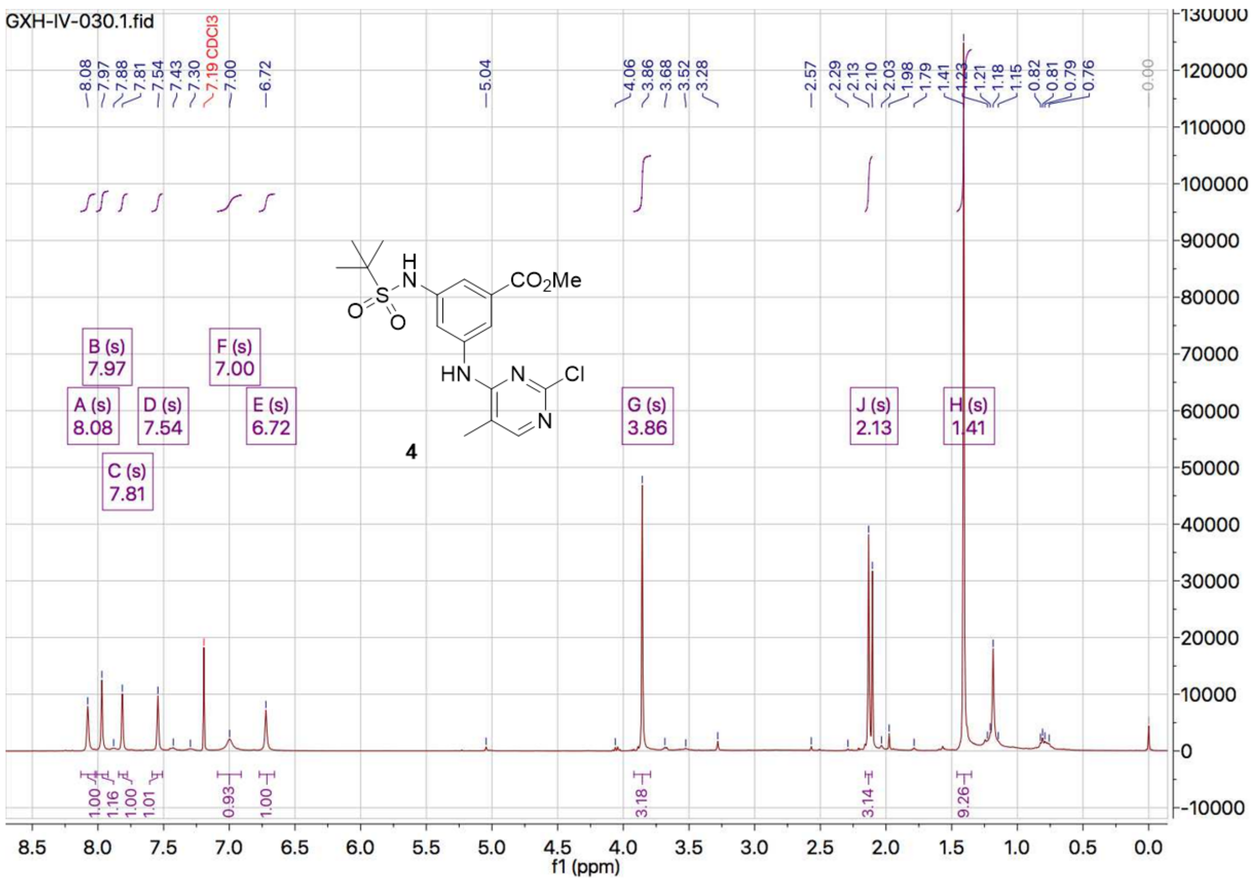
GXH-IV-030.1.fid
8.08
7.97
7.88
7.81
7.54
7.43
7.30
7.19 CDCl3
7.00
6.72
5.04
4.06
3.86
3.68
3.52
3.28
2.57
2.29
2.13
2.10
2.03
1.98
1.79
1.41
1.23
1.21
1.18
1.15
0.82
0.81
0.79
0.76
0.00
A (s) 8.08
B (s) 7.97
C (s) 7.81
D (s) 7.54
E (s) 6.72
F (s) 7.00
G (s) 3.86
J (s) 2.13
H (s) 1.41
H
N
CO2Me
S
O
O
HN
N
Cl
N
4
1.00
1.16
1.00
1.01
0.93
1.00
3.18
3.14
9.26
f1 (ppm)
130000
120000
110000
100000
90000
80000
70000
60000
50000
40000
30000
20000
10000
0
-10000

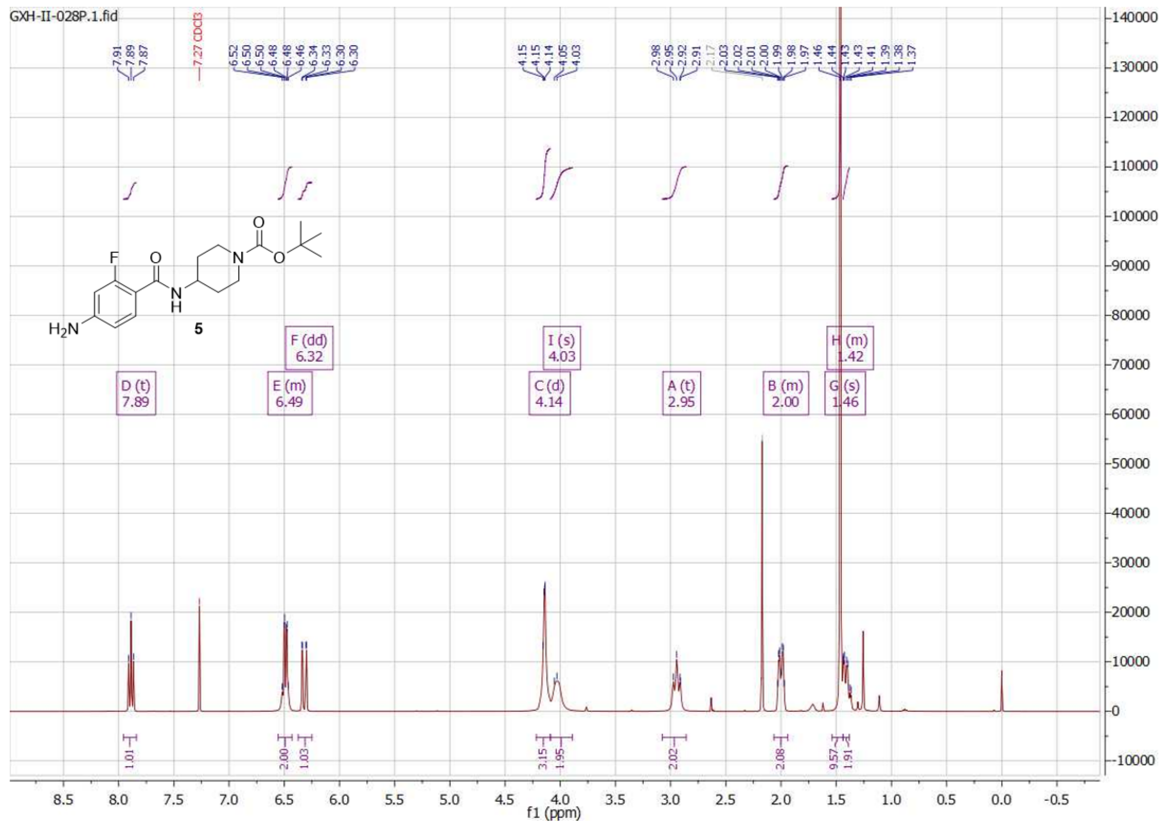
GXH-II-028P.1.fid
7.91
7.89
7.87
7.27 CDCl3
6.52
6.50
6.50
6.48
6.46
6.34
6.33
6.30
6.30
4.15
4.15
4.14
4.05
4.03
2.98
2.95
2.92
2.91
2.17
2.03
2.02
2.01
2.00
1.99
1.98
1.97
1.46
1.44
1.43
1.43
1.41
1.39
1.38
1.37
F
O
O
O
N
N
H
H_2N
5
F (dd)
6.32
I (s)
4.03
H (m)
1.42
D (t)
7.89
E (m)
6.49
C (d)
4.14
A (t)
2.95
B (m)
2.00
G (s)
1.46
1.01
2.00
1.03
3.15
1.95
2.02
2.08
9.57
1.91
f1 (ppm)

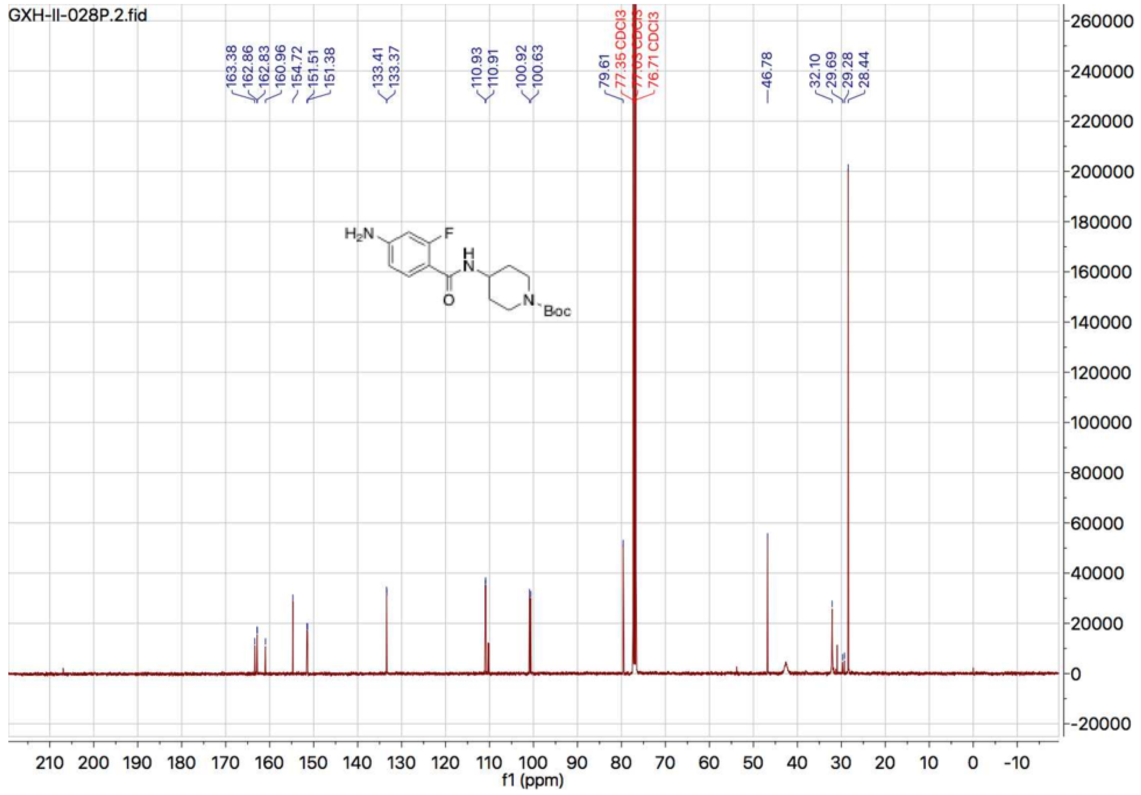
GXH-II-028P.2.fid
163.38
162.86
162.83
160.96
154.72
151.51
151.38
133.41
133.37
110.93
110.91
100.92
100.63
79.61
77.35 CDCl3
77.03 CDCl3
76.71 CDCl3
46.78
32.10
29.69
29.28
28.44
H_2N
F
H
N
O
N
Boc
f1 (ppm)

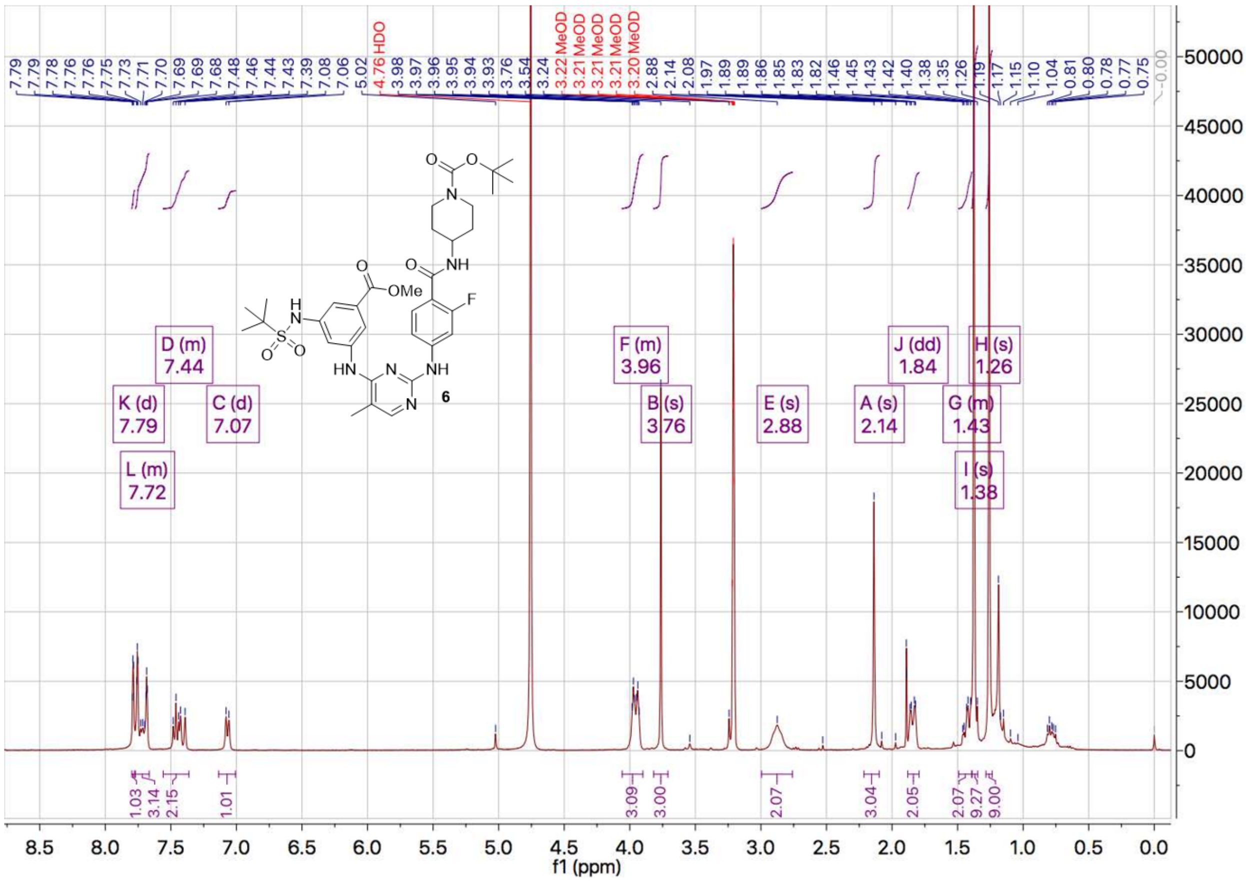
7.79 7.79 7.78 7.76 7.76 7.75 7.73 7.71 7.70 7.69 7.69 7.68 7.48 7.46 7.44 7.43 7.39 7.08 7.06 5.02
4.76 HDO
3.98 3.97 3.96 3.95 3.94 3.93 3.76 3.54 3.24
3.22 MeOD
3.21 MeOD
3.21 MeOD
3.21 MeOD
3.20 MeOD
2.88 2.14 2.08 1.97 1.89 1.86 1.85 1.83 1.82 1.46 1.45 1.43 1.42 1.40 1.38 1.35 1.26 1.19 1.17 1.15 1.10 1.04 0.81 0.80 0.78 0.77 0.75 -0.00
OMe
HN
NH
F
6
D (m) 7.44
K (d) 7.79
C (d) 7.07
L (m) 7.72
F (m) 3.96
B (s) 3.76
E (s) 2.88
A (s) 2.14
J (dd) 1.84
H (s) 1.26
G (m) 1.43
I (s) 1.38
1.03 3.14 2.15 1.01 3.09 3.00 2.07 3.04 2.05 2.07 9.27 9.00
50000 45000 40000 35000 30000 25000 20000 15000 10000 5000 0
8.5 8.0 7.5 7.0 6.5 6.0 5.5 5.0 4.5 4.0 3.5 3.0 2.5 2.0 1.5 1.0 0.5 0.0
f1 (ppm)

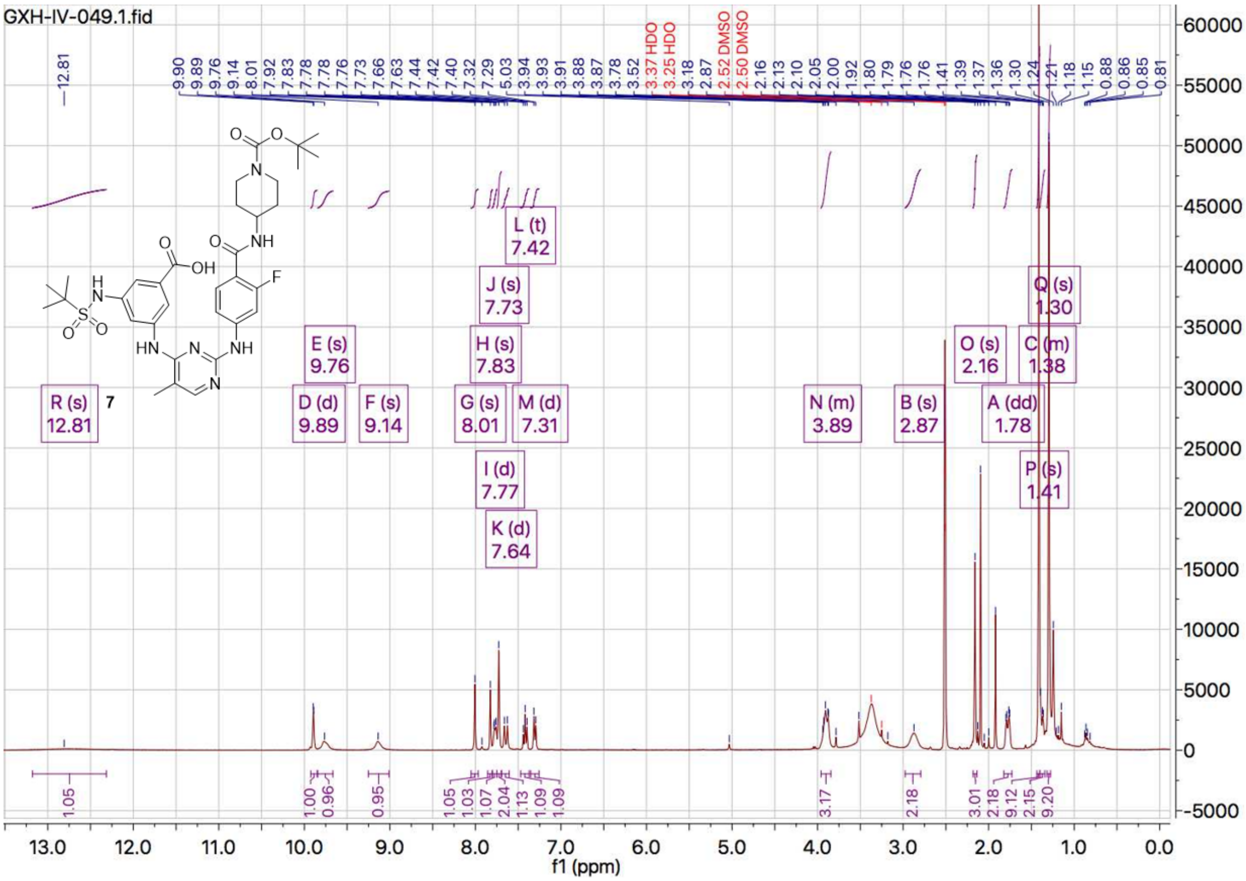
GXH-IV-049.1.fid
7
R (s) 12.81
E (s) 9.76
D (d) 9.89
F (s) 9.14
L (t) 7.42
J (s) 7.73
H (s) 7.83
G (s) 8.01
M (d) 7.31
I (d) 7.77
K (d) 7.64
N (m) 3.89
B (s) 2.87
O (s) 2.16
C (m) 1.38
A (dd) 1.78
Q (s) 1.30
P (s) 1.41
3.37 HDO
3.25 HDO
2.52 DMSO
2.50 DMSO
f1 (ppm)

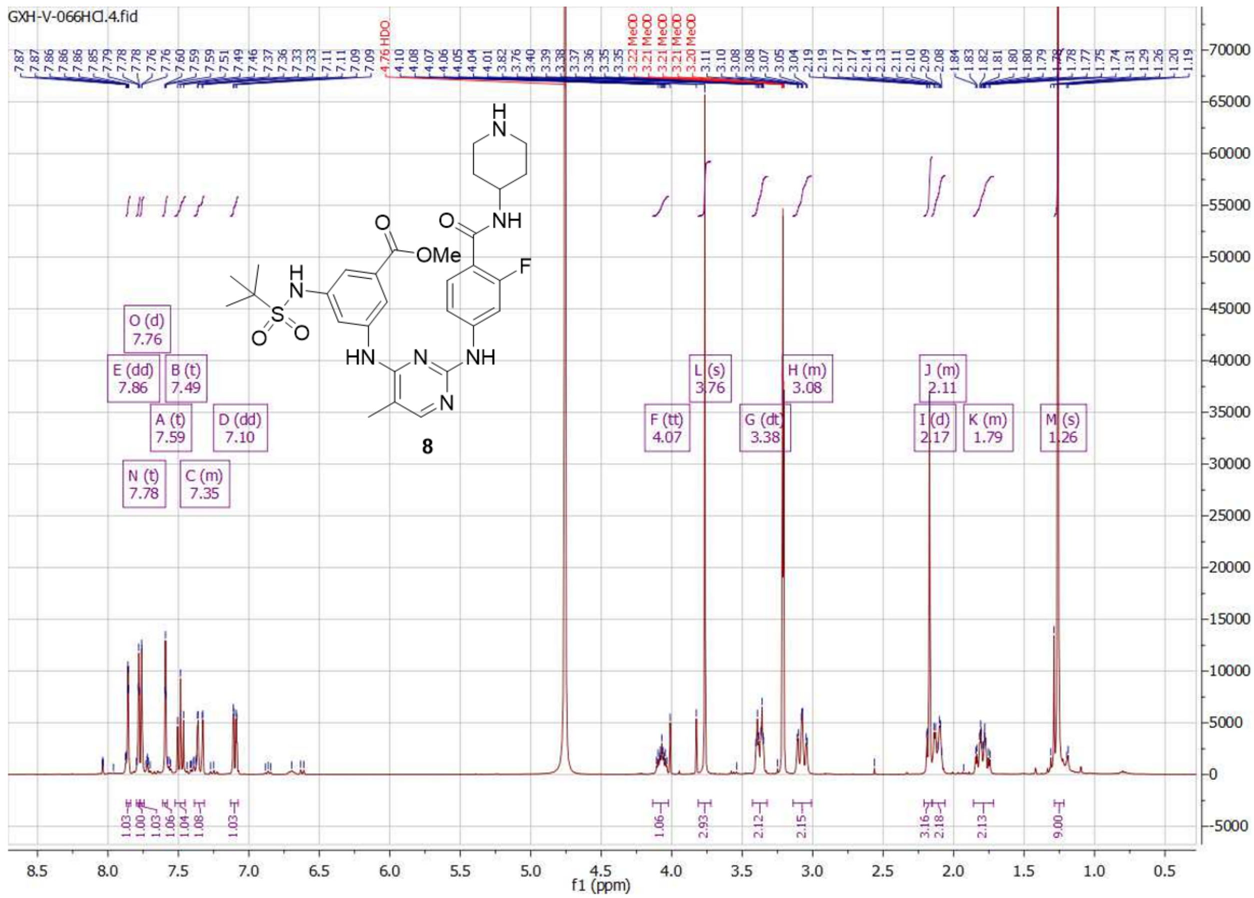
GXH-V-066HCl.4.fid
4.76 HDO
3.22 MeOD
3.21 MeOD
3.21 MeOD
3.21 MeOD
3.20 MeOD
O (d) 7.76
E (dd) 7.86
B (t) 7.49
A (t) 7.59
D (dd) 7.10
N (t) 7.78
C (m) 7.35
L (s) 3.76
H (m) 3.08
F (tt) 4.07
G (dt) 3.38
J (m) 2.11
I (d) 2.17
K (m) 1.79
M (s) 1.26
OMe
NH
HN
F
8
f1 (ppm)

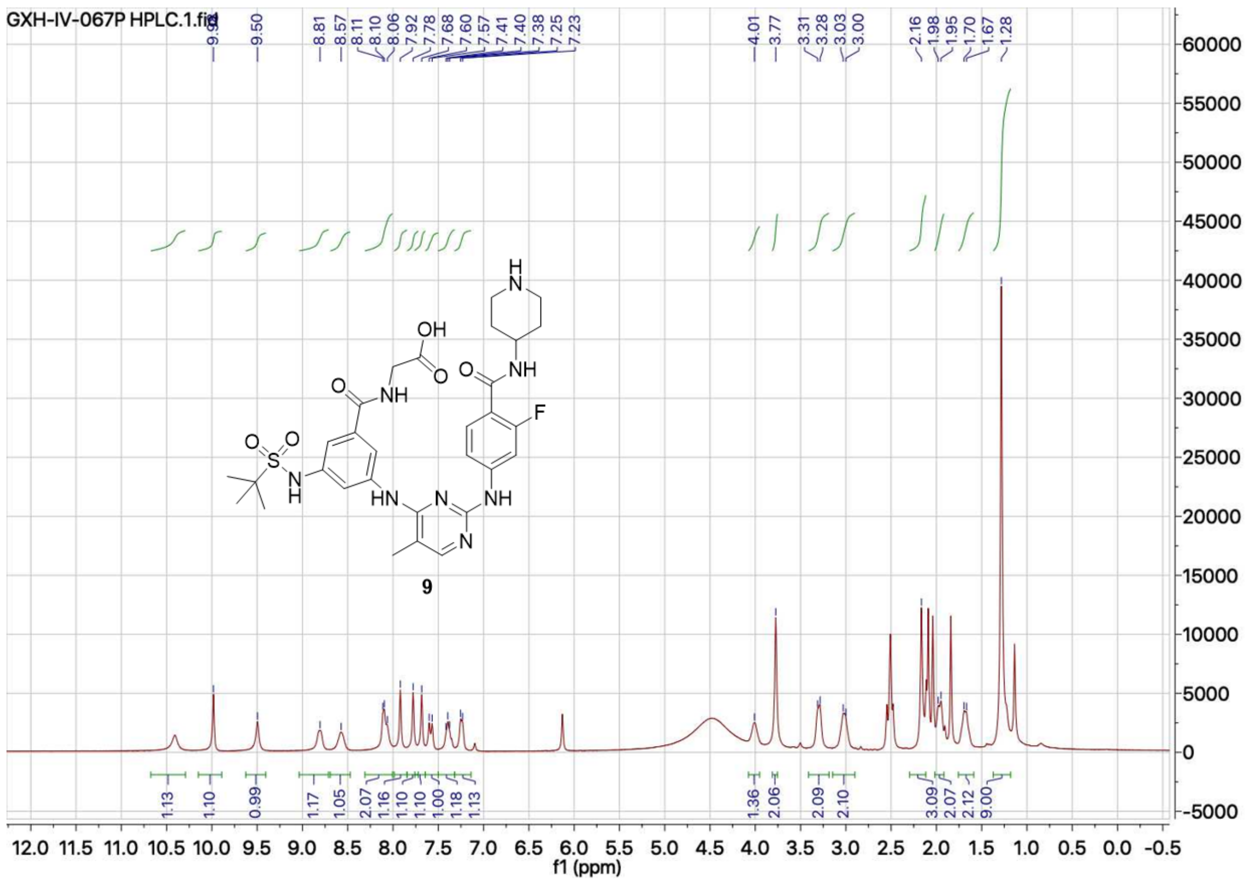
GXH-IV-067P HPLC.1.fid
9
f1 (ppm)

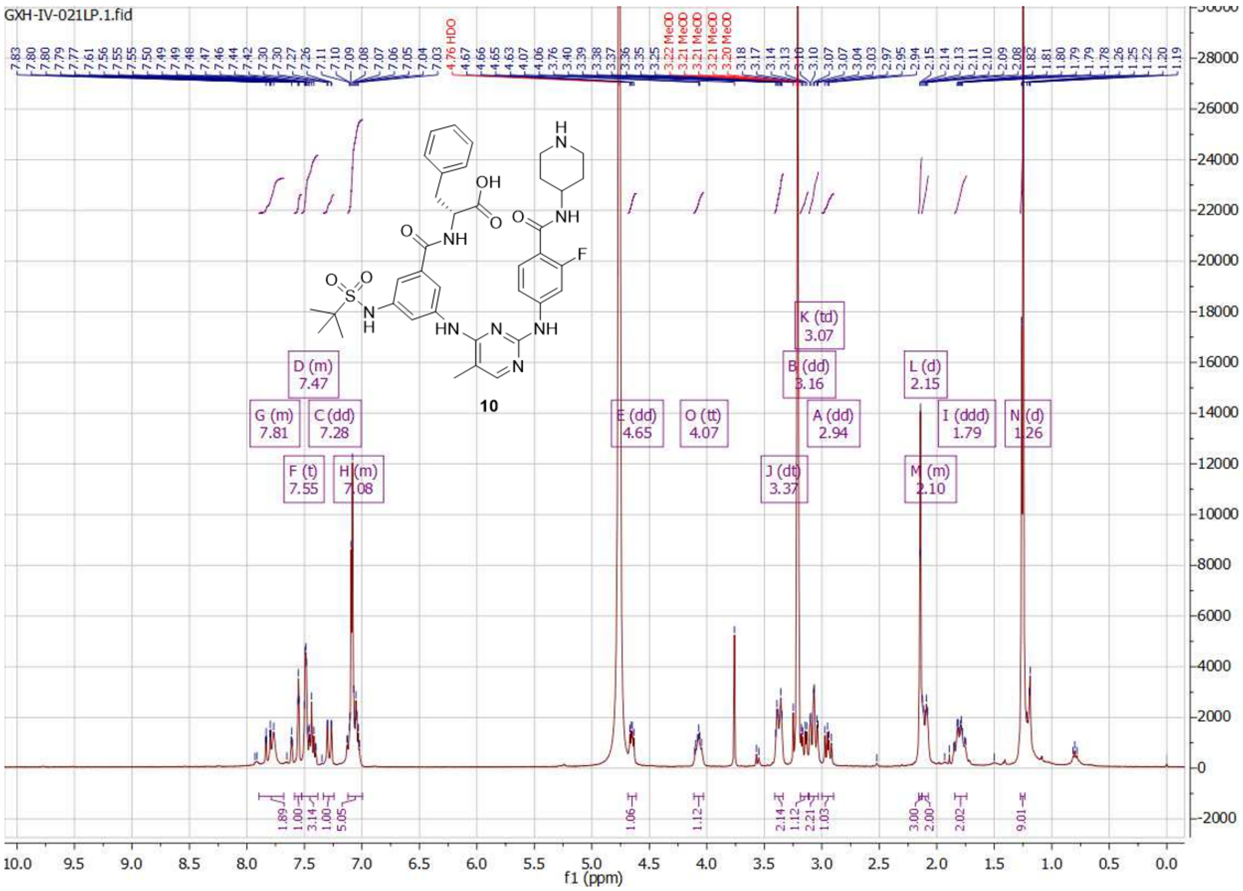

GXH-IV-021LP.1.fid
7.83 7.80 7.80 7.79 7.77 7.61 7.56 7.55 7.55 7.50 7.49 7.48 7.47 7.46 7.44 7.42 7.30 7.30 7.27 7.26 7.11 7.10 7.09 7.08 7.07 7.06 7.05 7.04 7.03
4.76 HDO
4.67 4.66 4.65 4.63 4.07 4.06 3.76 3.40 3.39 3.38 3.37 3.36 3.35 3.25
3.22 MeOD 3.21 MeOD 3.21 MeOD 3.21 MeOD 3.20 MeOD
3.18 3.17 3.14 3.13 3.10 3.10 3.07 3.07 3.04 3.03 2.97 2.95 2.94 2.15 2.14 2.13 2.11 2.10 2.09 2.08 1.82 1.81 1.80 1.79 1.78 1.26 1.25 1.22 1.20 1.19
OH
O
NH
O
O
NH
H
N
F
O
O
S
N
H
HN
N
NH
N
10
G (m) 7.81
D (m) 7.47
C (dd) 7.28
F (t) 7.55
H (m) 7.08
E (dd) 4.65
O (tt) 4.07
K (td) 3.07
B (dd) 3.16
A (dd) 2.94
J (dt) 3.37
L (d) 2.15
I (ddd) 1.79
M (m) 2.10
N (d) 1.26
1.89 1.00 3.14 1.00 5.05 1.06 1.12 2.14 1.12 2.21 1.03 3.00 2.00 2.02 9.01
10.0 9.5 9.0 8.5 8.0 7.5 7.0 6.5 6.0 5.5 5.0 4.5 4.0 3.5 3.0 2.5 2.0 1.5 1.0 0.5 0.0
f1 (ppm)
28000 26000 24000 22000 20000 18000 16000 14000 12000 10000 8000 6000 4000 2000 0 -2000

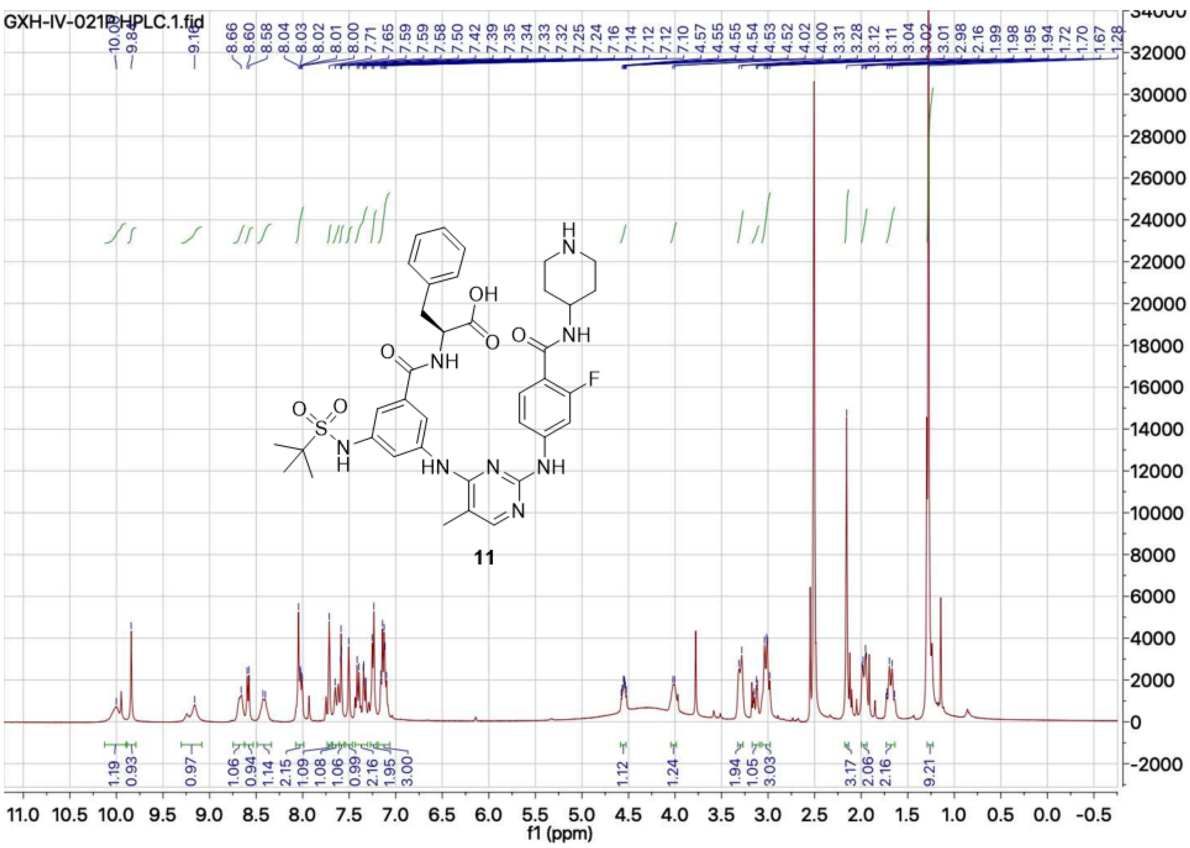
GXH-IV-021P HPLC.1.fid
11
f1 (ppm)

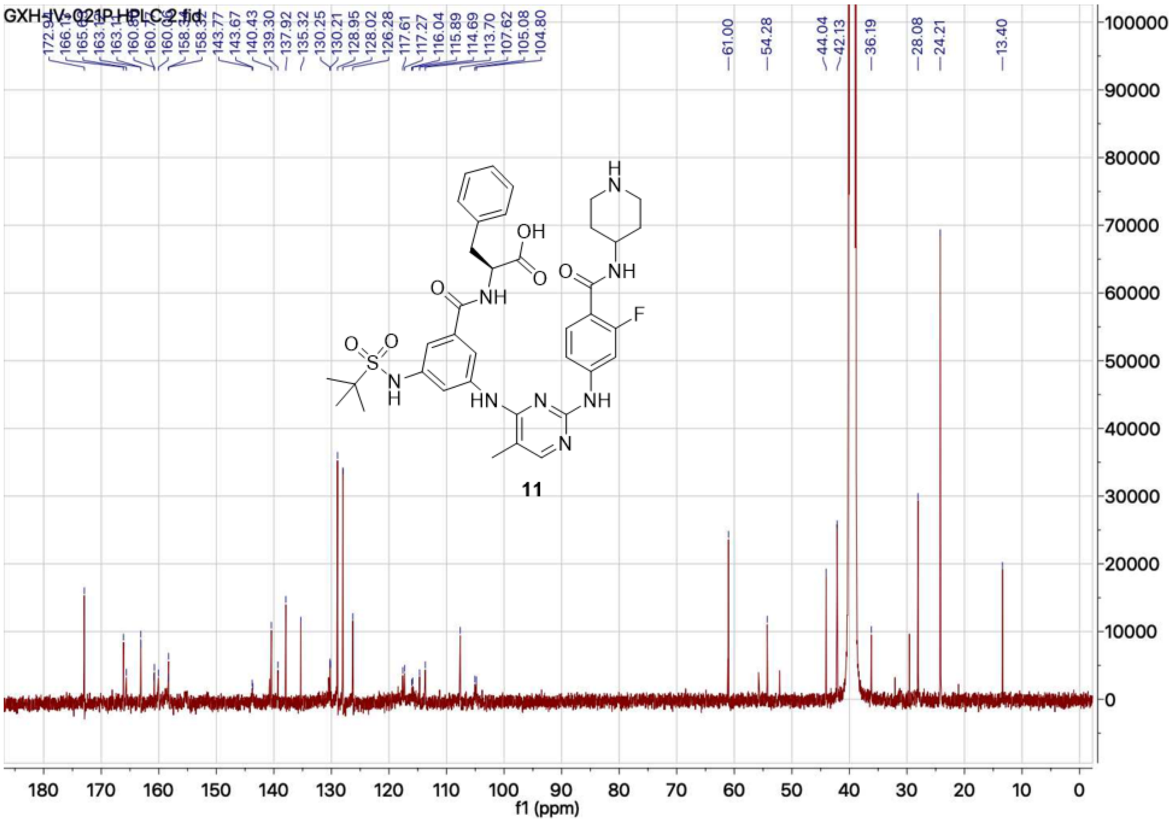
GXH-IV-021P HPLC.2.fid
11
f1 (ppm)

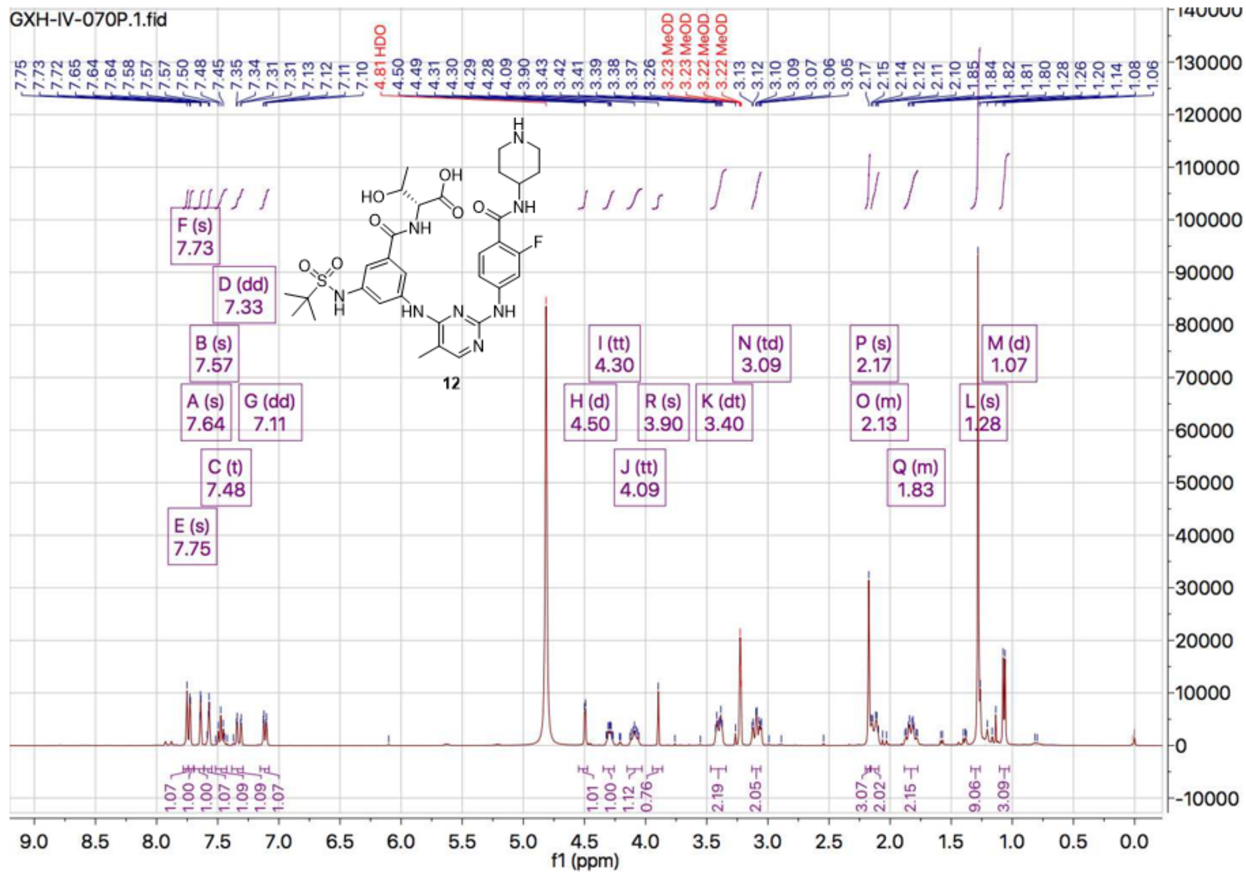

GXH-IV-070P.1.fid
7.75 7.73 7.72 7.65 7.64 7.64 7.58 7.57 7.57 7.50 7.48 7.45 7.35 7.34 7.31 7.31 7.13 7.12 7.11 7.10
4.81 HDO
4.50 4.49 4.31 4.30 4.29 4.28 4.09 3.90 3.43 3.42 3.41 3.39 3.38 3.37 3.26
3.23 MeOD 3.23 MeOD 3.22 MeOD 3.22 MeOD
3.13 3.12 3.09 3.07 3.06 3.05 2.17 2.15 2.14 2.12 2.11 2.10 1.85 1.84 1.82 1.81 1.80 1.28 1.26 1.20 1.14 1.08 1.06
F (s) 7.73
D (dd) 7.33
B (s) 7.57
A (s) 7.64
G (dd) 7.11
C (t) 7.48
E (s) 7.75
I (tt) 4.30
H (d) 4.50
R (s) 3.90
J (tt) 4.09
K (dt) 3.40
N (td) 3.09
P (s) 2.17
O (m) 2.13
Q (m) 1.83
M (d) 1.07
L (s) 1.28
12
1.07 1.00 1.00 1.07 1.09 1.09 1.07 1.01 1.00 1.12 0.76 2.19 2.05 3.07 2.02 2.15 9.06 3.09
f1 (ppm)

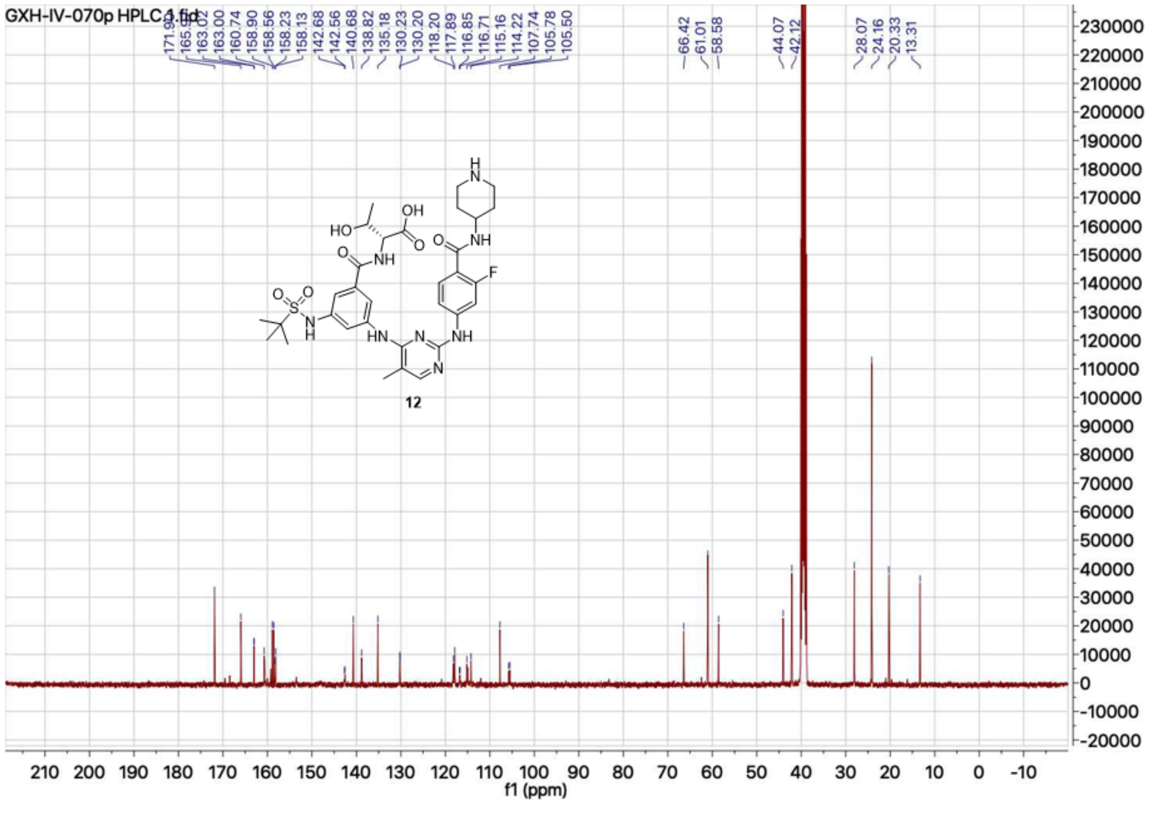

GXH-IV-070p HPLC1.fid
163.02 160.74 158.90 158.56 158.23 158.13 142.68 142.56 140.68 138.82 135.18 130.23 130.20 118.20 117.89 116.85 116.71 115.16 114.22 107.74 105.78 105.50
66.42 61.01 58.58 44.07 42.12 28.07 24.16 20.33 13.31
12
f1 (ppm)

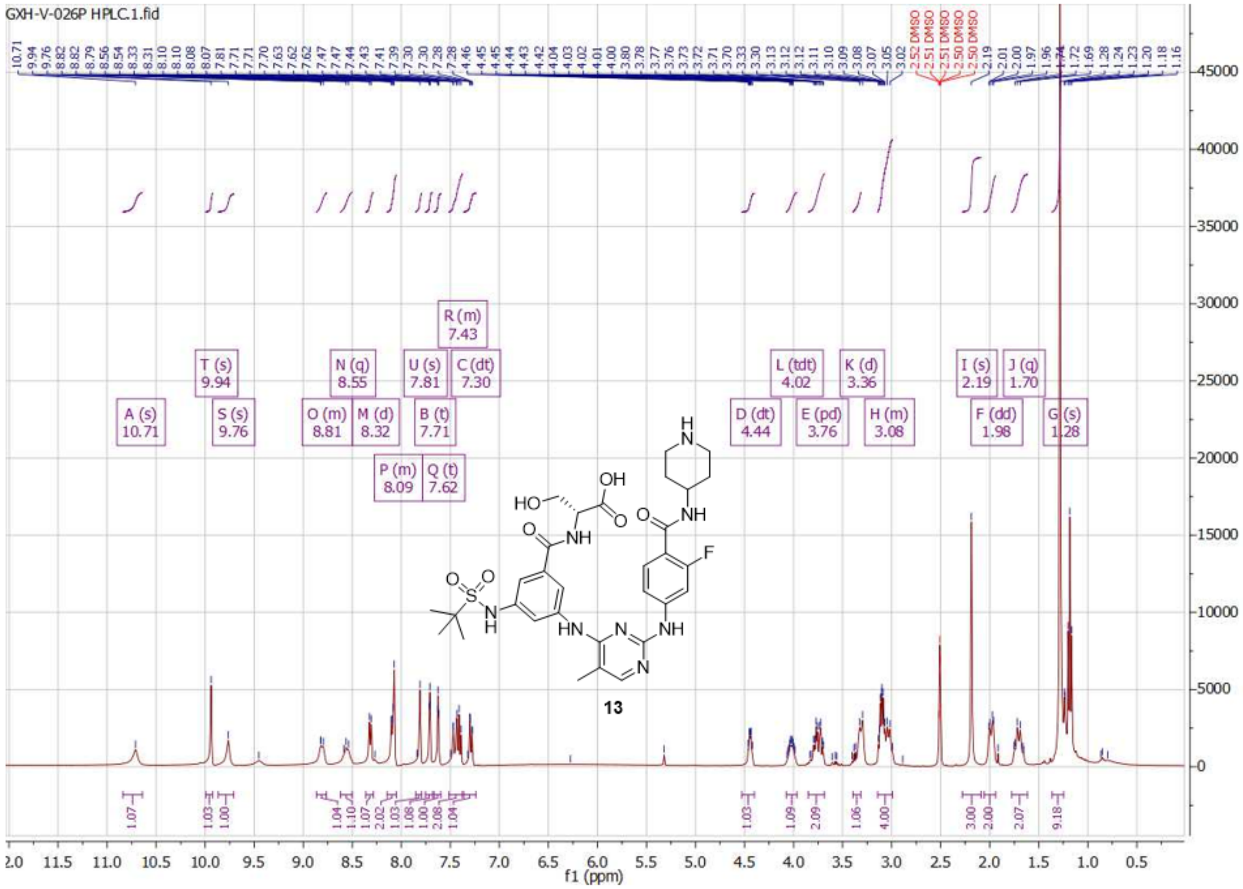
GXH-V-026P HPLC.1.fid
A (s) 10.71
T (s) 9.94
S (s) 9.76
O (m) 8.81
N (q) 8.55
M (d) 8.32
P (m) 8.09
U (s) 7.81
B (t) 7.71
Q (t) 7.62
R (m) 7.43
C (dt) 7.30
D (dt) 4.44
L (tdt) 4.02
E (pd) 3.76
K (d) 3.36
H (m) 3.08
I (s) 2.19
F (dd) 1.98
J (q) 1.70
G (s) 1.28
13
f1 (ppm)

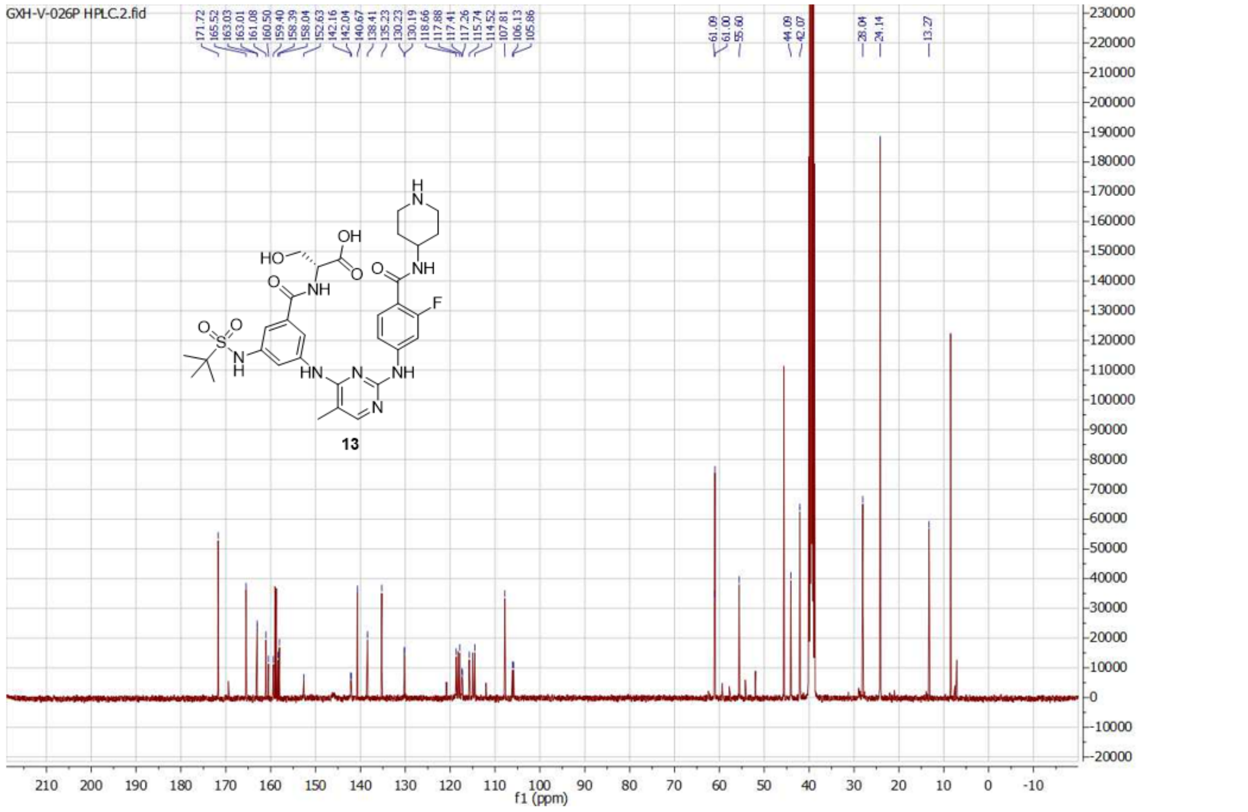
GXH-V-026P HPLC.2.fid
13
f1 (ppm)

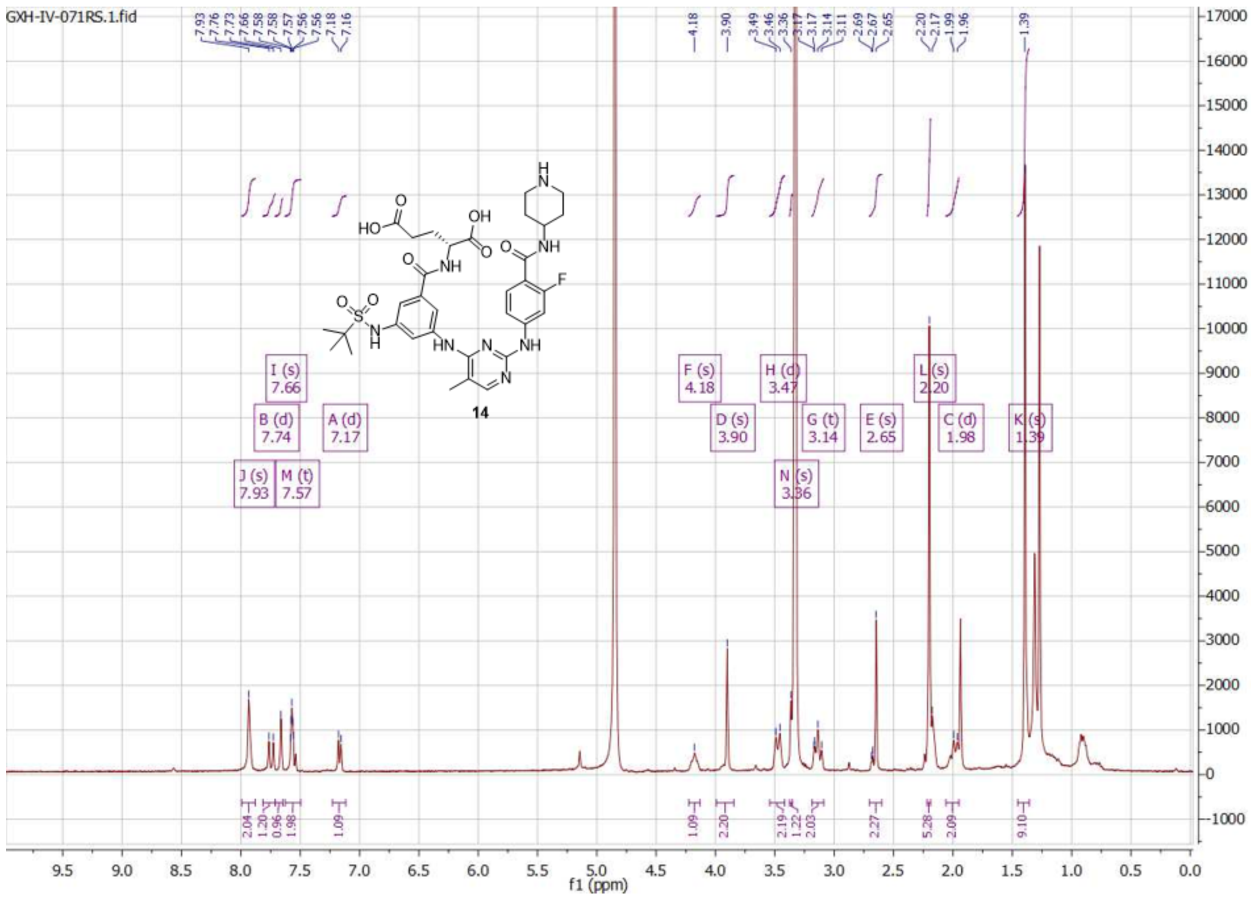

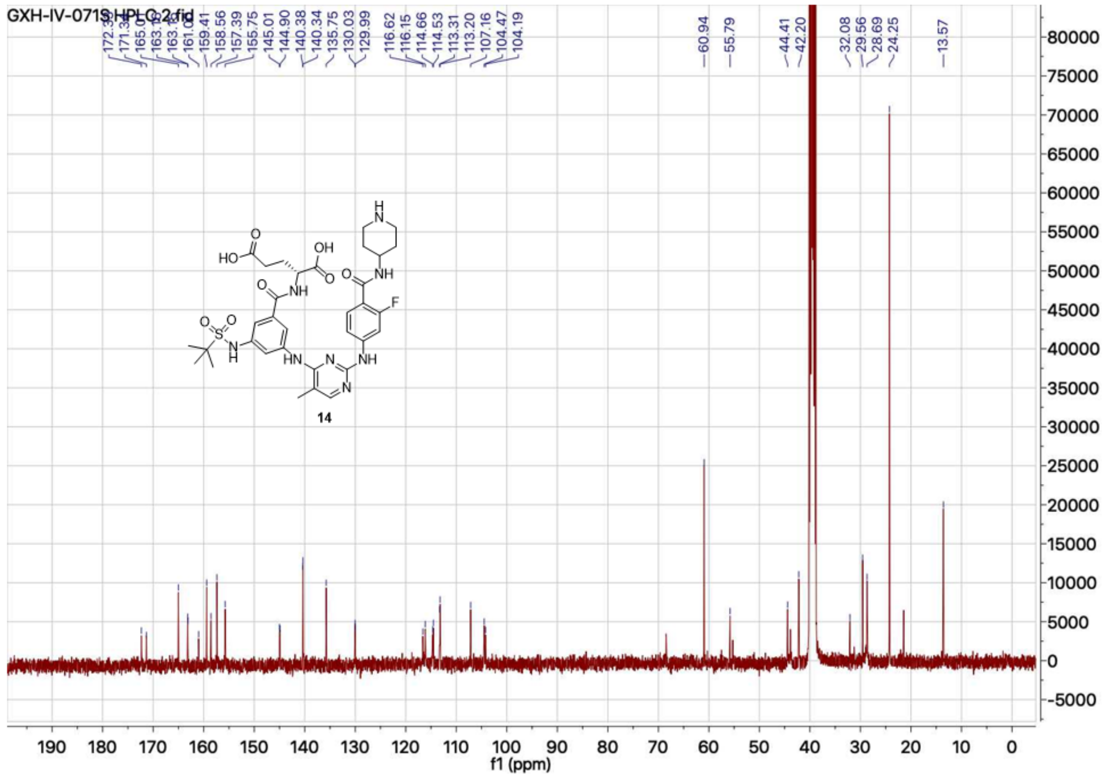

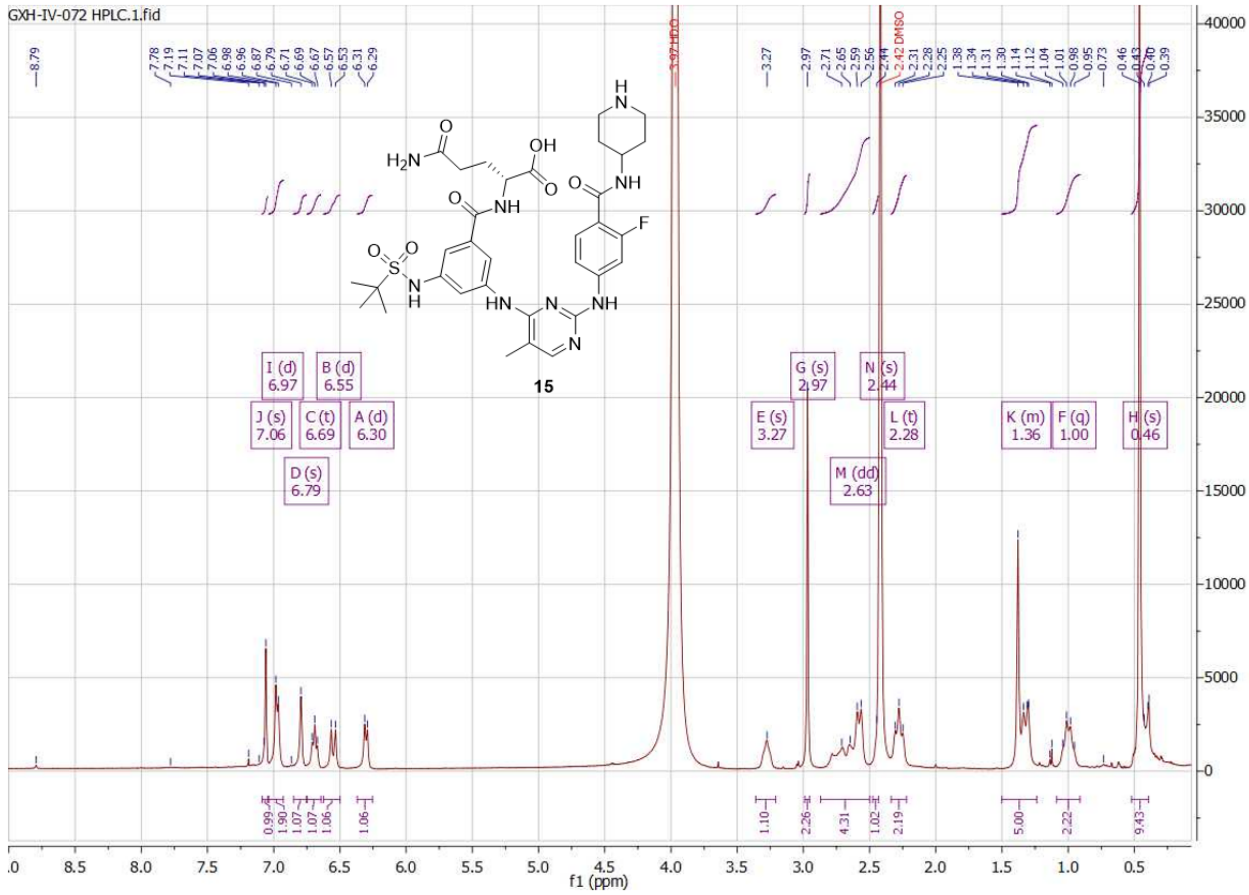
GXH-IV-072 HPLC.1.fid
15
I (d) 6.97
B (d) 6.55
J (s) 7.06
C (t) 6.69
A (d) 6.30
D (s) 6.79
G (s) 2.97
N (s) 2.44
E (s) 3.27
L (t) 2.28
M (dd) 2.63
K (m) 1.36
F (q) 1.00
H (s) 0.46
3.97 HDO
2.42 DMSO
f1 (ppm)

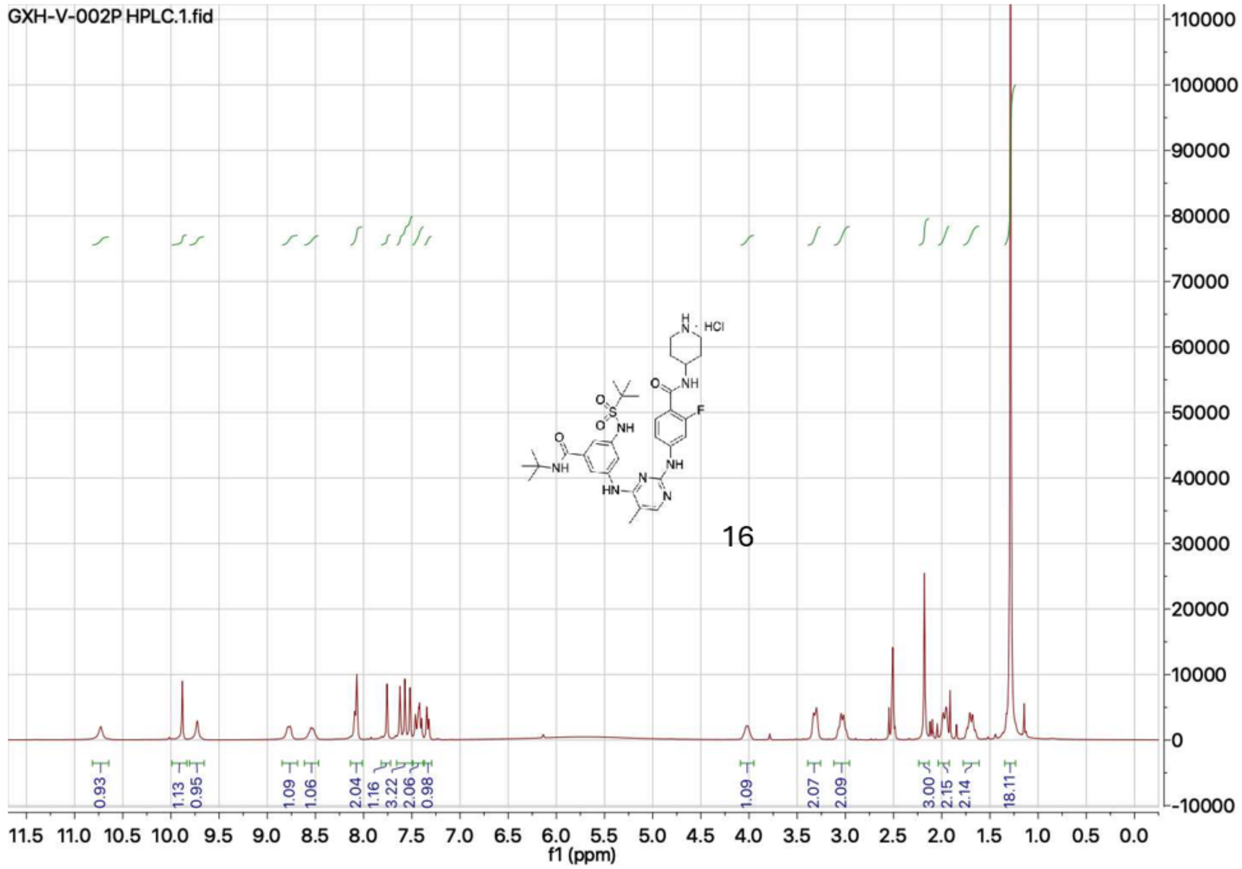
GXH-V-002P HPLC.1.fid
HCl
16
0.93
1.13
0.95
1.09
1.06
2.04
1.16
3.22
2.06
0.98
1.09
2.07
2.09
3.00
2.15
2.14
18.11
f1 (ppm)

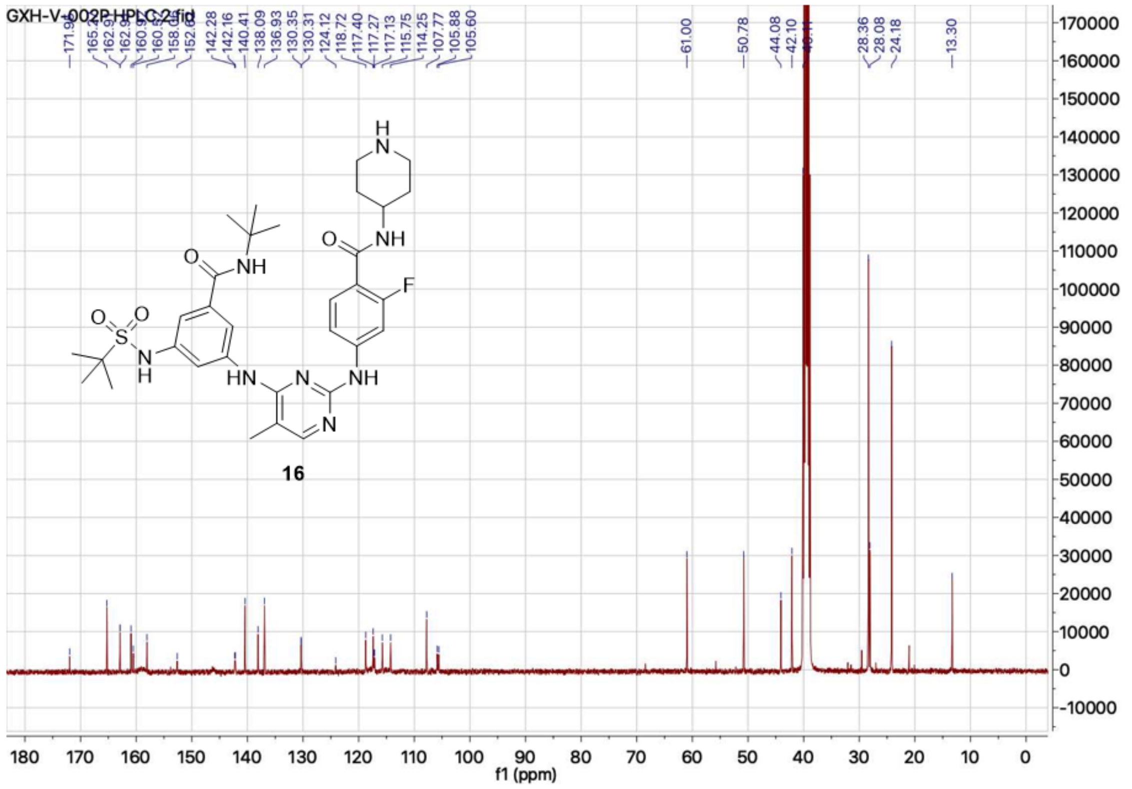
142.28
142.16
140.41
138.09
136.93
130.35
130.31
124.12
118.72
117.40
117.27
117.13
115.75
114.25
107.77
105.88
105.60
61.00
50.78
44.08
42.10
28.36
28.08
24.18
13.30
16
f1 (ppm)

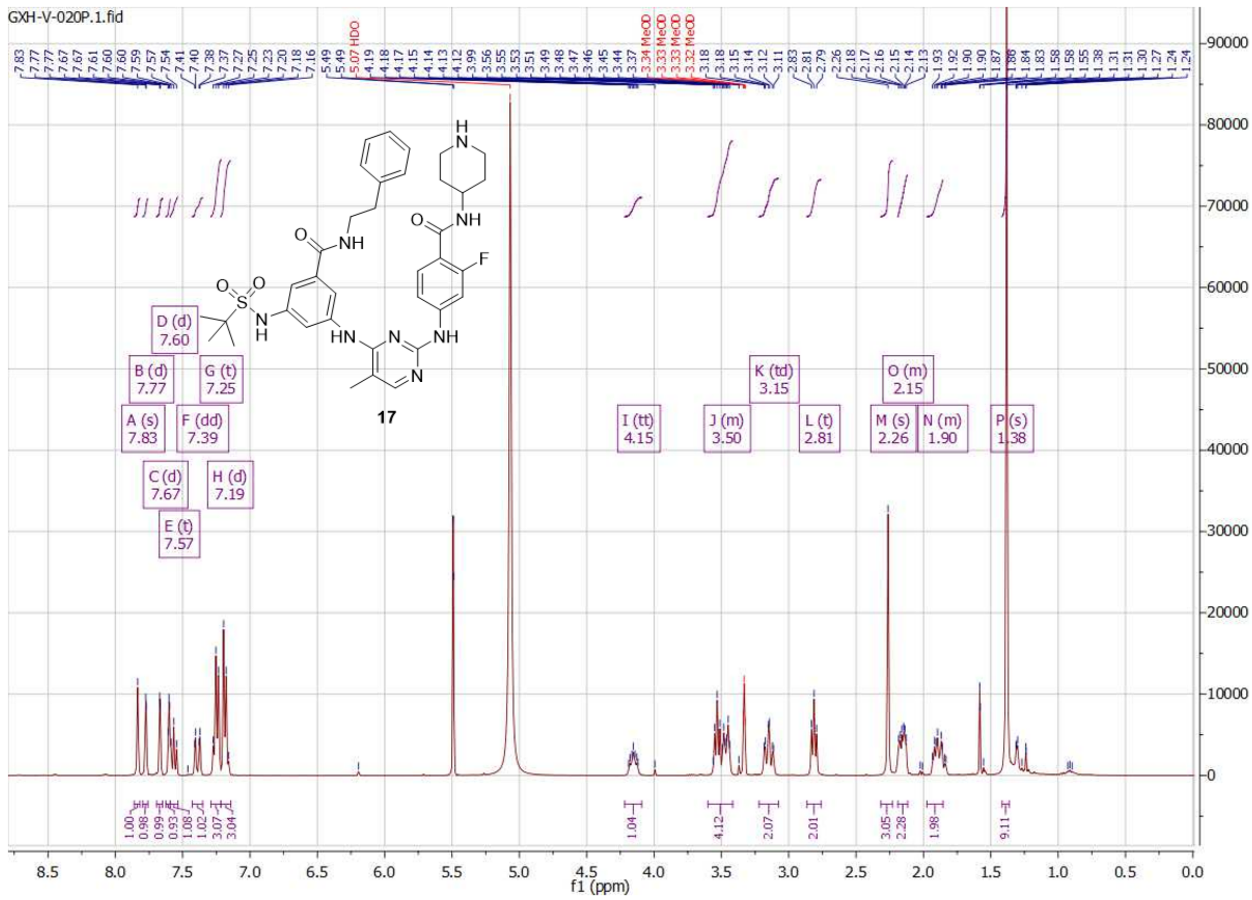
GXH-V-020P.1.fid
17
A (s) 7.83
B (d) 7.77
C (d) 7.67
D (d) 7.60
E (t) 7.57
F (dd) 7.39
G (t) 7.25
H (d) 7.19
I (tt) 4.15
J (m) 3.50
K (td) 3.15
L (t) 2.81
M (s) 2.26
O (m) 2.15
N (m) 1.90
P (s) 1.38
f1 (ppm)

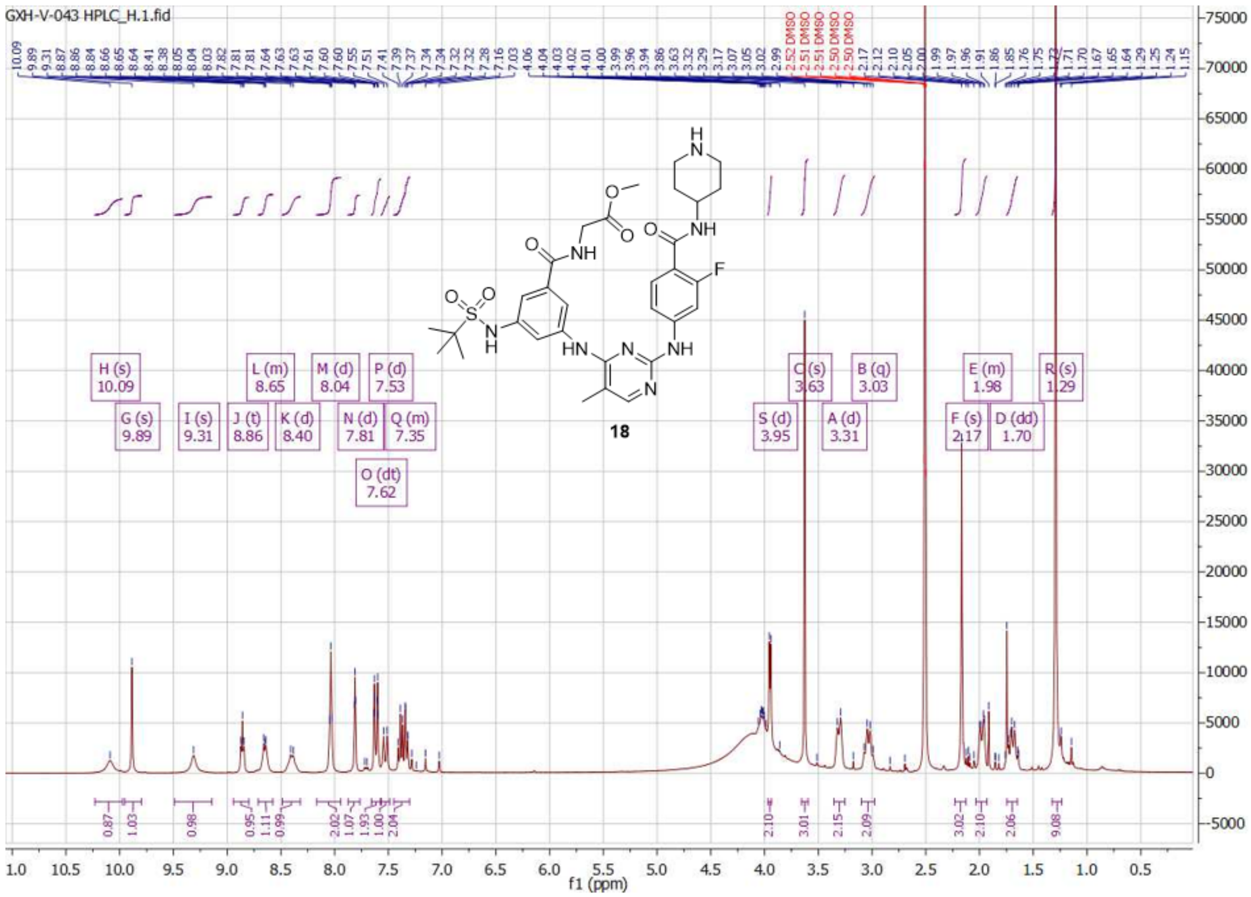

18

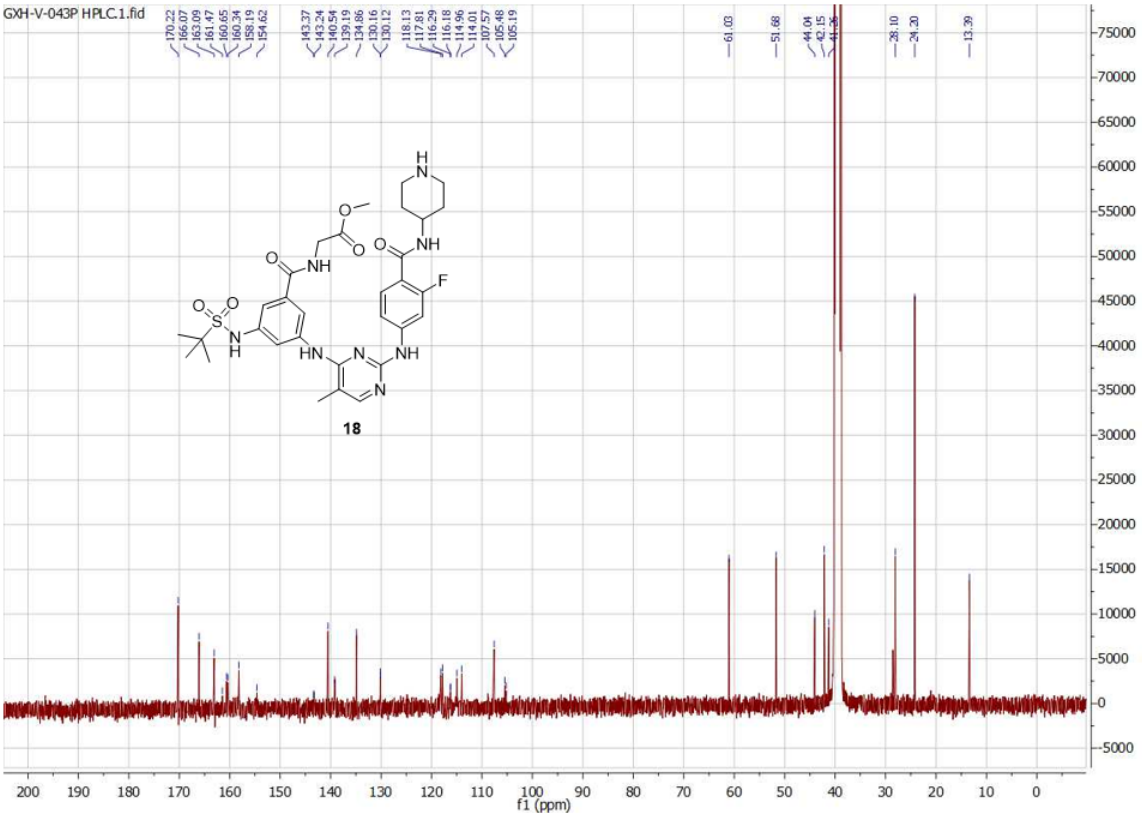

18

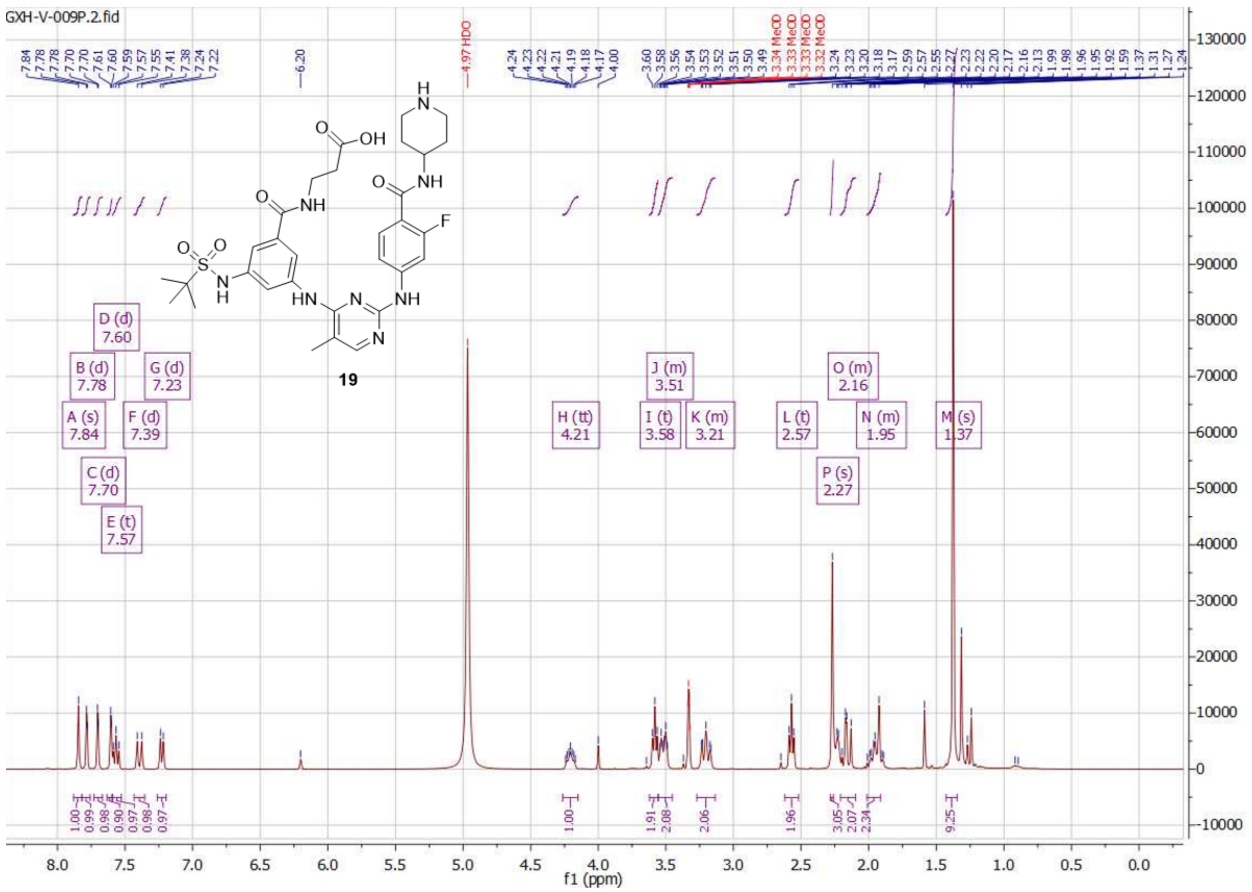

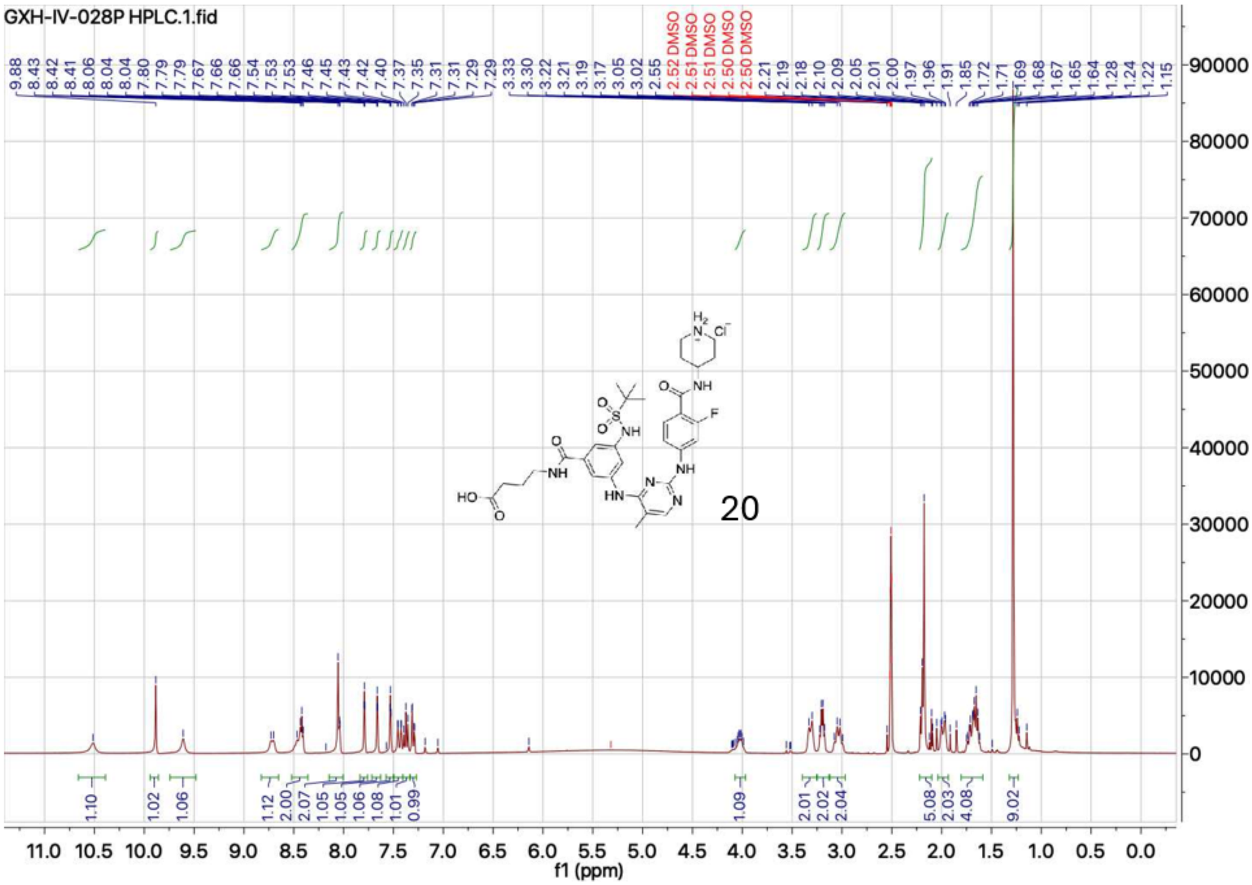
GXH-IV-028P HPLC.1.fid
9.88 8.43 8.42 8.41 8.06 8.04 8.04 7.80 7.79 7.79 7.67 7.66 7.66 7.54 7.53 7.53 7.46 7.45 7.43 7.42 7.40 7.37 7.35 7.31 7.31 7.29 7.29 3.33 3.30 3.22 3.21 3.19 3.17 3.05 3.02 2.55
2.52 DMSO
2.51 DMSO
2.51 DMSO
2.50 DMSO
2.50 DMSO
2.21 2.19 2.18 2.10 2.09 2.05 2.01 2.00 1.97 1.96 1.91 1.85 1.72 1.71 1.69 1.68 1.67 1.65 1.64 1.28 1.24 1.22 1.15
1.10 1.02 1.06 1.12 2.00 2.07 1.05 1.05 1.06 1.08 1.01 0.99 1.09 2.01 2.02 2.04 5.08 2.03 4.08 9.02
20
f1 (ppm)

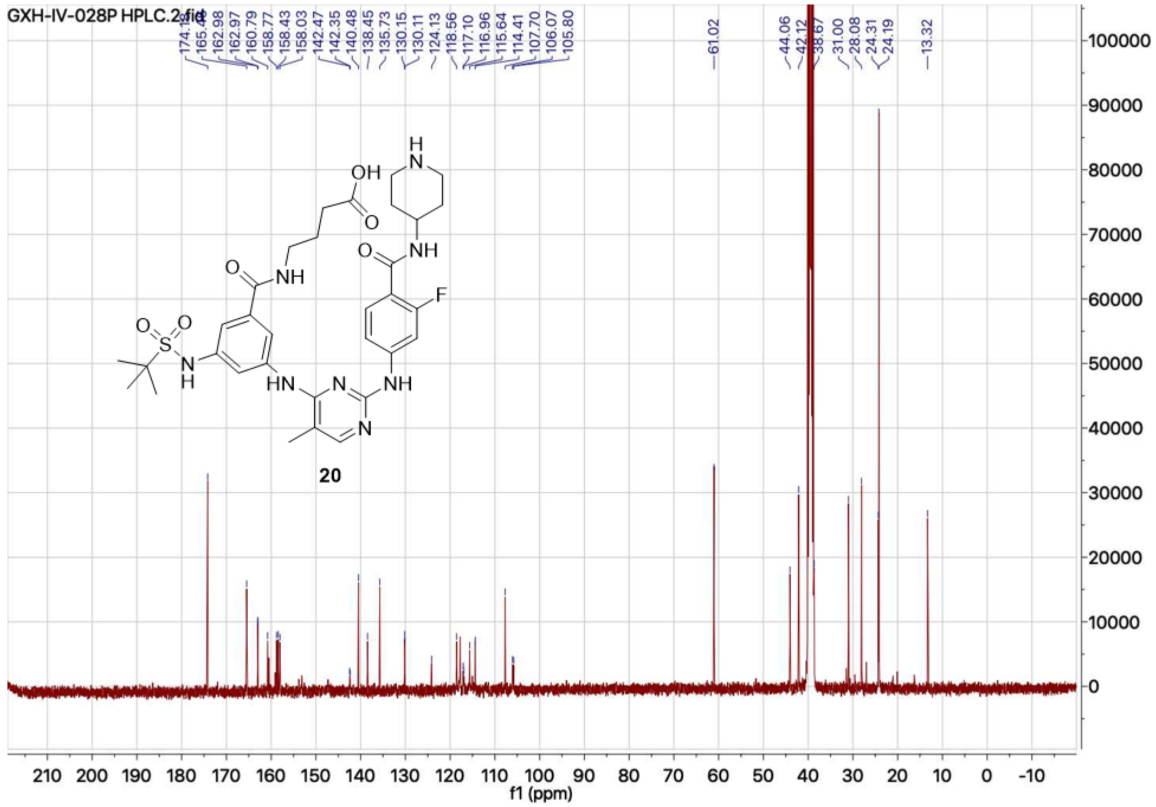
GXH-IV-028P HPLC.2.fid
174.19 165.38 162.98 162.97 160.79 158.77 158.43 158.03 142.47 142.35 140.48 138.45 135.73 130.15 130.11 124.13 118.56 117.10 116.96 115.64 114.41 107.70 106.07 105.80
61.02
44.06 42.12 38.67 31.00 28.08 24.31 24.19
13.32
20
f1 (ppm)

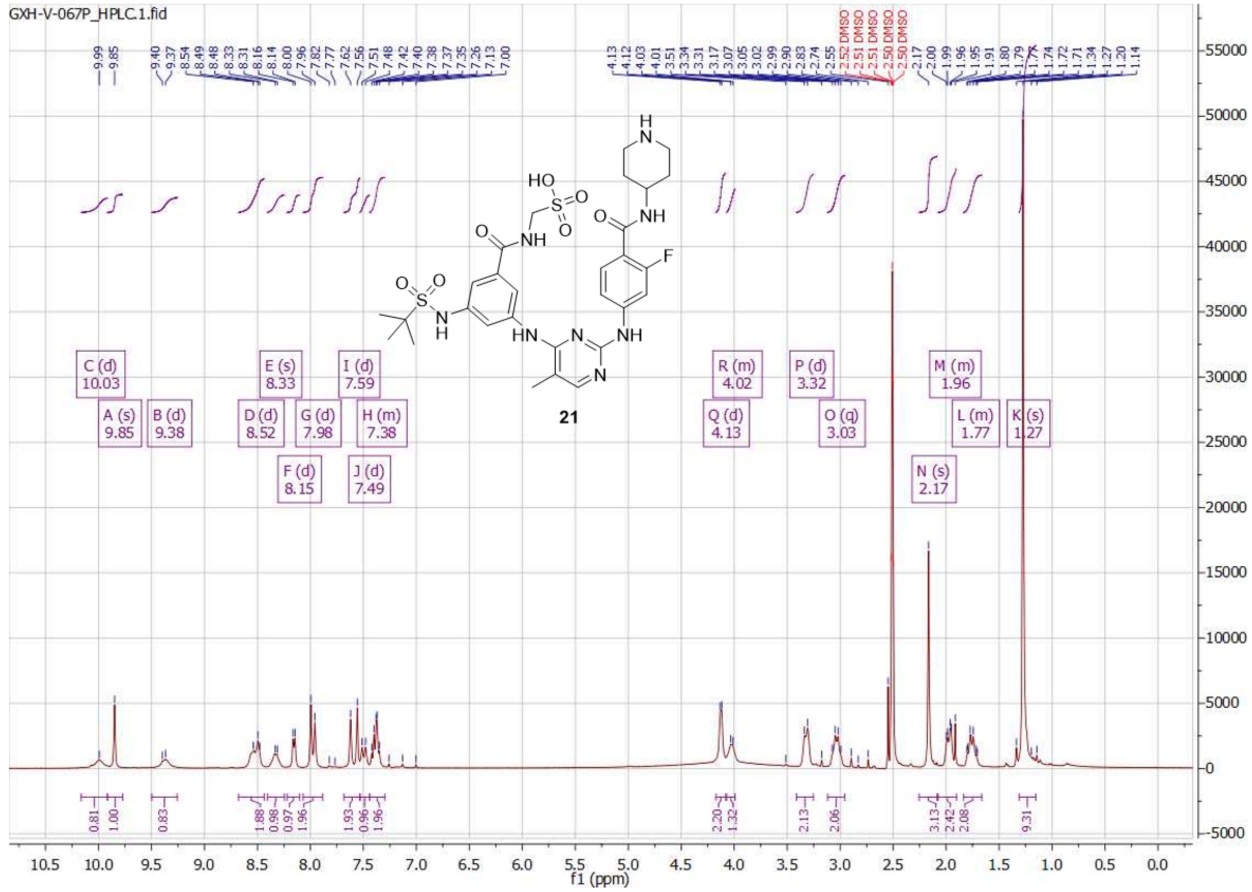
GXH-V-067P_HPLC.1.fid
C (d) 10.03
A (s) 9.85
B (d) 9.38
E (s) 8.33
D (d) 8.52
F (d) 8.15
G (d) 7.98
I (d) 7.59
H (m) 7.38
J (d) 7.49
R (m) 4.02
Q (d) 4.13
P (d) 3.32
O (q) 3.03
M (m) 1.96
L (m) 1.77
K (s) 1.27
N (s) 2.17
21
f1 (ppm)

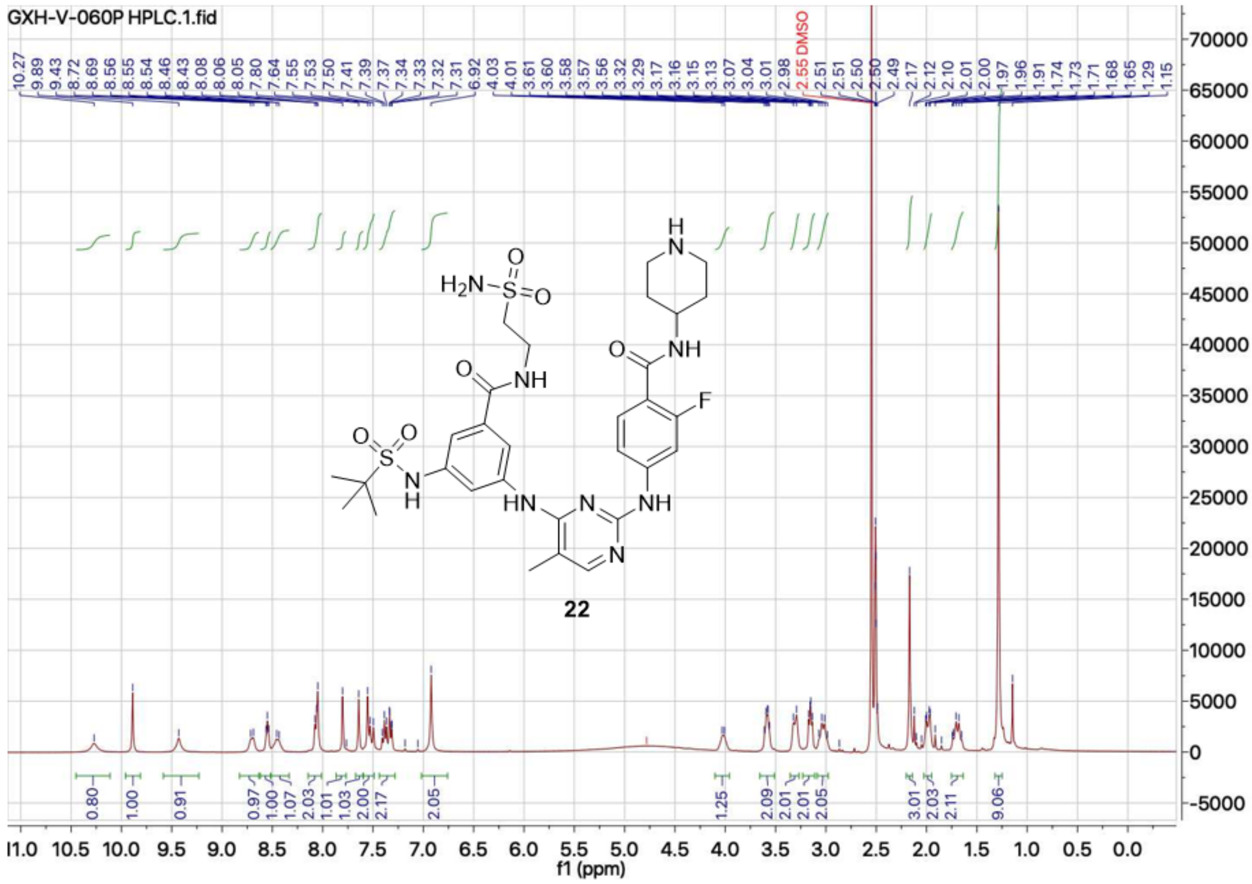
GXH-V-060P HPLC.1.fid
2.55 DMSO
22
f1 (ppm)

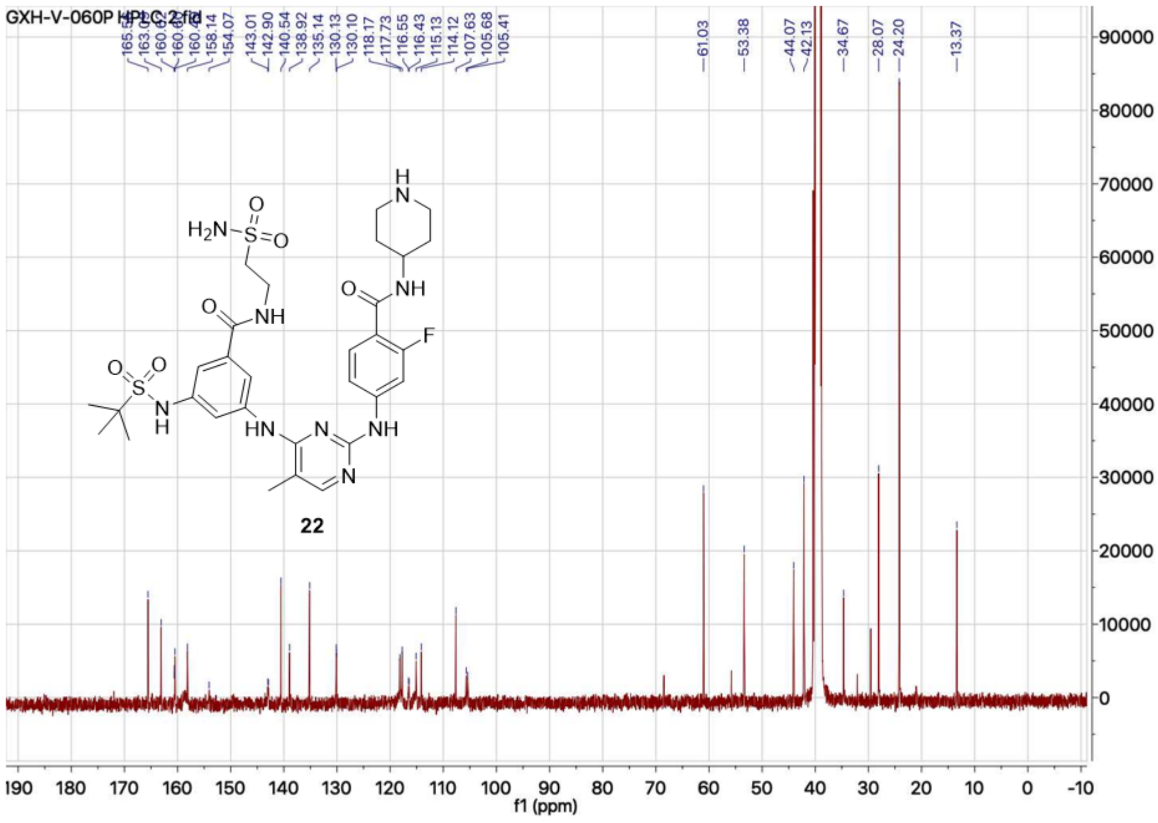
22
f1 (ppm)

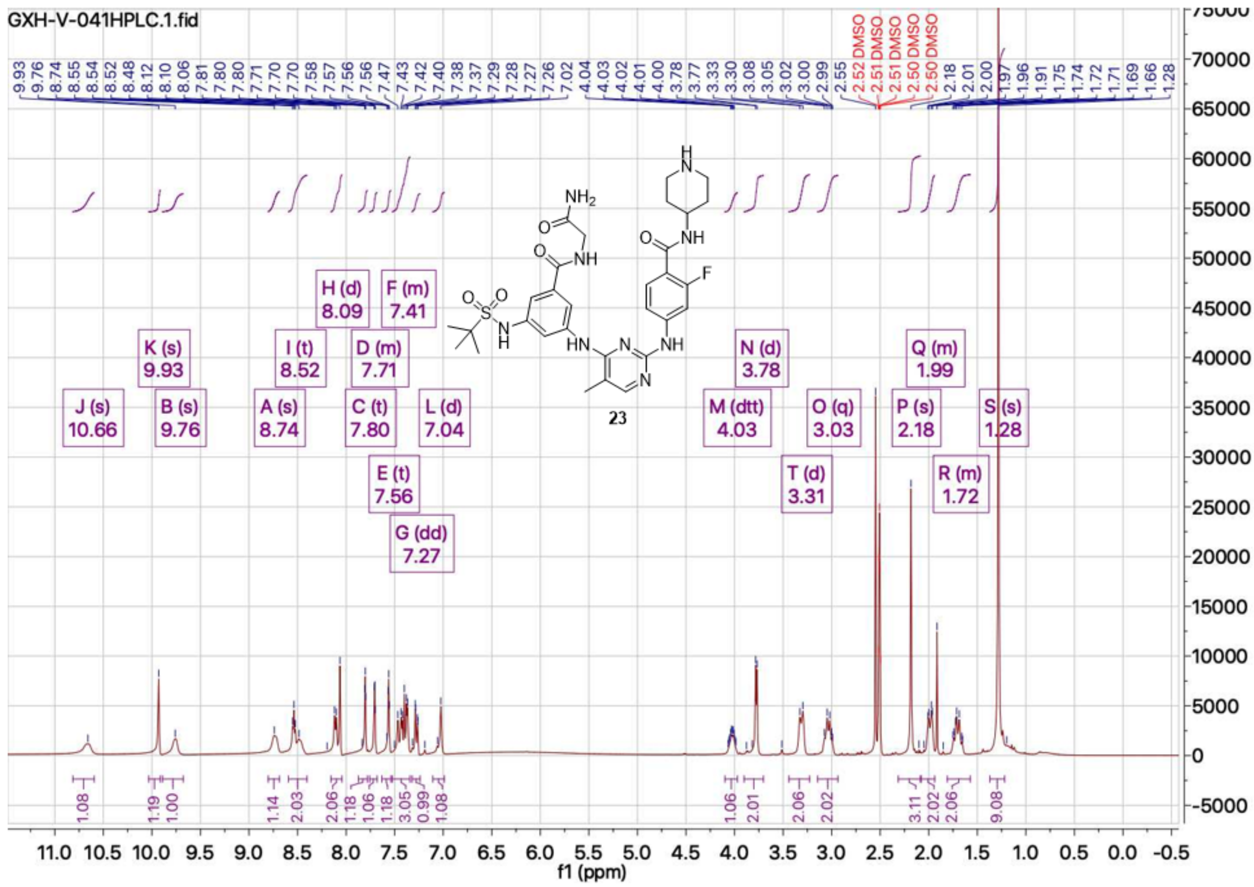
GXH-V-041HPLC.1.fid
J (s) 10.66
K (s) 9.93
B (s) 9.76
A (s) 8.74
I (t) 8.52
H (d) 8.09
C (t) 7.80
D (m) 7.71
E (t) 7.56
F (m) 7.41
G (dd) 7.27
L (d) 7.04
M (dtt) 4.03
N (d) 3.78
T (d) 3.31
O (q) 3.03
P (s) 2.18
Q (m) 1.99
R (m) 1.72
S (s) 1.28
23
f1 (ppm)

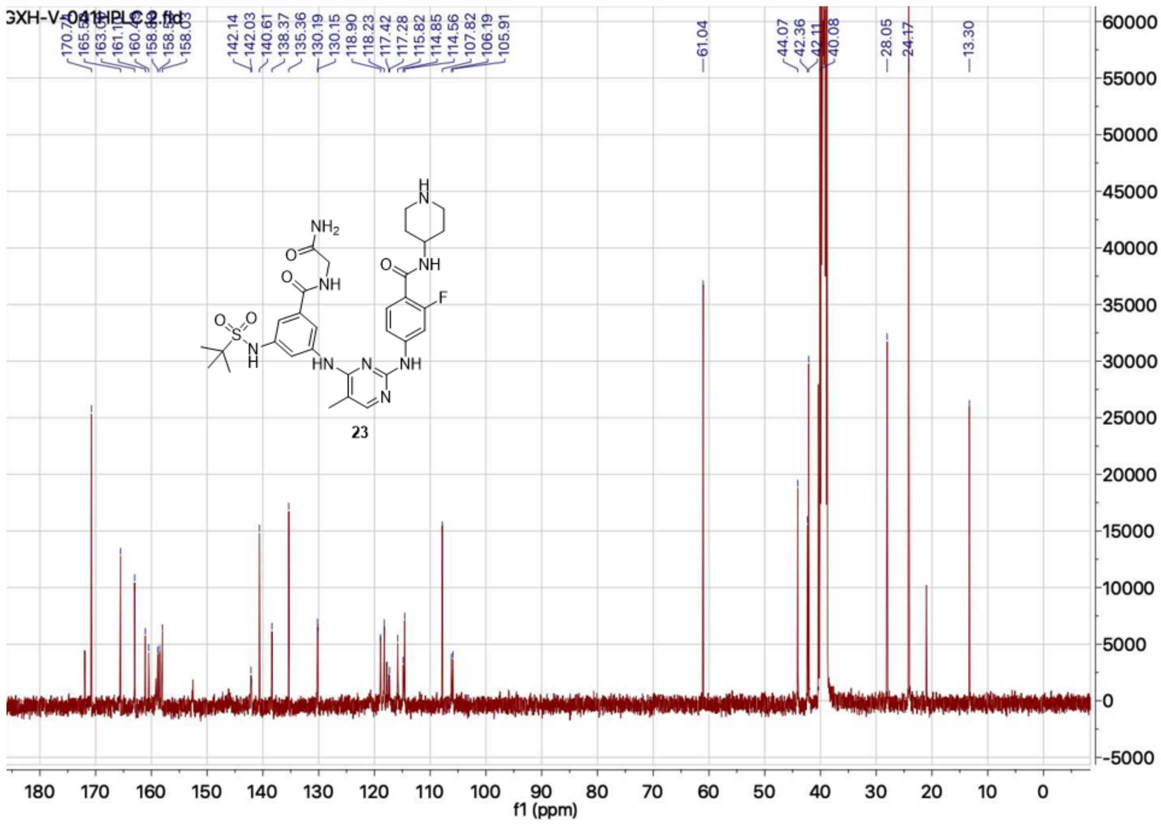
GXH-V-041HPLC.2.fid
23
f1 (ppm)

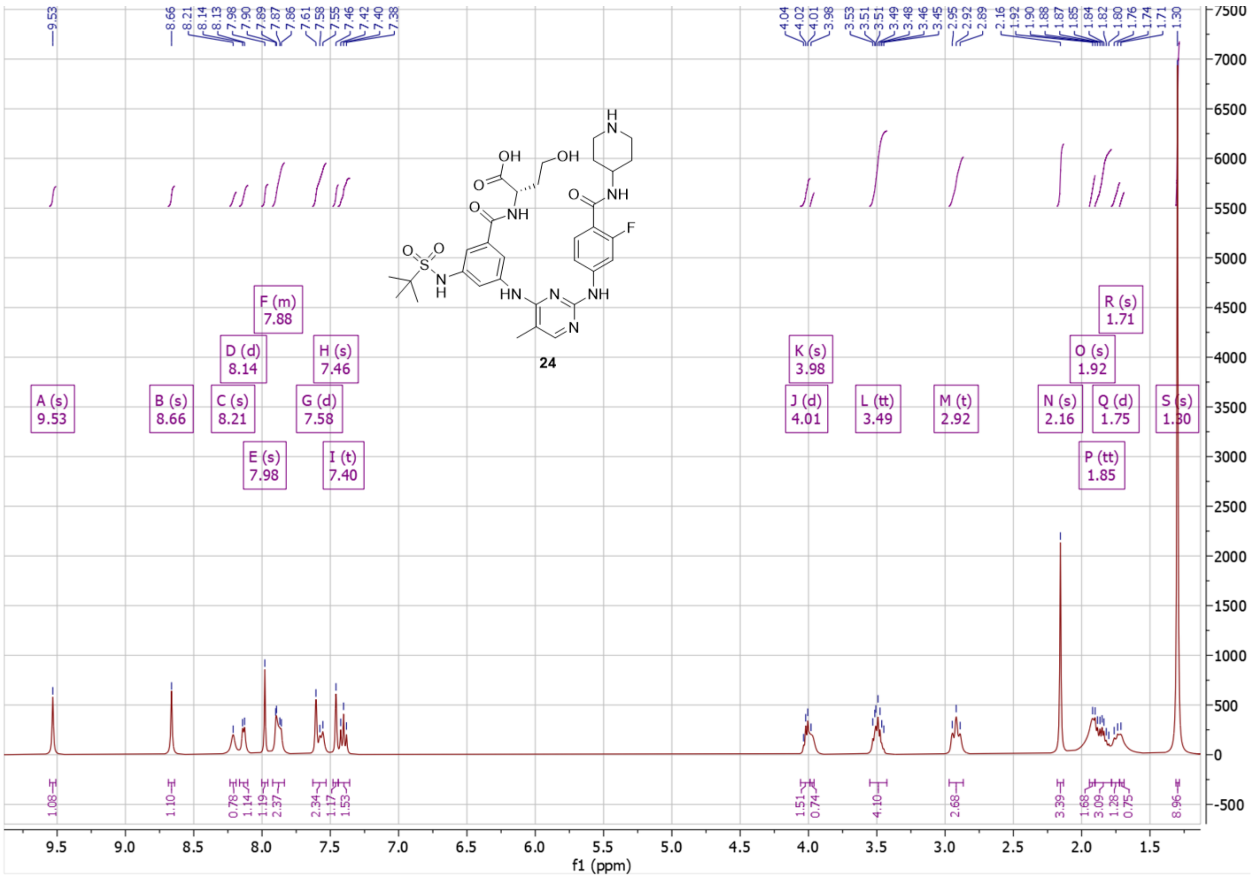

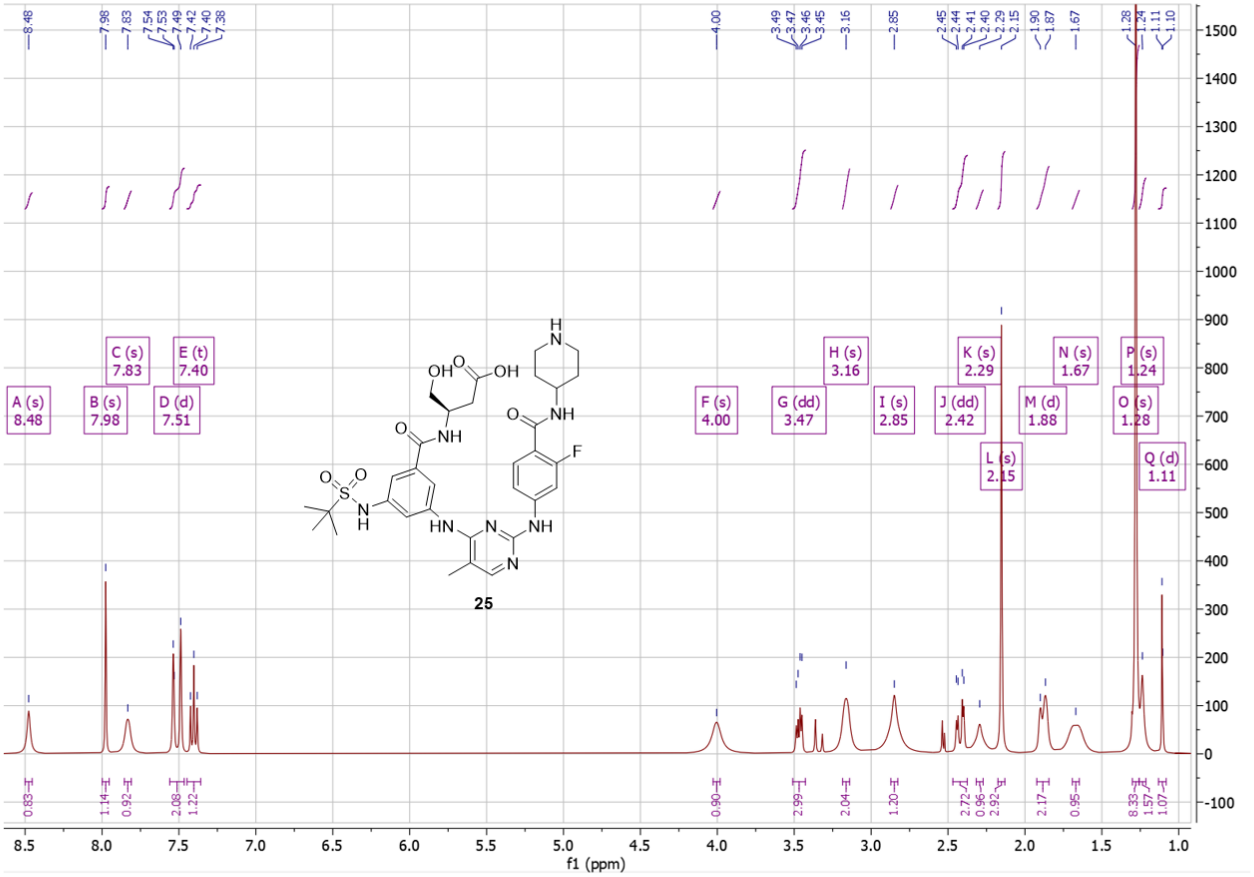
A (s) 8.48
B (s) 7.98
C (s) 7.83
D (d) 7.51
E (t) 7.40
F (s) 4.00
G (dd) 3.47
H (s) 3.16
I (s) 2.85
J (dd) 2.42
K (s) 2.29
L (s) 2.15
M (d) 1.88
N (s) 1.67
O (s) 1.28
P (s) 1.24
Q (d) 1.11
25
f1 (ppm)

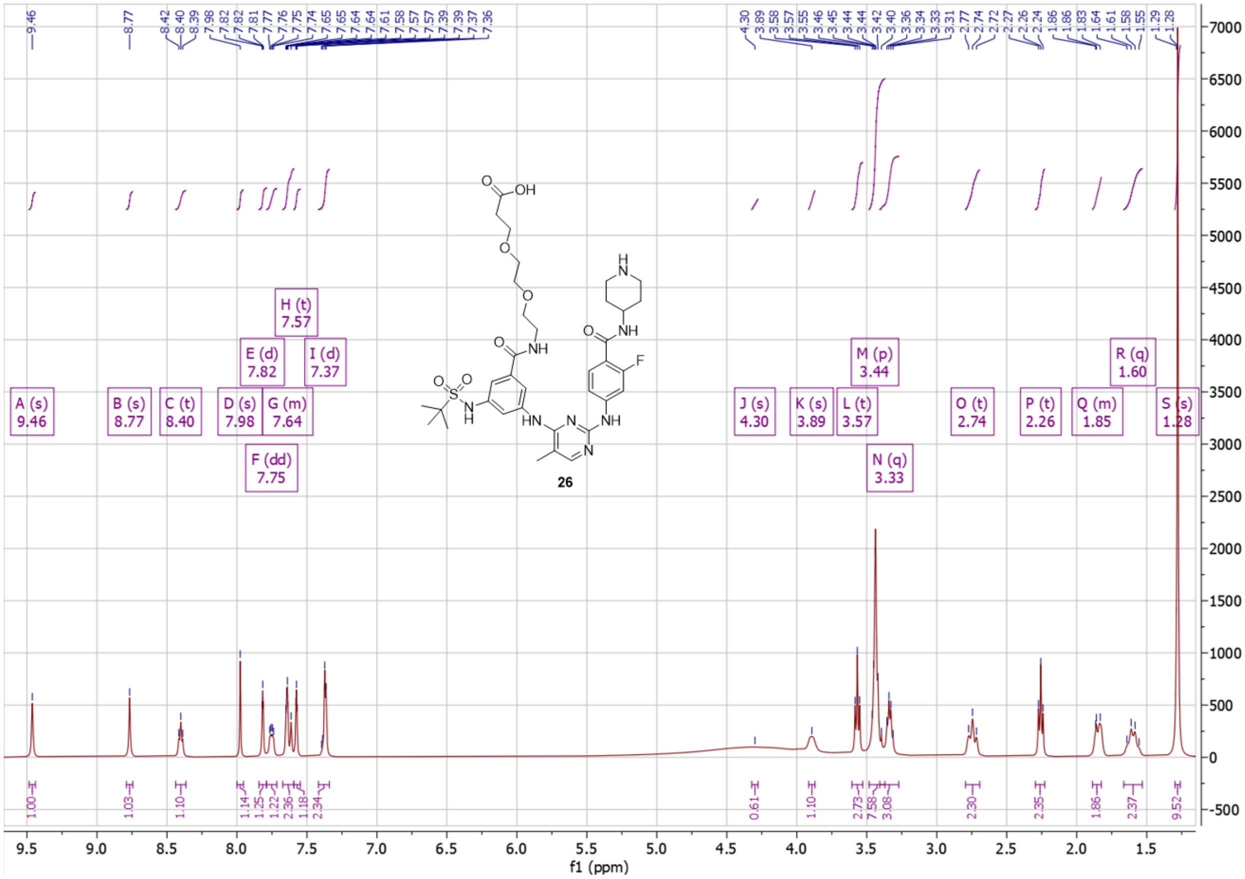
A (s) 9.46
B (s) 8.77
C (t) 8.40
D (s) 7.98
E (d) 7.82
F (dd) 7.75
G (m) 7.64
H (t) 7.57
I (d) 7.37
J (s) 4.30
K (s) 3.89
L (t) 3.57
M (p) 3.44
N (q) 3.33
O (t) 2.74
P (t) 2.26
Q (m) 1.85
R (q) 1.60
S (s) 1.28
26
f1 (ppm)

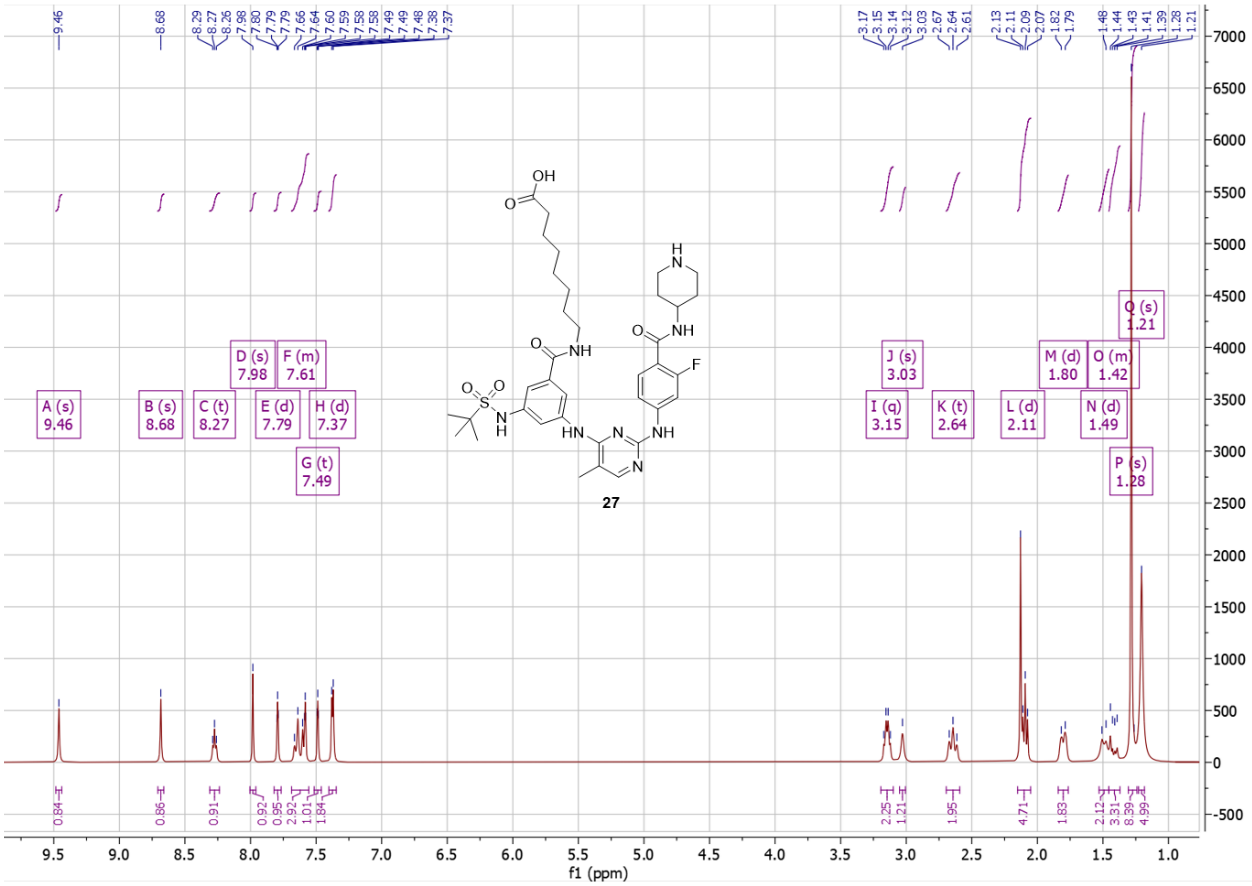
27
A (s) 9.46
B (s) 8.68
C (t) 8.27
D (s) 7.98
E (d) 7.79
F (m) 7.61
G (t) 7.49
H (d) 7.37
I (q) 3.15
J (s) 3.03
K (t) 2.64
L (d) 2.11
M (d) 1.80
N (d) 1.49
O (m) 1.42
P (s) 1.28
Q (s) 1.21
f1 (ppm)

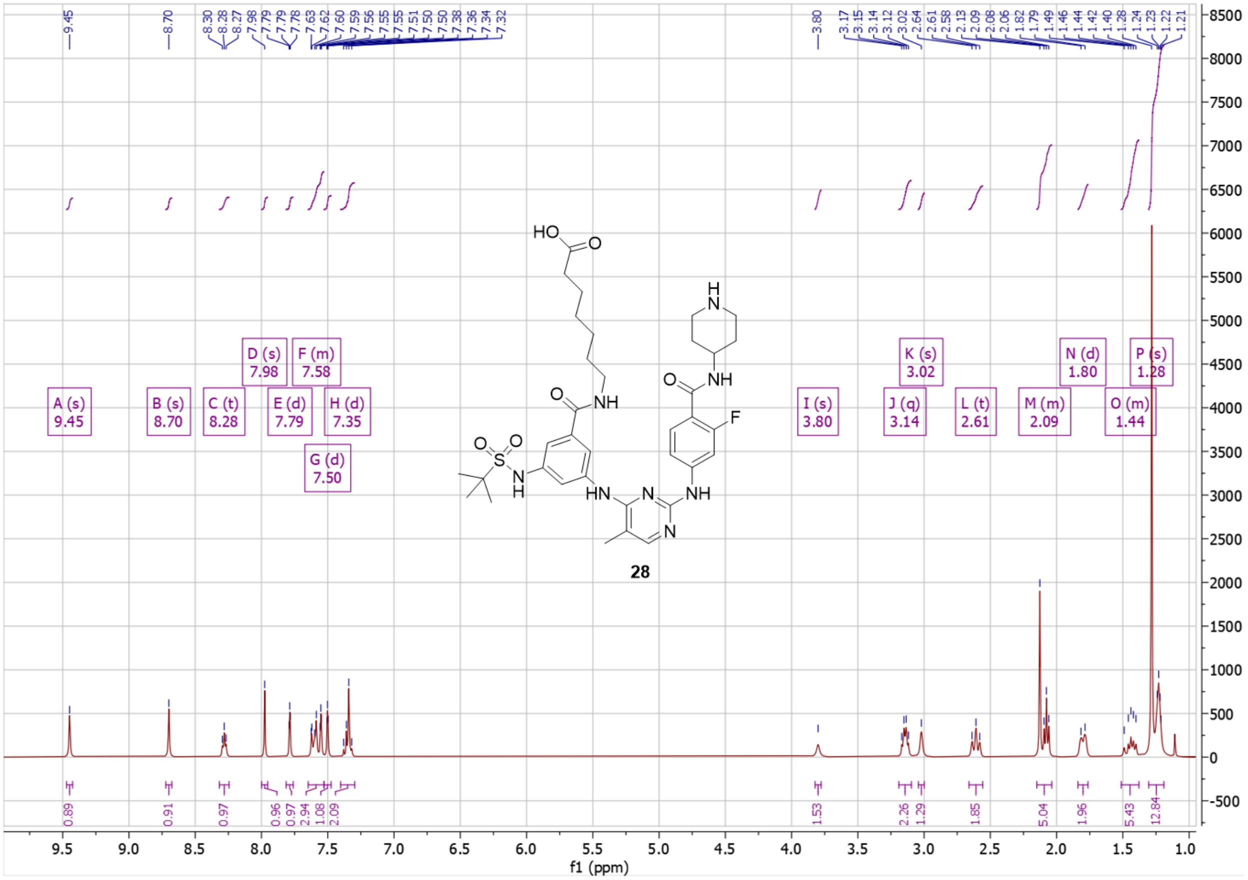

A (s) 9.45
B (s) 8.70
C (t) 8.28
D (s) 7.98
E (d) 7.79
F (m) 7.58
G (d) 7.50
H (d) 7.35
I (s) 3.80
J (q) 3.14
K (s) 3.02
L (t) 2.61
M (m) 2.09
N (d) 1.80
O (m) 1.44
P (s) 1.28
28
f1 (ppm)

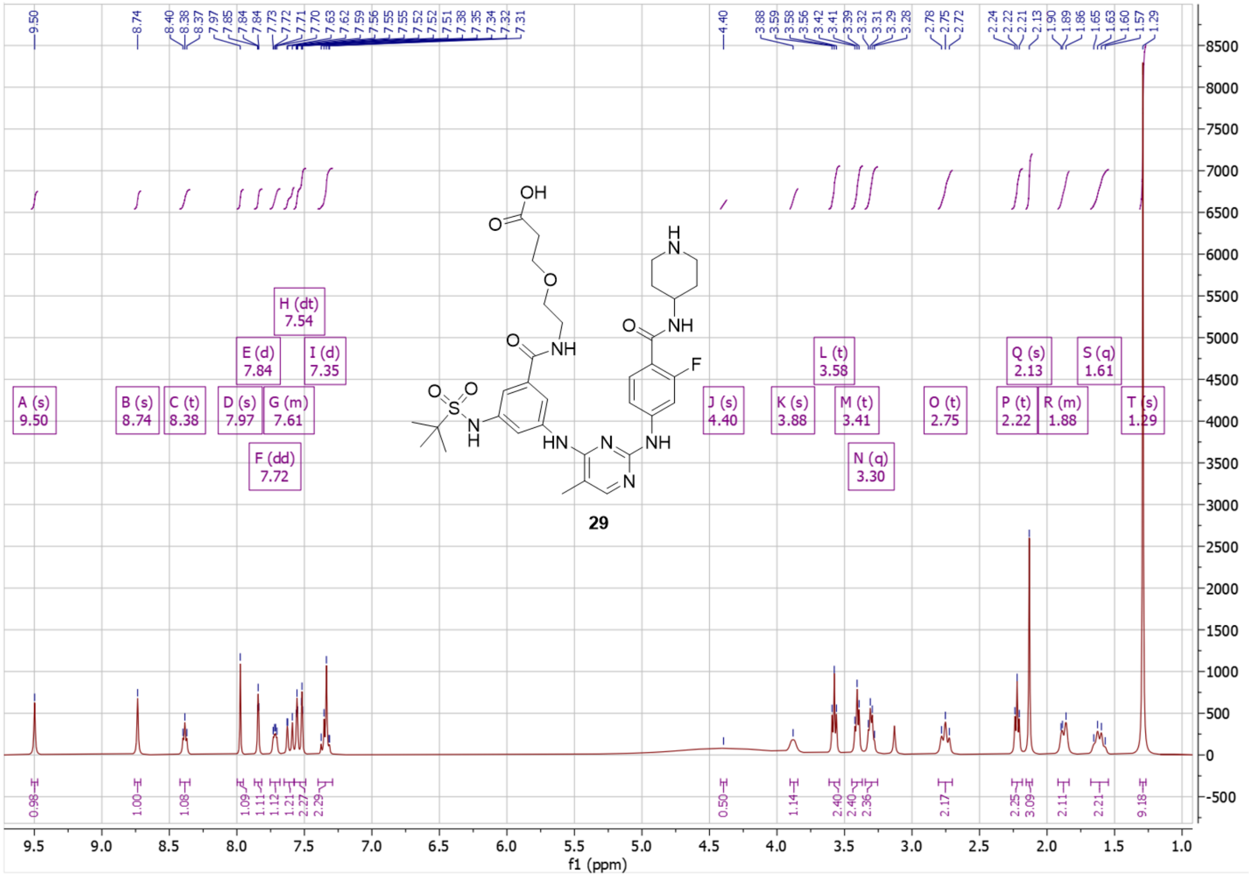

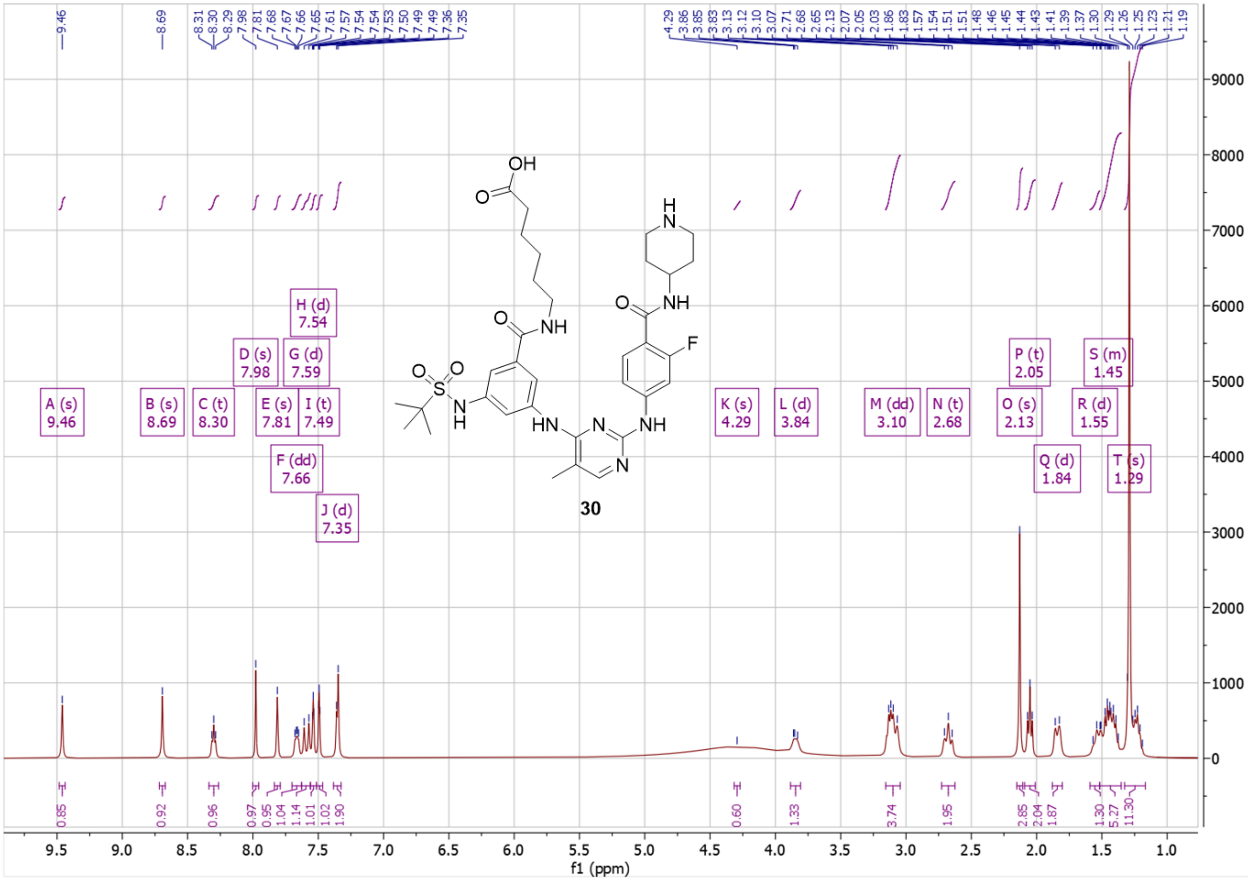
A (s) 9.46
B (s) 8.69
C (t) 8.30
D (s) 7.98
E (s) 7.81
F (dd) 7.66
G (d) 7.59
H (d) 7.54
I (t) 7.49
J (d) 7.35
K (s) 4.29
L (d) 3.84
M (dd) 3.10
N (t) 2.68
O (s) 2.13
P (t) 2.05
Q (d) 1.84
R (d) 1.55
S (m) 1.45
T (s) 1.29
30
f1 (ppm)

Single Injection Report

**Sample name:**	8		
**Description:**			
**Sample amount:**	0.000	**Sample type:**	Sample
**Instrument:**	LCMS	**Location:**	P2-F3
		**Injection:**	1 of 1
**Acq. method:**	Regular method.amx	**Injection volume:**	5.000 µL
**Analysis method:**	MS method-purity.pmx	**Acq. operator:**	SYSTEM

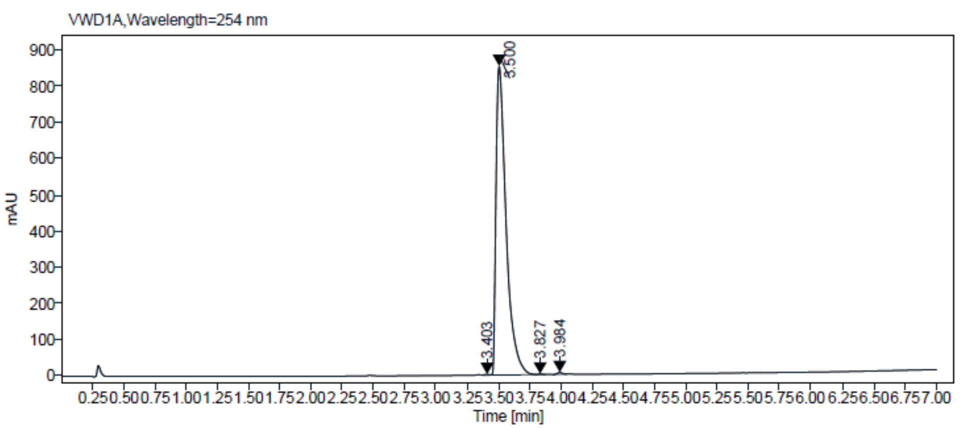

Signal: MS1 +TIC SCAN ESI Frag=110V Gain=1.0

RT [min]	Width [min]	Area	Height	Area%
3.534	0.0608	13448935.5618	9853739.2838	14.3241
3.560	0.0493	28129469.3467	10312897.2238	29.9599
3.586	0.2646	45767887.2065	9321581.7933	48.7460
4.028	0.2044	529446.2390	116930.3606	0.5639
5.261	0.7323	897428.2418	37862.4172	0.9558
6.297	0.1263	1054219.0857	256764.3985	1.1228
6.372	0.2817	2407802.5500	239331.8880	2.5645
6.675	0.3924	1655266.4014	128162.4876	1.7630
	**Sum**	**93890454.6328**		

Signal: VWD1A,Wavelength=254 nm

RT [min]	Width [min]	Area	Height	Area%
3.403	0.0785	6.8390	2.0695	0.1419
3.500	0.3544	4790.0380	853.4522	99.4018

Single Injection Report

RT [min]	Width [min]	Area	Height	Area%
3.827	0.0759	5.2666	1.7054	0.1093
3.984	0.1116	16.7219	5.4067	0.3470
	**Sum**	**4818.8656**		

Single Injection Report

**Sample name:**	9		
**Description:**			
**Sample amount:**	0.000	**Sample type:**	Sample
**Instrument:**	LCMS	**Location:**	P2-C6
		**Injection:**	1 of 1
**Acq. method:**	Regular method.amx	**Injection volume:**	5.000 µL
**Analysis method:**	MS method-purity.pmx	**Acq. operator:**	SYSTEM

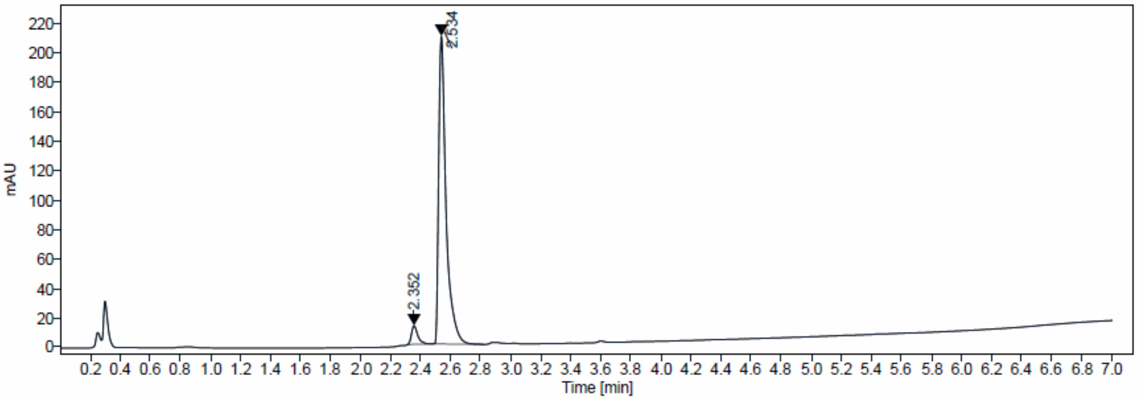

Signal: MS1 +TIC SCAN ESI Frag=110V Gain=1.0

RT [min]	Width [min]	Area	Height	Area%
0.310	0.5775	886981.4636	121470.6997	9.9858
2.405	0.3066	751471.1907	95725.5542	8.4602
2.593	0.3858	5697086.5763	980297.8299	64.1387
6.291	0.1190	552236.6902	155205.4154	6.2172
6.352	0.2680	994677.2786	145533.8670	11.1982
	**Sum**	**8882453.1996**		

Signal: VWD1A,Wavelength=254 nm

RT [min]	Width [min]	Area	Height	Area%
2.352	0.1656	36.6167	12.5501	4.8848
2.534	0.3450	712.9895	208.6395	95.1152
	**Sum**	**749.6062**		

Single Injection Report

**Sample name:**	10		
**Description:**			
**Sample amount:**	0.000	**Sample type:**	Sample
**Instrument:**	LCMS	**Location:**	P2-F4
		**Injection:**	1 of 1
**Acq. method:**	Regular method.amx	**Injection volume:**	5.000 µL
**Analysis method:**	MS method-purity.pmx	**Acq. operator:**	SYSTEM

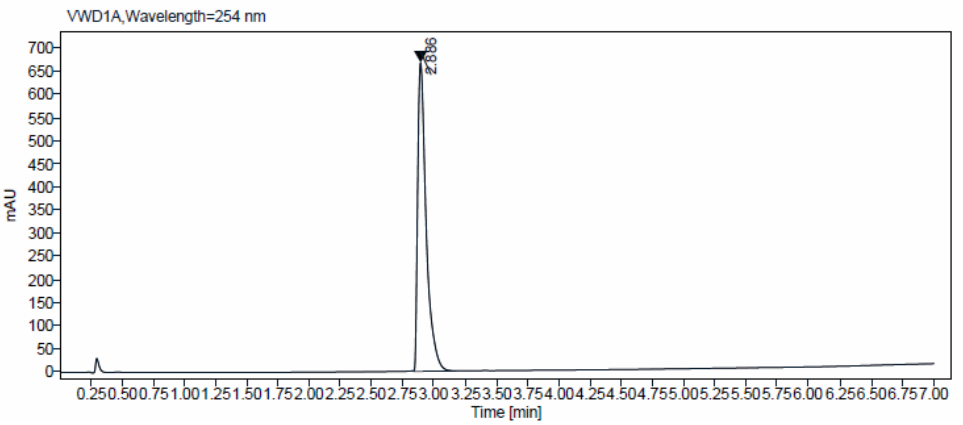

Signal: MS1 +TIC SCAN ESI Frag=110V Gain=1.0

RT [min]	Width [min]	Area	Height	Area%
2.947	0.4132	47349335.0296	7026374.1526	89.2743
6.293	0.1038	1045603.9927	282249.5281	1.9714
6.360	0.2990	3004512.3762	266745.0492	5.6648
6.756	0.3408	1638582.3918	143130.9732	3.0894
	**Sum**	**53038033.7903**		

Signal: VWD1A,Wavelength=254 nm

RT [min]	Width [min]	Area	Height	Area%
2.886	0.3404	3167.4142	668.5170	100.0000
	**Sum**	**3167.4142**		

Single Injection Report

**Sample name:**	11		
**Description:**			
**Sample amount:**	0.000	**Sample type:**	Sample
**Instrument:**	LCMS	**Location:**	P2-F5
		**Injection:**	1 of 1
**Acq. method:**	Regular method.amx	**Injection volume:**	5.000 µL
**Analysis method:**	MS method-purity.pmx	**Acq. operator:**	SYSTEM

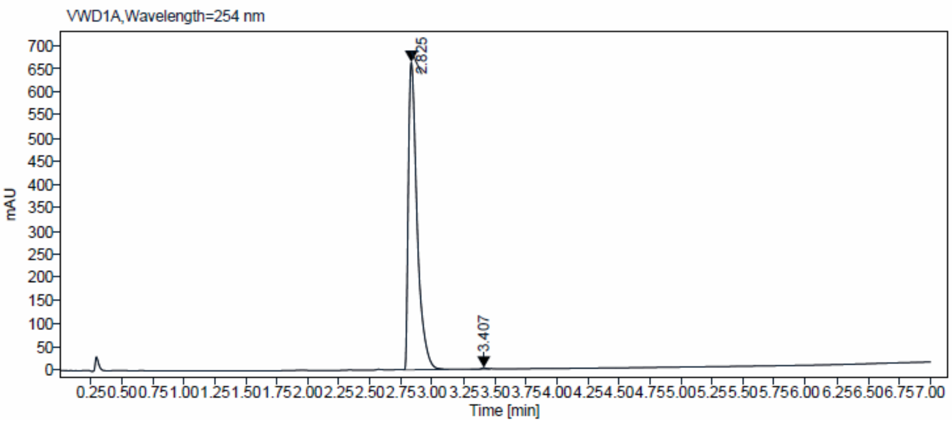

Signal: MS1 +TIC SCAN ESI Frag=110V Gain=1.0

RT [min]	Width [min]	Area	Height	Area%
2.869	0.1816	12565414.1291	7100009.9765	22.4278
2.890	0.3814	38447824.8069	7359340.0782	68.6249
6.301	0.1199	1011014.1835	300442.5787	1.8045
6.370	0.2832	2456190.7455	251696.1026	4.3840
6.659	0.3790	1545587.5341	126347.7576	2.7587
	**Sum**	**56026031.3990**		

Signal: VWD1A,Wavelength=254 nm

RT [min]	Width [min]	Area	Height	Area%
2.825	0.3640	3257.9498	663.3281	99.7561
3.407	0.1525	7.9653	2.6826	0.2439
	**Sum**	**3265.9151**		

Single Injection Report

**Sample name:**	12		
**Description:**			
**Sample amount:**	0.000	**Sample type:**	Sample
**Instrument:**	LCMS	**Location:**	P2-C11
		**Injection:**	1 of 1
**Acq. method:**	Regular method.amx	**Injection volume:**	5.000 µL
**Analysis method:**	MS method-purity.pmx	**Acq. operator:**	SYSTEM

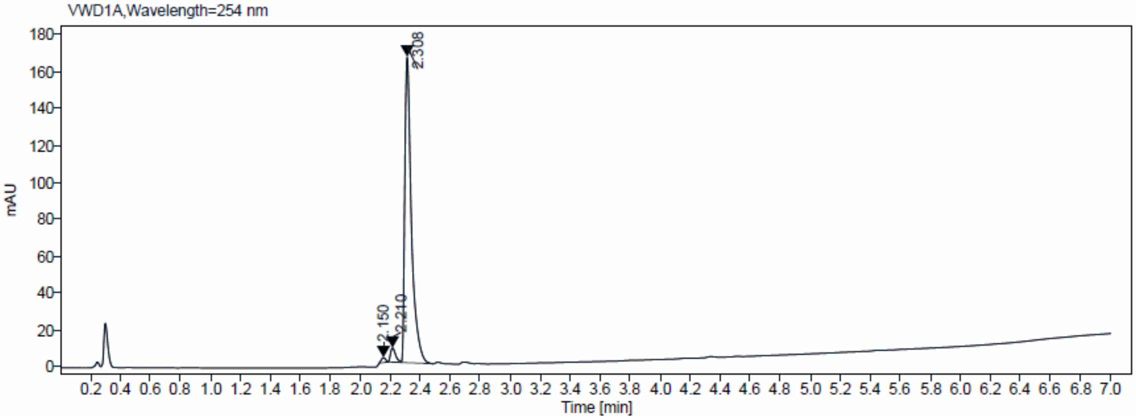

Signal: MS1 +TIC SCAN ESI Frag=110V Gain=1.0

RT [min]	Width [min]	Area	Height	Area%
0.309	0.4204	543341.7230	73915.0082	4.7489
2.377	0.3553	4465517.6382	871769.4540	39.0295
6.166	0.3880	904022.7323	116896.5626	7.9013
6.290	0.1156	1149820.0987	269794.8370	10.0497
6.366	0.6792	4378680.1794	249524.0275	38.2706
	**Sum**	**11441382.3715**		

Signal: VWD1A,Wavelength=254 nm

RT [min]	Width [min]	Area	Height	Area%
2.150	0.0589	5.2066	2.6249	0.9752
2.210	0.0877	17.9611	7.5888	3.3642
2.308	0.2141	510.7209	165.5658	95.6606
	**Sum**	**533.8887**		

Single Injection Report

**Sample name:**	13		
**Description:**			
**Sample amount:**	0.000	**Sample type:**	Sample
**Instrument:**	LCMS	**Location:**	P2-D6
		**Injection:**	1 of 1
**Acq. method:**	Regular method.amx	**Injection volume:**	5.000 µL
**Analysis method:**	MS method-purity.pmx	**Acq. operator:**	SYSTEM

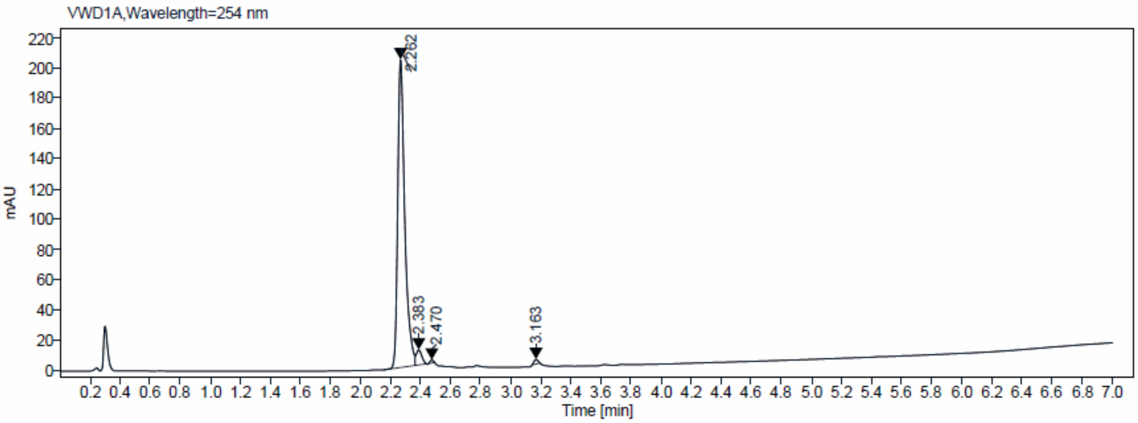

Signal: MS1 +TIC SCAN ESI Frag=110V Gain=1.0

RT [min]	Width [min]	Area	Height	Area%
2.320	0.3136	5257845.4762	1098174.3641	46.6568
2.523	0.2331	553335.7585	71139.9622	4.9102
6.291	0.4137	3894056.3553	242114.1572	34.5549
6.645	0.3970	1563965.6614	131363.6099	13.8782
	**Sum**	**11269203.2515**		

Signal: VWD1A,Wavelength=254 nm

RT [min]	Width [min]	Area	Height	Area%
2.262	0.2109	662.6369	203.5833	95.1670
2.383	0.0745	25.8174	9.9108	3.7079
2.470	0.0552	2.4429	1.9050	0.3508
3.163	0.0517	5.3914	2.9433	0.7743
	**Sum**	**696.2886**		

Single Injection Report

**Sample name:**	14		
**Description:**			
**Sample amount:**	0.000	**Sample type:**	Sample
**Instrument:**	LCMS	**Location:**	P2-F6
		**Injection:**	1 of 1
**Acq. method:**	Regular method.amx	**Injection volume:**	5.000 µL
**Analysis method:**	MS method-purity.pmx	**Acq. operator:**	SYSTEM

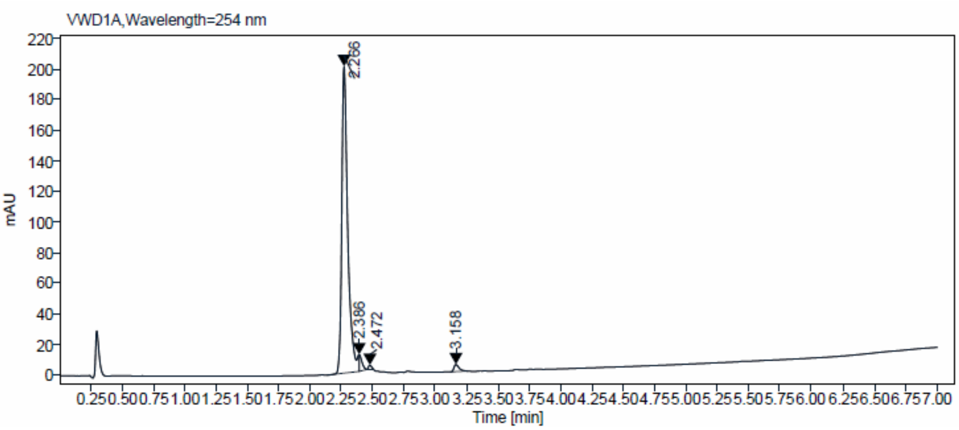

Signal: MS1 +TIC SCAN ESI Frag=110V Gain=1.0

RT [min]	Width [min]	Area	Height	Area%
2.328	0.3058	7056439.0332	1373037.2433	50.5485
2.524	0.1875	545102.2735	92486.6880	3.9048
3.216	0.5109	667160.1279	88273.2040	4.7792
6.299	0.0899	816867.0617	273856.0366	5.8516
6.341	0.2969	3054102.4057	233796.3984	21.8779
6.644	0.1140	842062.8861	131358.6075	6.0321
6.756	0.2485	978007.6199	134713.5751	7.0059
	**Sum**	**13959741.4081**		

Signal: VWD1A,Wavelength=254 nm

RT [min]	Width [min]	Area	Height	Area%
2.266	0.2557	665.0011	200.3578	95.0765
2.386	0.0592	16.4253	10.9187	2.3484
2.472	0.0552	4.8598	2.6482	0.6948
3.158	0.1334	13.1514	4.5825	1.8803
	**Sum**	**699.4376**		

Single Injection Report

**Sample name:**	15		
**Description:**			
**Sample amount:**	0.000	**Sample type:**	Sample
**Instrument:**	LCMS	**Location:**	P2-A7
		**Injection:**	1 of 1
**Acq. method:**	Regular method.amx	**Injection volume:**	5.000 µL
**Analysis method:**	MS method-purity.pmx	**Acq. operator:**	SYSTEM

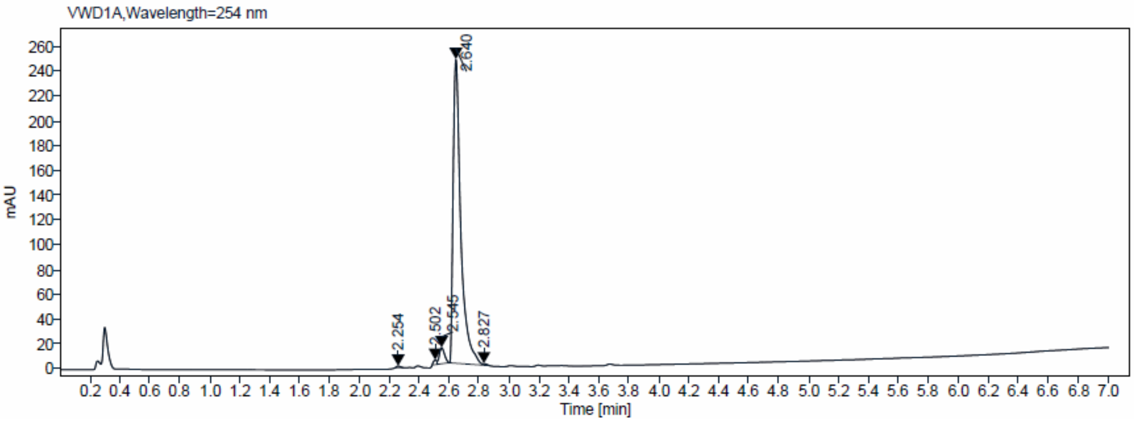

Signal: MS1 +TIC SCAN ESI Frag=110V Gain=1.0

RT [min]	Width [min]	Area	Height	Area%
0.316	0.9538	958655.2407	95083.3176	5.5689
2.596	0.1493	704536.1776	158417.7882	4.0927
2.700	0.3828	8711003.6579	1523407.0020	50.6028
6.168	0.5992	1345610.5098	108921.1272	7.8167
6.298	0.1123	1095081.3682	234417.2419	6.3614
6.372	0.2753	2497208.0226	230300.3069	14.5065
6.646	0.1079	838183.2303	137394.7575	4.8691
6.742	0.2848	1064177.3445	137580.6520	6.1819
	**Sum**	**17214455.5516**		

Signal: VWD1A,Wavelength=254 nm

RT [min]	Width [min]	Area	Height	Area%
2.254	0.0577	2.5836	1.3686	0.2823
2.502	0.0337	5.3778	3.5059	0.5876
2.545	0.0780	31.2068	12.9278	3.4096

Single Injection Report

RT [min]	Width [min]	Area	Height	Area%
2.640	0.2237	873.5563	245.2020	95.4422
2.827	0.0501	2.5478	1.2911	0.2784
	Sum	915.2723		

Single Injection Report

**Sample name:**	16		
**Description:**			
**Sample amount:**	0.000	**Sample type:**	Sample
**Instrument:**	LCMS	**Location:**	P2-C8
		**Injection:**	1 of 1
**Acq. method:**	Regular method.amx	**Injection volume:**	5.000 µL
**Analysis method:**	MS method-purity.pmx	**Acq. operator:**	SYSTEM

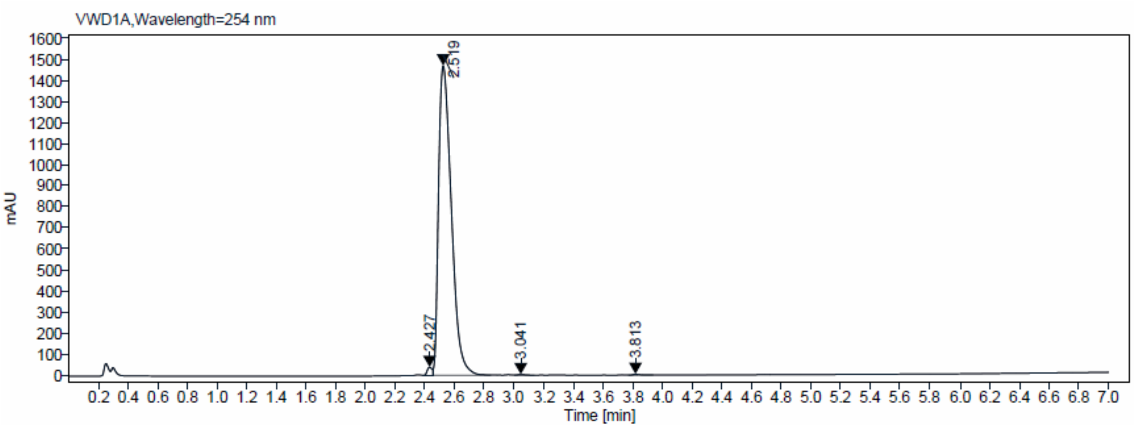

Signal: MS1 +TIC SCAN ESI Frag=110V Gain=1.0

RT [min]	Width [min]	Area	Height	Area%
0.307	0.2967	745972.6002	185041.9669	1.3403
2.560	0.3545	15019857.8096	5381850.0833	26.9862
2.586	0.3948	35489481.7467	5622713.1252	63.7641
3.873	0.4757	903314.1233	161639.6236	1.6230
4.718	0.3392	830528.4197	113551.7822	1.4922
5.280	0.5102	841033.7020	76862.2018	1.5111
6.292	0.1179	752775.8743	195904.0489	1.3525
6.362	0.2738	1074492.0348	155013.5046	1.9305
	**Sum**	**55657456.3108**		

Signal: VWD1A,Wavelength=254 nm

RT [min]	Width [min]	Area	Height	Area%
2.427	0.0644	80.7692	37.0667	0.9101
2.519	0.3900	8770.4168	1467.2719	98.8242
3.041	0.1334	10.6362	3.0192	0.1198

Single Injection Report

RT [min]	Width [min]	Area	Height	Area%
3.813	0.1667	12.9435	4.0890	0.1458
	**Sum**	**8874.7657**		

Single Injection Report

**Sample name:**	17		
**Description:**			
**Sample amount:**	0.000	**Sample type:**	Sample
**Instrument:**	LCMS	**Location:**	P2-F7
		**Injection:**	1 of 1
**Acq. method:**	Regular method.amx	**Injection volume:**	5.000 µL
**Analysis method:**	MS method-purity.pmx	**Acq. operator:**	SYSTEM

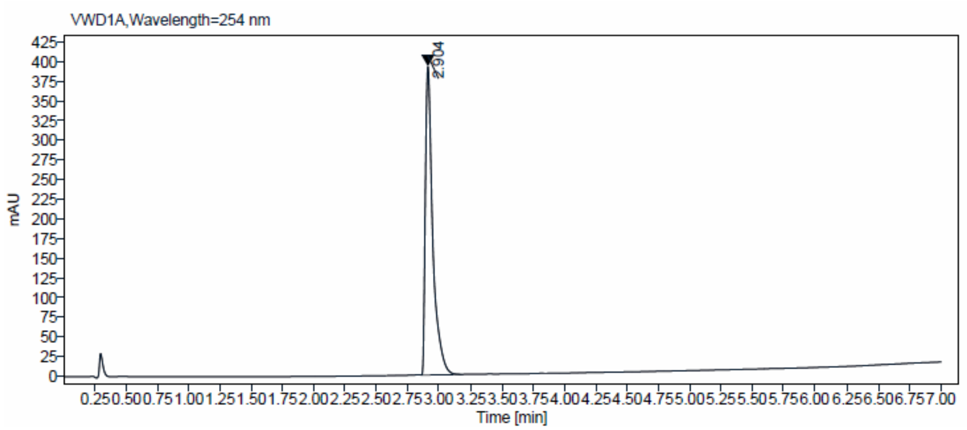

Signal: MS1 +TIC SCAN ESI Frag=110V Gain=1.0

RT [min]	Width [min]	Area	Height	Area%
2.954	0.3781	17631806.5356	5644277.9905	43.0357
2.993	0.4046	17137525.5401	4825051.3695	41.8292
5.243	0.6634	758742.3496	43621.3032	1.8519
6.300	0.1015	852688.4666	273313.8242	2.0812
6.370	0.2686	2548408.5448	245857.9748	6.2202
6.695	0.3369	2041034.2236	168597.0615	4.9818
	**Sum**	**40970205.6603**		

Signal: VWD1A,Wavelength=254 nm

RT [min]	Width [min]	Area	Height	Area%
2.904	0.3227	1625.4092	393.1853	100.0000
	**Sum**	**1625.4092**		

Single Injection Report

**Sample name:**	18		
**Description:**			
**Sample amount:**	0.000	**Sample type:**	Sample
**Instrument:**	LCMS	**Location:**	P2-F8
		**Injection:**	1 of 1
**Acq. method:**	Regular method.amx	**Injection volume:**	5.000 µL
**Analysis method:**	MS method-purity.pmx	**Acq. operator:**	SYSTEM

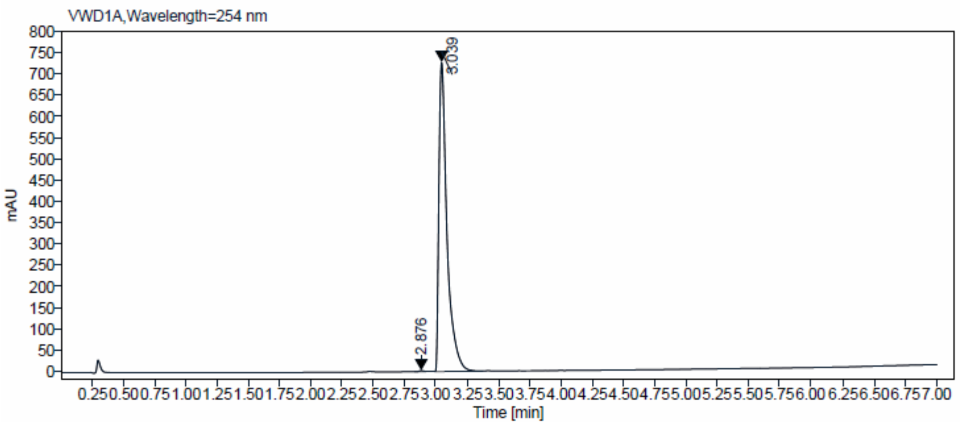

Signal: MS1 +TIC SCAN ESI Frag=110V Gain=1.0

RT [min]	Width [min]	Area	Height	Area%
3.100	0.4055	52034597.4773	7323809.7410	90.8502
6.303	0.1152	1008592.2342	287520.2886	1.7610
6.373	0.2510	2310193.9161	235286.3889	4.0335
6.700	0.1367	919155.8299	132066.4925	1.6048
6.747	0.2909	1002628.4588	145306.9972	1.7505
	**Sum**	**57275167.9163**		

Signal: VWD1A,Wavelength=254 nm

RT [min]	Width [min]	Area	Height	Area%
2.876	0.0932	5.6227	2.1807	0.1715
3.039	0.3871	3273.4377	727.8808	99.8285
	**Sum**	**3279.0604**		

Single Injection Report

**Sample name:**	19		
**Description:**			
**Sample amount:**	0.000	**Sample type:**	Sample
**Instrument:**	LCMS	**Location:**	P2-A10
		**Injection:**	1 of 1
**Acq. method:**	Regular method.amx	**Injection volume:**	5.000 µL
**Analysis method:**	MS method-purity.pmx	**Acq. operator:**	SYSTEM

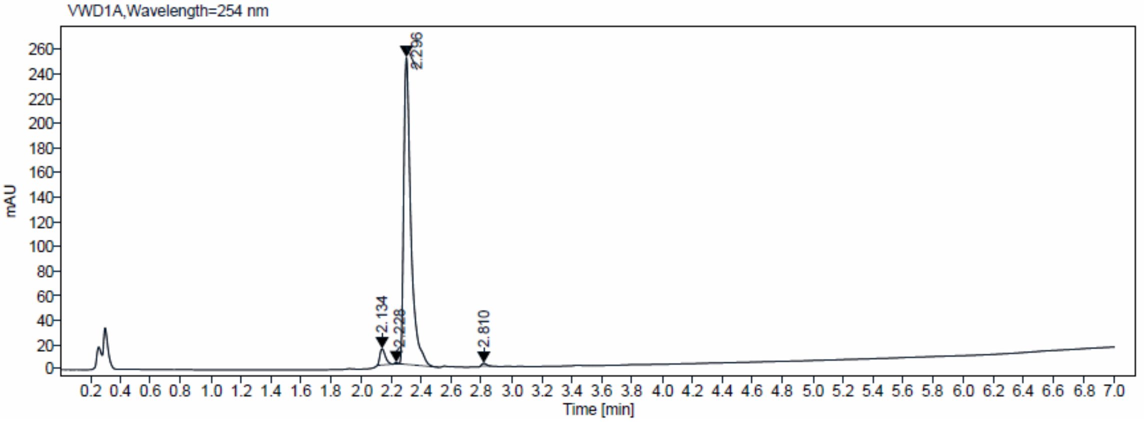

Signal: MS1 +TIC SCAN ESI Frag=110V Gain=1.0

RT [min]	Width [min]	Area	Height	Area%
0.319	0.3215	698495.9662	106329.1373	7.1209
2.189	0.2265	511233.3023	91078.2415	5.2118
2.353	0.4771	5894510.5316	1163958.8831	60.0923
6.290	0.1023	800062.7176	208152.4572	8.1563
6.357	0.2622	1285474.2115	175431.3681	13.1049
6.706	0.2519	619325.3055	70636.9822	6.3138
	**Sum**	**9809102.0347**		

Signal: VWD1A,Wavelength=254 nm

RT [min]	Width [min]	Area	Height	Area%
2.134	0.1023	33.3269	13.0948	3.8480
2.228	0.0433	1.5031	1.0266	0.1735
2.296	0.2639	826.7320	249.6500	95.4575
2.810	0.0698	4.5112	2.1671	0.5209
	**Sum**	**866.0732**		

Single Injection Report

**Sample name:**	20		
**Description:**			
**Sample amount:**	0.000	**Sample type:**	Sample
**Instrument:**	LCMS	**Location:**	P2-F9
		**Injection:**	1 of 1
**Acq. method:**	Regular method.amx	**Injection volume:**	5.000 µL
**Analysis method:**	MS method-purity.pmx	**Acq. operator:**	SYSTEM

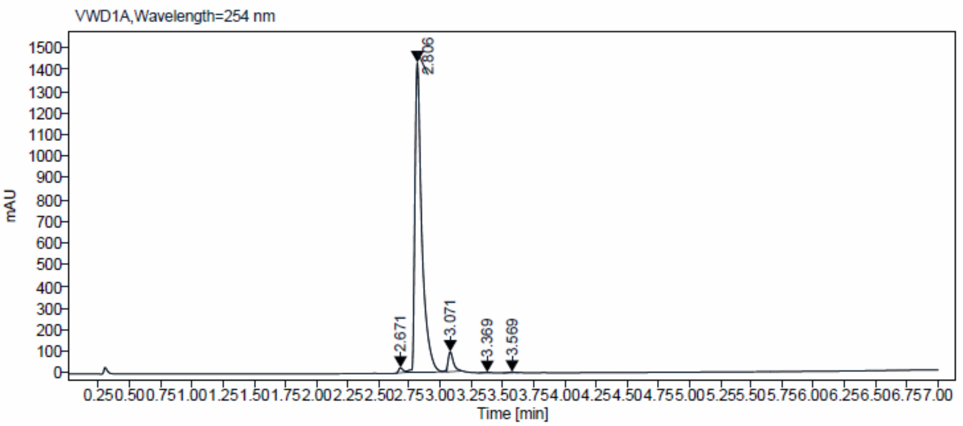

Signal: MS1 +TIC SCAN ESI Frag=110V Gain=1.0

RT [min]	Width [min]	Area	Height	Area%
2.728	0.1414	766317.6079	229826.7954	1.0708
2.865	0.3005	56283283.6292	7982564.7805	78.6469
3.130	0.3075	9682400.4614	1821339.9436	13.5296
3.626	0.2653	784686.8036	155845.9670	1.0965
6.299	0.1043	675739.9938	205947.0776	0.9442
6.353	0.2840	1990516.0021	244425.2778	2.7814
6.652	0.1210	681033.6797	109205.8178	0.9516
6.748	0.2723	700515.7553	103730.2900	0.9789
	**Sum**	**71564493.9329**		

Signal: VWD1A,Wavelength=254 nm

RT [min]	Width [min]	Area	Height	Area%
2.671	0.0810	56.4694	22.7969	0.9578
2.806	0.3287	5559.2304	1428.5543	94.2940
3.071	0.1233	264.1120	91.6498	4.4798

Single Injection Report

RT [min]	Width [min]	Area	Height	Area%
3.369	0.1034	3.8104	1.4277	0.0646
3.569	0.1311	12.0107	3.5542	0.2037
	**Sum**	**5895.6330**		

Single Injection Report

**Sample name:**	21		
**Description:**			
**Sample amount:**	0.000	**Sample type:**	Sample
**Instrument:**	LCMS	**Location:**	P2-F10
		**Injection:**	1 of 1
**Acq. method:**	Regular method.amx	**Injection volume:**	5.000 µL
**Analysis method:**	MS method-purity.pmx	**Acq. operator:**	SYSTEM

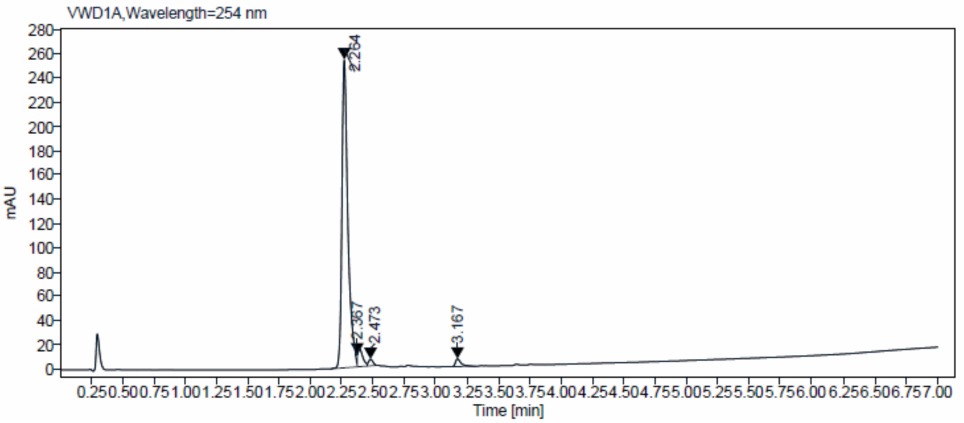

Signal: MS1 +TIC SCAN ESI Frag=110V Gain=1.0

RT [min]	Width [min]	Area	Height	Area%
2.327	0.5495	9998941.2795	1916237.2173	72.6561
3.210	0.2723	518474.3006	101989.5039	3.7674
6.289	0.0849	780123.1249	260317.1772	5.6687
6.339	0.1172	1041314.4159	211309.2688	7.5666
6.687	0.1623	641785.4694	124029.5911	4.6635
6.743	0.2407	781367.1141	152323.3354	5.6777
	**Sum**	**13762005.7044**		

Signal: VWD1A,Wavelength=254 nm

RT [min]	Width [min]	Area	Height	Area%
2.264	0.2935	879.9806	254.1468	95.8695
2.367	0.0146	4.7574	9.1975	0.5183
2.473	0.0773	13.2053	5.2947	1.4387
3.167	0.1945	19.9509	6.2212	2.1736
	**Sum**	**917.8942**		

Single Injection Report

**Sample name:**	22		
**Description:**			
**Sample amount:**	0.000	**Sample type:**	Sample
**Instrument:**	LCMS	**Location:**	P2-C9
		**Injection:**	1 of 1
**Acq. method:**	Regular method.amx	**Injection volume:**	5.000 µL
**Analysis method:**	MS method-purity.pmx	**Acq. operator:**	SYSTEM

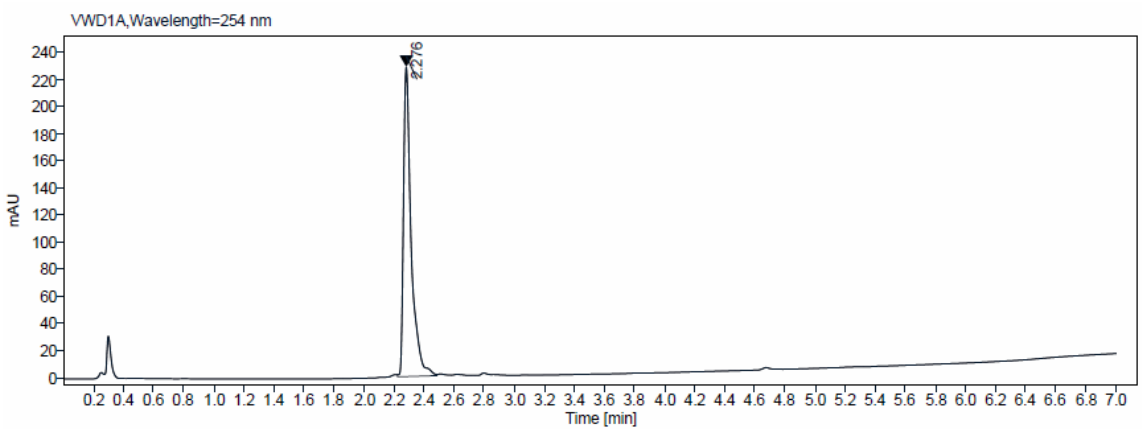

Signal: MS1 +TIC SCAN ESI Frag=110V Gain=1.0

RT [min]	Width [min]	Area	Height	Area%
0.314	0.5620	1098800.36 90	154199.0567	7.0037
2.333	0.5116	7209855.11 21	1330463.60 53	45.9554
2.687	0.5422	775691.2858	44844.4152	4.9442
4.722	0.3390	1340407.07 26	209635.6062	8.5437
5.310	0.4571	980281.8766	116403.1319	6.2483
6.286	0.1030	665720.1926	256697.3068	4.2433
6.366	0.3293	2326704.28 10	192105.3238	14.8303
6.668	0.3611	1291354.33 31	108435.0744	8.2311
	**Sum**	**15688814.52 28**		

Signal: VWD1A,Wavelength=254 nm

RT [min]	Width [min]	Area	Height	Area%
2.276	0.2626	812.5129	228.3777	100.0000
	**Sum**	**812.5129**		

Single Injection Report

**Sample name:**	23		
**Description:**			
**Sample amount:**	0.000	**Sample type:**	Sample
**Instrument:**	LCMS	**Location:**	P2-F11
		**Injection:**	1 of 1
**Acq. method:**	Regular method.amx	**Injection volume:**	5.000 µL
**Analysis method:**	MS method-purity.pmx	**Acq. operator:**	SYSTEM

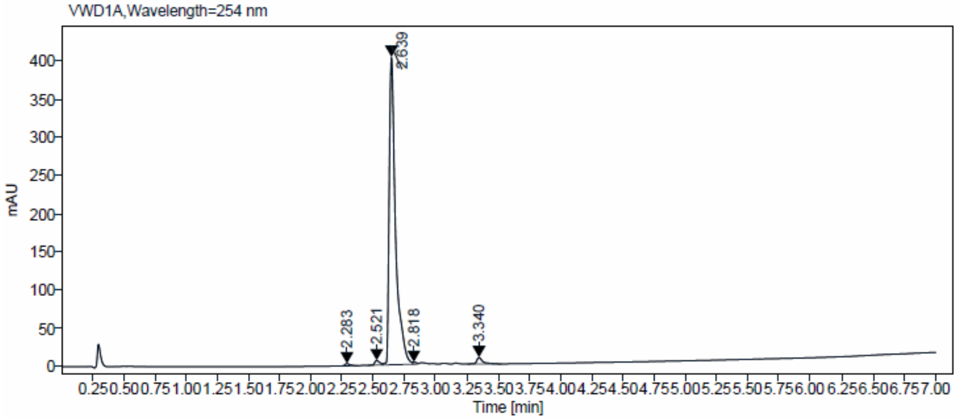

Signal: MS1 +TIC SCAN ESI Frag=110V Gain=1.0

RT [min]	Width [min]	Area	Height	Area%
2.701	0.4499	26636648.7390	4943088.7902	78.1254
3.392	0.4805	2329552.6492	423916.4795	6.8326
5.229	0.5438	510388.1549	32206.3895	1.4970
6.293	0.1014	866111.9764	235770.9640	2.5403
6.358	0.2538	2144771.4700	221155.0744	6.2906
6.676	0.1352	767007.9417	106514.2994	2.2496
6.747	0.2930	840238.0794	132084.6130	2.4644
	**Sum**	**34094719.0105**		

Signal: VWD1A,Wavelength=254 nm

RT [min]	Width [min]	Area	Height	Area%
2.283	0.1514	8.0837	2.8446	0.5422
2.521	0.1185	16.7037	5.8311	1.1203
2.639	0.2408	1432.3272	403.4249	96.0632
2.818	0.0453	5.4958	2.5800	0.3686

Single Injection Report

RT [min]	Width [min]	Area	Height	Area%
3.340	0.2692	28.4148	8.3339	1.9057
	**Sum**	**1491.0253**		

Single Injection Report

**Sample name:**	24		
**Description:**			
**Sample amount:**	0.000	**Sample type:**	Sample
**Instrument:**	LCMS	**Location:**	P2-D7
		**Injection:**	1 of 1
**Acq. method:**	Regular method.amx	**Injection volume:**	5.000 µL
**Analysis method:**	MS method-purity.pmx	**Acq. operator:**	SYSTEM

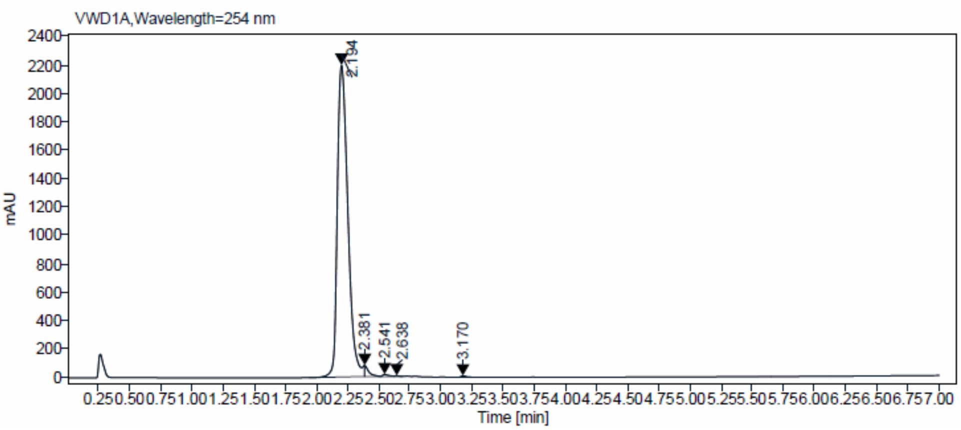

Signal: MS1 +TIC SCAN ESI Frag=110V Gain=1.0

RT [min]	Width [min]	Area	Height	Area%
0.058	0.2480	871322.7376	116134.4248	1.2592
0.315	0.0948	1621431.4177	455013.9436	2.3433
0.394	0.2136	1991687.8005	259935.7094	2.8784
0.680	0.3551	1311879.6285	101588.9255	1.8959
2.263	0.5653	55308641.3425	6190220.5239	79.9320
2.605	0.1998	1544253.6551	204061.3671	2.2318
3.222	0.3576	833667.0274	153686.6017	1.2048
5.060	0.3070	545786.4133	44997.1049	0.7888
5.891	0.1990	513668.9212	73745.0420	0.7424
6.298	0.1175	2265035.6614	595990.7646	3.2734
6.368	0.2704	1735614.7177	321008.0100	2.5083
6.771	0.2253	651634.4595	111631.9173	0.9417
	**Sum**	**69194623.7824**		

Single Injection Report

Signal: VWD1A,Wavelength=254 nm

RT [min]	Width [min]	Area	Height	Area%
2.194	0.4469	12883.8974	2193.5997	97.9610
2.381	0.1266	171.9804	76.8781	1.3076
2.541	0.1064	47.2689	13.8220	0.3594
2.638	0.0709	15.2848	5.5491	0.1162
3.170	0.1814	33.6365	10.4260	0.2558
	**Sum**	**13152.0680**		

Single Injection Report

**Sample name:**	25		
**Description:**			
**Sample amount:**	0.000	**Sample type:**	Sample
**Instrument:**	LCMS	**Location:**	P2-D8
		**Injection:**	1 of 1
**Acq. method:**	Regular method.amx	**Injection volume:**	5.000 µL
**Analysis method:**	MS method-purity.pmx	**Acq. operator:**	SYSTEM

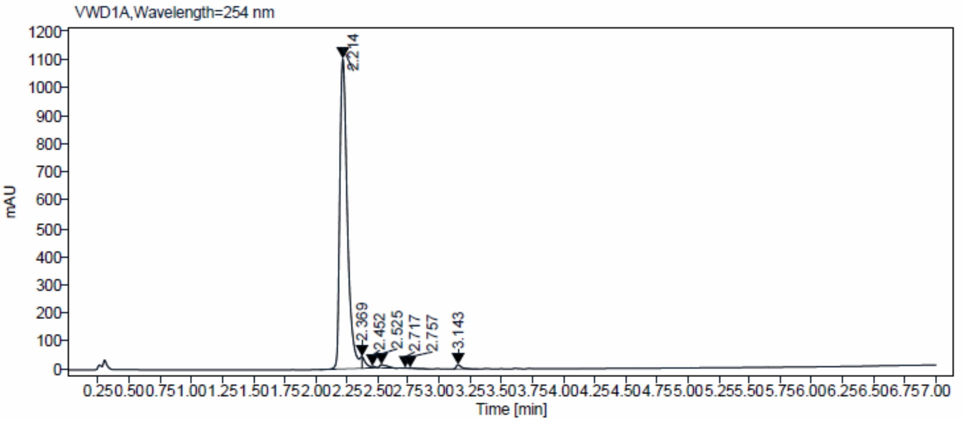

Signal: MS1 +TIC SCAN ESI Frag=110V Gain=1.0

RT [min]	Width [min]	Area	Height	Area%
0.307	0.7031	1372484.8628	207658.5734	3.1328
2.272	0.3120	26251311.2129	4381375.5150	59.9202
2.416	0.1401	2298128.1385	543343.6262	5.2456
2.603	0.1780	936216.2766	159358.4809	2.1370
3.210	0.1780	788686.6582	195422.8023	1.8002
5.898	0.2667	519193.5464	83058.3464	1.1851
6.172	0.1356	672430.9217	186413.7443	1.5349
6.301	0.3829	8247415.4518	1101961.4753	18.8252
6.696	0.1022	993943.0420	198069.9833	2.2687
6.755	0.2891	1730608.8166	218318.4218	3.9502
	**Sum**	**43810418.9275**		

Single Injection Report

Signal: VWD1A,Wavelength=254 nm

RT [min]	Width [min]	Area	Height	Area%
2.214	0.3422	4693.3309	1100.4721	95.8860
2.369	0.0682	78.9880	41.0528	1.6137
2.452	0.0586	14.0691	6.2309	0.2874
2.525	0.1501	46.9003	11.2329	0.9582
2.717	0.0553	5.7085	2.3674	0.1166
2.757	0.1719	8.8371	2.8301	0.1805
3.143	0.2001	46.8659	14.7676	0.9575
	**Sum**	**4894.6999**		

Single Injection Report

**Sample name:**	26		
**Description:**			
**Sample amount:**	0.000	**Sample type:**	Sample
**Instrument:**	LCMS	**Location:**	P2-D9
		**Injection:**	1 of 1
**Acq. method:**	Regular method.amx	**Injection volume:**	5.000 µL
**Analysis method:**	MS method-purity.pmx	**Acq. operator:**	SYSTEM

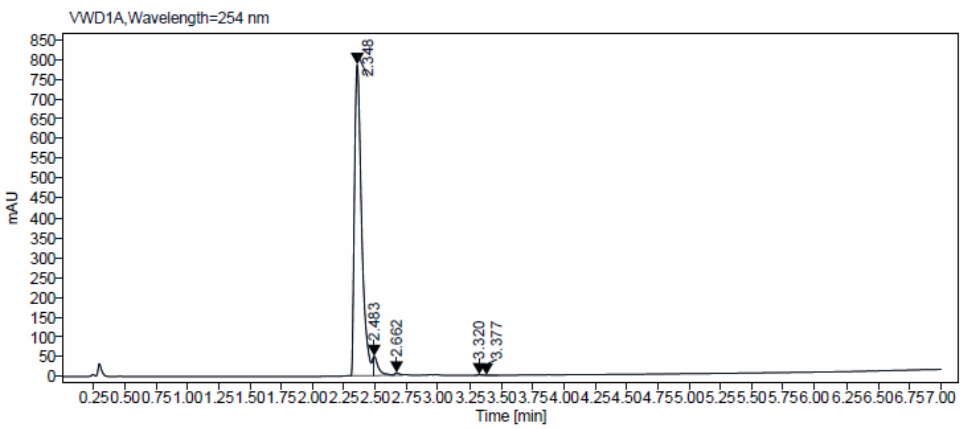

Signal: MS1 +TIC SCAN ESI Frag=110V Gain=1.0

RT [min]	Width [min]	Area	Height	Area%
2.406	0.4375	33675574.1806	4819524.6051	88.1655
2.721	0.2283	1501545.0618	184233.6181	3.9312
2.992	0.3391	904312.9452	80461.5360	2.3676
6.299	0.1061	591047.3804	179586.0461	1.5474
6.367	0.1640	862800.3237	149651.1931	2.2589
6.759	0.1501	660596.4994	137394.4402	1.7295
	**Sum**	**38195876.3911**		

Signal: VWD1A,Wavelength=254 nm

RT [min]	Width [min]	Area	Height	Area%
2.348	0.1820	3149.5236	789.3172	95.5076
2.483	0.1532	127.4137	48.6814	3.8638
2.662	0.0766	14.7992	5.8240	0.4488
3.320	0.0968	5.2101	1.7438	0.1580
3.377	0.0933	0.7229	0.3559	0.0219
	**Sum**	**3297.6695**		

Single Injection Report

**Sample name:**	27		
**Description:**			
**Sample amount:**	0.000	**Sample type:**	Sample
**Instrument:**	LCMS	**Location:**	P2-D10
		**Injection:**	1 of 1
**Acq. method:**	Regular method.amx	**Injection volume:**	5.000 µL
**Analysis method:**	MS method-purity.pmx	**Acq. operator:**	SYSTEM

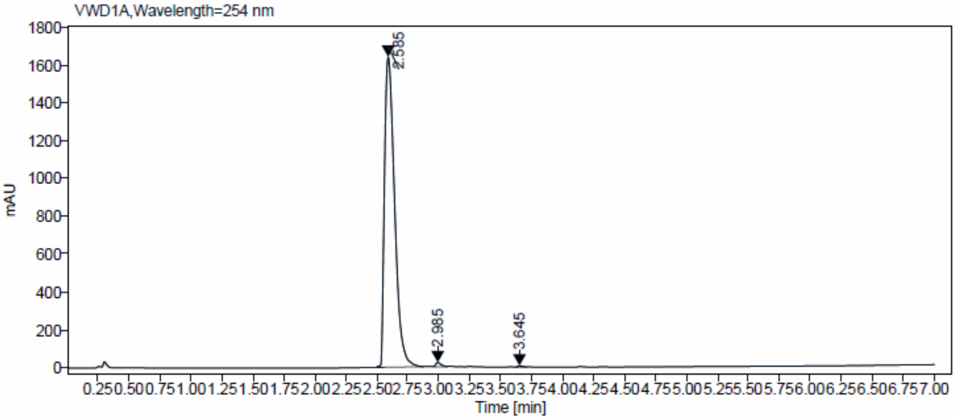

Signal: MS1 +TIC SCAN ESI Frag=110V Gain=1.0

RT [min]	Width [min]	Area	Height	Area%
0.307	0.2293	598145.2913	145077.3205	0.7465
2.654	0.6001	70557121.8515	7846449.6895	88.0575
3.044	0.1214	2118262.7582	478176.6235	2.6437
3.133	0.0960	619510.9479	182057.5128	0.7732
3.305	0.2373	863738.0492	109962.0286	1.0780
3.697	0.3128	1142720.3756	232905.2419	1.4262
6.296	0.3166	3359870.5115	388160.0907	4.1932
6.756	0.2332	866835.9904	106788.8729	1.0818
	**Sum**	**80126205.7756**		

Signal: VWD1A,Wavelength=254 nm

RT [min]	Width [min]	Area	Height	Area%
2.585	0.3871	9328.9464	1643.5412	99.0832
2.985	0.1108	66.3961	22.2443	0.7052
3.645	0.0959	19.9214	7.1372	0.2116

Single Injection Report

RT [min]	Width [min]	Area	Height	Area%
	Sum	9415.2639		

Single Injection Report

**Sample name:**	28		
**Description:**			
**Sample amount:**	0.000	**Sample type:**	Sample
**Instrument:**	LCMS	**Location:**	P2-D11
		**Injection:**	1 of 1
**Acq. method:**	Regular method.amx	**Injection volume:**	5.000 µL
**Analysis method:**	MS method-purity.pmx	**Acq. operator:**	SYSTEM

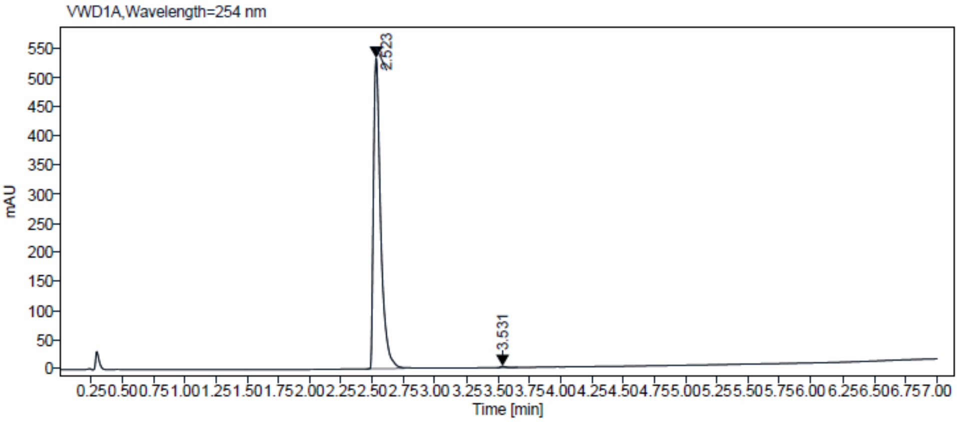

Signal: MS1 +TIC SCAN ESI Frag=110V Gain=1.0

RT [min]	Width [min]	Area	Height	Area%
2.587	0.7314	26993686.2742	4556814.6486	82.1979
6.296	0.1013	977608.9614	262069.1127	2.9769
6.369	0.2615	2710177.5710	278773.6220	8.2527
6.664	0.1852	1536683.3124	165001.4506	4.6793
6.794	0.2013	621700.0346	133605.7669	1.8931
	**Sum**	**32839856.1537**		

Signal: VWD1A,Wavelength=254 nm

RT [min]	Width [min]	Area	Height	Area%
2.523	0.3064	2126.8519	532.9993	99.6719
3.531	0.1698	7.0021	2.2759	0.3281
	**Sum**	**2133.8540**		

Single Injection Report

**Sample name:**	29		
**Description:**			
**Sample amount:**	0.000	**Sample type:**	Sample
**Instrument:**	LCMS	**Location:**	P2-B10
		**Injection:**	1 of 1
**Acq. method:**	Regular method.amx	**Injection volume:**	5.000 µL
**Analysis method:**	MS method-purity.pmx	**Acq. operator:**	SYSTEM

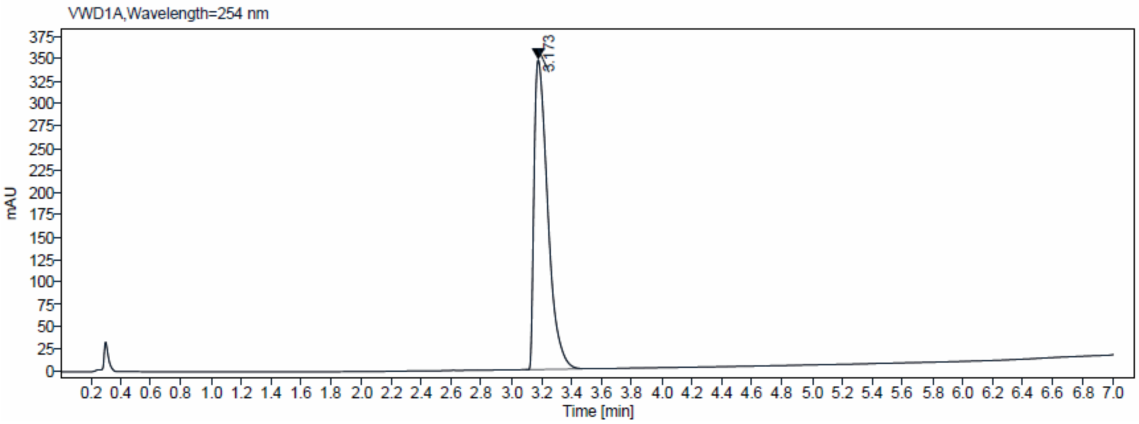

Signal: MS1 +TIC SCAN ESI Frag=110V Gain=1.0

RT [min]	Width [min]	Area	Height	Area%
0.320	1.1752	1331438.7500	157320.5470	3.7542
3.217	0.5605	28465782.1277	3919253.9635	80.2635
6.290	0.1164	1200685.8752	269552.8108	3.3855
6.334	0.2905	2906307.7860	265887.4942	8.1948
6.739	0.3745	1561186.8443	125019.4916	4.4020
	**Sum**	**35465401.3832**		

Signal: VWD1A,Wavelength=254 nm

RT [min]	Width [min]	Area	Height	Area%
3.173	0.3774	2192.8143	346.8816	100.0000
	**Sum**	**2192.8143**		

Single Injection Report

**Sample name:**	30		
**Description:**			
**Sample amount:**	0.000	**Sample type:**	Sample
**Instrument:**	LCMS	**Location:**	P2-C10
		**Injection:**	1 of 1
**Acq. method:**	Regular method.amx	**Injection volume:**	5.000 µL
**Analysis method:**	MS method-purity.pmx	**Acq. operator:**	SYSTEM

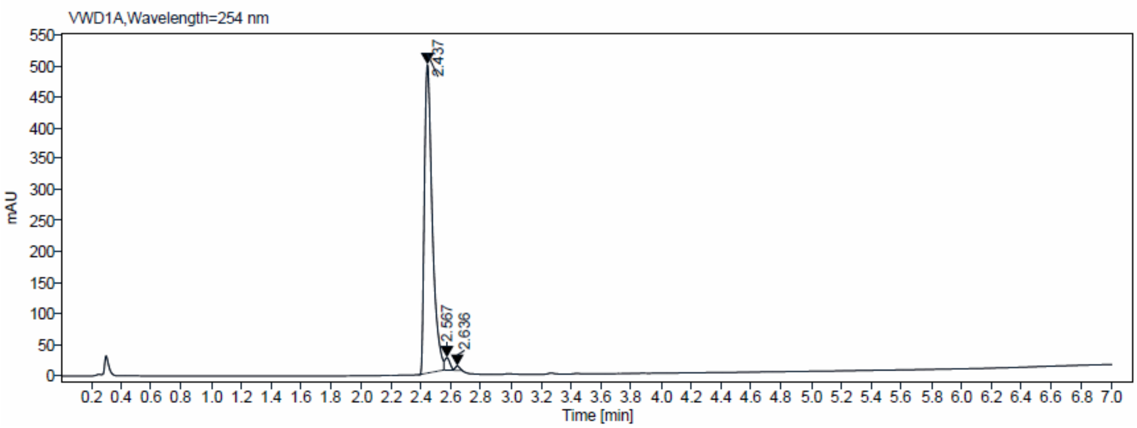

Signal: MS1 +TIC SCAN ESI Frag=110V Gain=1.0

RT [min]	Width [min]	Area	Height	Area%
0.313	0.3577	861864.3440	121869.2915	3.6871
2.502	0.6304	19655220.1733	3203503.1141	84.0863
6.294	0.1098	925344.8812	241089.0519	3.9587
6.341	0.2671	1400042.7564	182594.6793	5.9895
6.739	0.2138	532584.1405	75575.1489	2.2784
	**Sum**	**23375056.2954**		

Signal: VWD1A,Wavelength=254 nm

RT [min]	Width [min]	Area	Height	Area%
2.437	0.1721	1821.8054	498.5783	96.6551
2.567	0.0641	48.5140	19.9239	2.5739
2.636	0.0588	14.5319	7.0058	0.7710
	**Sum**	**1884.8513**		
